# Harnessing the Intrinsic Chemical Reactivity of the Mycotoxin Patulin for Immunosensing

**DOI:** 10.1021/acs.analchem.4c01631

**Published:** 2024-07-15

**Authors:** Hadyn Duncan, Consuelo Agulló, Josep V. Mercader, Antonio Abad-Somovilla, Antonio Abad-Fuentes

**Affiliations:** † Institute of Agricultural Chemistry and Food Technology (IATA), Spanish Scientific Research Council (CSIC), Av. Agustí Escardino 7, Paterna 46980, Valencia, Spain; ‡ Department of Organic Chemistry, University of Valencia, Doctor Moliner 50, Burjassot 46100, Valencia, Spain

## Abstract

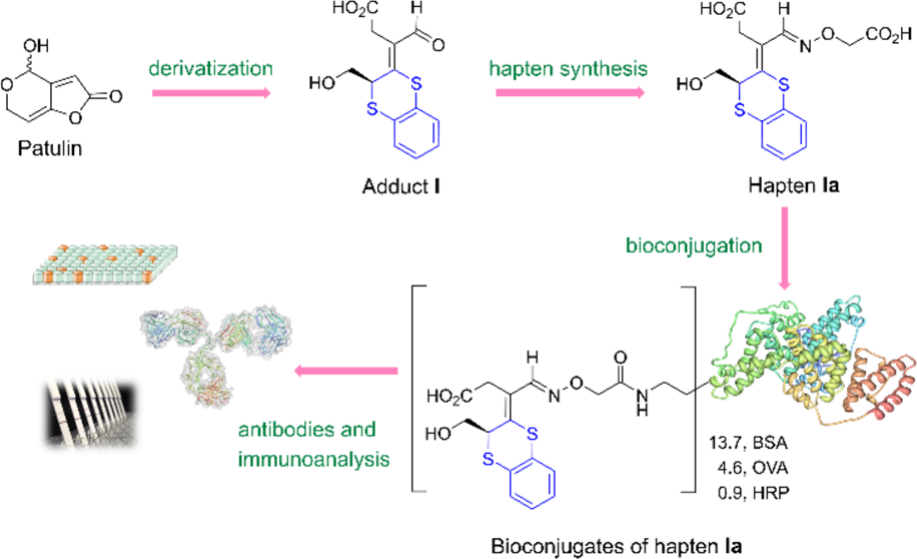

Mycotoxins are globally pervasive contaminants that threaten food safety worldwide. Regulatory authorities have established maximum permissible levels for certain mycotoxins, and their presence is routinely monitored throughout the food chain to ensure the provision of healthy food and safe feed for humans and animals. While immunoanalytical methods are essential for mycotoxin screening, monoclonal antibodies for the detection of patulin are notably absent. Moreover, leading immunodiagnostic companies currently do not offer rapid tests for patulin in their product portfolios. This deficiency in mycotoxin testing is primarily due to the electrophilic reactivity of patulin. In this study, we exploit this reactivity to develop an innovative strategy that targets the stable adduct formed by the reaction of patulin with aryl-1,2-dithiolates, rather than analyzing the mycotoxin itself. Based on this previously unknown reaction, we present the first collection of monoclonal antibodies, enabling the long-sought goal of sensitive, simple, and user-friendly immunosensing of patulin.

Mycotoxins are low-molecular-weight, nonvolatile toxic secondary metabolites produced by numerous fungal species that contaminate grains, nuts, and fruits.^1^ The presence of these toxins in food and feed poses significant threats to human health and animal welfare and results in substantial economic losses for the agri-food industry.^2^ Therefore, monitoring the presence of mycotoxins throughout the food chain is a top priority for food safety authorities worldwide. To mitigate the risks associated with mycotoxins, maximum allowable levels have been established internationally for both raw materials and processed foods. Although hundreds of mycotoxins have been identified to date, only a limited group—including aflatoxins, ochratoxin A, fumonisins, deoxynivalenol, zearalenone, and patulin—is subject to international regulation.^3−6^ Analytical methods are essential tools for regulatory agencies to ensure high standards of food and feed safety. Alongside a variety of analytical methods,^7−9^ antibody-based approaches are widely employed in the agri-food industry and by official laboratories for the analysis of regulated mycotoxins. Consequently, rapid tests for these compounds are marketed by numerous food immunodiagnostic companies.^10^ The only exception to this comprehensive testing framework is patulin, a mycotoxin produced primarily by *Penicillium expansum*, a mold that predominantly infects fruits, particularly apples.^11,12^

Previous efforts to generate specific and high-affinity antibodies against patulin have not proven effective for use in immunoassay formats commonly employed in immunodiagnostics, such as enzyme-linked immunosorbent assays (ELISA) and lateral flow immunoassays (LFIA).^13−17^ In addition, monoclonal antibodies, which are considered the gold standard among immunoreagents in commercial assays for mycotoxin detection, have not yet been reported for patulin. Although the small size and rather limited structural complexity of patulin are certainly two limiting factors underlying the difficulty to develop powerful and robust antibodies against this mycotoxin, our group has recently demonstrated that the primary challenge lies in the high electrophilic reactivity of the patulin molecule, which has hindered the development of sensitive, simple, and user-friendly immunoanalytical methods for its detection.^18^ In that study, we also proposed a strategy to overcome the immunogenic challenges posed by patulin conjugates in eliciting an effective immune response. This strategy involved targeting the adduct formed when patulin reacts with a thiol-containing aromatic compound rather than the mycotoxin itself. If antibodies could be raised against these adducts, it might be possible to analyze patulin-contaminated samples following a simple derivatization reaction, a strategy previously employed for other simple chemicals such as glyphosate and acrylamide.^22,23^ While this approach showed promise, it was not entirely satisfactory due to the limited affinity of the antibodies obtained against these derivatives. This limitation could be attributed to two main factors: (i) the high flexibility of the compounds proposed as haptens for eliciting the immune response and (ii) the derivatization reaction, although primarily producing a major adduct, also resulted in the formation of other side products, thereby compromising the overall strategy.

In this context, the aim of this study was to investigate alternative chemical strategies that could exploit the electrophilic reactivity of patulin to generate high-affinity monoclonal antibodies. These antibodies would ultimately facilitate the rapid and on-site analysis of mycotoxin in potentially contaminated foods.

## Experimental Section

### Reagents and Instruments

Chemicals and apparatus, as well as general experimental techniques, are described in the Supporting Information. The file also includes the preparation of adducts other than adduct **I**, the preparation of bioconjugates of adduct **I**, the matrix-assisted laser desorption/ionization-time-of-flight-mass spectrometry (MALDI-TOF-MS) analysis of bioconjugates, the evaluation of the immune response in rabbits, and the production of monoclonal antibodies.

### Reaction of Patulin with Benzene-1,2-dithiol: Preparation of Adduct **I**

#### Preparation of Disodium Benzene-1,2-thiolate

Benzene-1,2-dithiol (200 mg, 1.06 mmol) was added to a solution of sodium *tert*-butoxide (325 mg, 3.38 mmol, 2.4 equiv) in anhydrous methanol (1.5 mL) under nitrogen, and the mixture was stirred at 50 °C for 1 h. The resulting mixture was cooled to rt and concentrated at reduced pressure, and the obtained solid residue was washed with anhydrous THF (2×) and Et_2_O (2×). The resulting white solid was dried under high vacuum overnight to give disodium benzene-1,2-thiolate (229.4 mg, 88%) as a white solid that was used directly without further purification. ^1^H NMR (300 MHz, D_2_O) δ (ppm) 7.41 (dd, *J* = 5.8, 3.5 Hz, 2H, H-3 and H-6), 6.70 (dd, *J* = 5.8, 3.6 Hz, 2H, H-4 and H-5).^21^

#### Reaction of Patulin with Disodium Benzene-1,2-thiolate

A solution of the obtained (bis)thiolate (13.3 mg, 0.071 mmol, 1.1 equiv) in Milli-Q water (5 mL) was added into a stirred solution of patulin (10.0 mg, 0.065 mmol) in PB (5 mL) at rt. After 20 min of stirring, the resulting yellowish mixture was cooled in an ice–water bath, acidified with HCO_2_H to pH 3–4, and extracted with EtOAc (20 mL × 3). The combined organic phases were washed with brine (15 mL), dried over anhydrous MgSO_4_, and concentrated under reduced pressure to give adduct **I** (18.0 mg, 93.7%) as a yellow oil. The crude product showed a purity ≥95% as judged by ^1^H NMR (see Figure S2) and was not further purified. IR v_max_ (cm^–1^) 3447m, 2924m, 1783m, 1725s, 1654s, 1558m, 1451m, 1200s, 1062s; ^1^H NMR (300 MHz, CDCl_3_) δ ppm 10.26 (s, 1H, HCO), 7.32–7.24 (m, 2H, H-5′and H-8′), 7.20–7.14 (m, 2H, H-6′and H-7′), 5.75 (br s, 2H, 2xOH), 4.30 (t, *J* = 7.4 Hz, 1H, H-3′), 3.73 (dd, *J* = 11.6, 7.7 Hz, 1H, *CH*OH), 3.64 (dd, *J* = 11.6, 7.4 Hz, 1H, *CH‘*OH), 3.624 and 3.67 (each d, AB system, *J* = 16.8 Hz, 1H each, H-2); ^13^C NMR (126 MHz, acetone-*d*_6_) δ (ppm) 188.7 (CH, C-4), 171.5 (C-1), 154.0 (C-2′), 131.2 (CH, C-8′), 130.7 (C, C-8′a), 128.6 (C, C-4′a), 127.9 (CH, C-6′), 127.64 and 127.61 (each CH, C-5′ and C-7′), 127.5 (C, C-3), 62.9 (CH_2_OH), 43.7 (CH, C-3′), 32.4 (CH_2_, C-2); high-resolution mass spectrometry (HRMS) *m*/*z* calcd for C_13_H_13_O_4_S_2_ [M + H]^·+^ 297.0250, found [M + H]^·+^ 297.0250.

### Synthesis of (3*Z*,4*E*)-4-((Carboxymethoxy)imino)-3-(3-(hydroxymethyl)benzo[*b*][1,4]dithiin-2(3*H*)-ylidene)butanoic Acid (Hapten **Ia**)

A solution of adduct **I** (17.5 mg, 0.059 mmol), (aminooxy)acetic acid hemihydrochloride (10.3 mg, 0.094 mmol, 1.6 equiv), and dry pyridine (10 μL, 9.8 mg, 0.124 mmol, 2.1 equiv) in a 3:1 (v/v) mixture of absolute EtOH and anhydrous THF (0.5 mL) was stirred at rt for 4 h under nitrogen. The solvents were evaporated under reduced pressure, and the residue was suspended in a cold 1 M aqueous solution of HCl (1 mL) and extracted with EtOAc (10 mL × 3). The combined organic phases were washed with brine (15 mL), dried over anhydrous MgSO_4_, and concentrated on the rotary evaporator to give hapten **Ia** (14 mg, 64.3%) as a yellow film. IR v_max_ (cm^–1^) 3006m, 2926s, 2113w, 1707s, 1628w, 1420m, 1360s, 1220s, 1092s; ^1^H NMR (500 MHz, acetone-*d*_6_) δ (ppm) 8.54 (s, 1H, H-4), 7.31 (dd, *J* = 7.9, 1.5 Hz, 1H, H-5′), 7.29 (dd, *J* = 7.7, 1.7 Hz, 1H, H-8′), 7.19 (td, *J* = 7.6, 1.7 Hz, 1H, H-6′), 7.15 (td, *J* = 7.4, 1.5 Hz, 1H, H-7′), 4.66 (s, 2H, NOCH_2_), 4.51 (dd, *J* = 7.9, 6.8 Hz, 1H, H-3′), 3.71 (dd, *J* = 11.1, 7.9 Hz, 1H, *CH*OH), 3.70 and 3.65 (each d, AB system, *J* = 16.9 Hz, 1H each, H-2), 3.60 (dd, *J* = 11.1, 6.8 Hz, 1H, *CH’*OH); ^13^C NMR (126 MHz, acetone-*d*_6_) δ (ppm) 171.4 (C, C-1), 170.7 (CO_2_H), 148.5 (CH, C-4), 140.0 (C, C-2′), 130.95 (CH, C-8′), 130.9 (C, C-8′a), 128.4 (C, C-4′a), 127.5 (CH, C-5′), 127.3 (CH, C-6′), 127.1 (CH, C-7′), 121.8 (C, C-3), 71.4 (NOCH_2_), 63.5 (CH_2_OH), 43.4 (CH, C-3′), 33.7 (CH_2_, C-2); HRMS calcd for C_15_H_16_NO_6_S_2_ [M + H]^·+^ 370.0414, found [M + H]^·+^ 370.0420.

### Synthesis of (3*Z*,4*E*)-4-(((5-Azidopentyl)oxy)imino)-3-(3-(hydroxymethyl)benzo[*b*][1,4]dithiin-2(3*H*)-ylidene)butanoic Acid (Hapten **Ib**)

A solution of adduct **I** (10.9 mg, 0.038 mmol), hydroxylamine-azide **8** prepared as described in the Supporting Information (6.8 mg, 0.047 mmol, 1.25 equiv), and dry pyridine (6.2 μL, 0.077 mmol, 2.1 equiv) in absolute EtOH (0.3 mL) was stirred at rt for 3 h under nitrogen. Then, the solvents were removed under reduced pressure, and the residue was treated with 1 mL of a 1 M cold aqueous solution of HCl (1 mL) and extracted with EtOAc (10 mL × 3). The combined organic phases were washed with brine (15 mL), dried over anhydrous MgSO_4_, and concentrated under reduced pressure. The obtained residue was purified by flash silica gel chromatography, using a 98:2 mixture of CHCl_3_-MeOH as the eluent, to provide the hapten **Ib** (9.8 mg, 61%) as a clear oil. IR v_max_ (cm^–1^) 3181m, 2939s, 2871m, 2096s, 1707s, 1455m, 1422m, 1217s, 1187s, 1108s, 1049s, 933s; ^1^H NMR (300 MHz, acetone-*d*_6_) δ (ppm) 8.44 (s, 1H, H-4), 7.28 (m, 2H, H-5′ and H-8′), 7.17 (m, 2H, H-6′ and H-7′), 4.52 (t, *J* = 7.3 Hz, 1H, H-3′), 4.10 (t, *J* = 6.5 Hz, 2H, H-1″), 3.73 and 3.66 (each d, AB system, *J* = 16.9 Hz, 1H each, H-2), 3.70 (dd, *J* = 11.1, 7.8 Hz, 1H, *CH*OH), 3.59 (dd, *J* = 11.1, 6.9 Hz, 1H, *CH’*OH), 3.35 (t, *J* = 6.8 Hz, 2H, H-5″), 2.82 (br s, 2H, 2xOH), 1.77–1.58 (m, 4H, H-2″ and H-4″), 1.52–1.41 (m, 2H, H-3″); ^13^C NMR (126 MHz, acetone-*d*_6_) δ (ppm) 171.7 (C, C-1), 146.9 (CH, C-4), 138.4 (C, C-2′), 131.1 (C, C-8′a), 130.9 (CH, C-8′), 128.4 (C, C-4′a), 127.5 (CH, C-6′), 127.3 (CH, C-7′), 127.0 (CH, C-5′), 122.4 (C, C-3), 74.8 (CH_2_, C-1″), 63.5 (CH_2_OH), 51.9 (CH_2_, C-5″), 43.5 (CH, C-3′), 33.8 (CH_2_, C-2), 29.5 and 29.4 (each CH_2_, C-2″ and C-4,″ overlapped by solvent signal), 23.8 (CH_2_, C-3″); HRMS *m*/*z* calcd for C_18_H_23_N_4_O_4_S_2_ [M + H]^·+^ 423.1155, found [M + H]^·+^ 423.1149.

### Preparation of 2,5-Dioxopyrrolidin-1-yl (3*Z*,4*E*)-4-((2-((2-(l2-Azaneylidene)-5-oxopyrrolidin-1-yl)oxy)-2-oxoethoxy)imino)-3-(3-(hydroxymethyl)benzo[*b*][1,4] Dithiin-2(3*H*)-ylidene)butanoate (*bis*-NHS Ester of Hapten **Ia**)

Hapten **Ia** (13.4 mg, 0.036 mmol), EDC·HCl (21.6 mg, 0.112 mmol, 3.1 equiv), and NHS (12.8 mg, 0.112 mmol, 3.1 equiv) were weighed into a reaction vial and purged with nitrogen. The vial was then cooled to −50 °C, and a 2:1 mixture of anhydrous MeCN and THF (1.2 mL) was added. The mixture was stirred for 2 h, keeping the temperature at −40 °C. Then, cold water (5 mL) was added to the vial, and the mixture was extracted with Et_2_O (15 mL x 3). The combined organic phases were washed with brine (15 mL), dried over anhydrous MgSO_4_, and concentrated under vacuum to give the *bis*-NHS ester of hapten **Ia** (10.8 mg, 53%) as a yellowish oil. ^1^H NMR (300 MHz, CDCl_3_) δ (ppm) 8.61 (s, 1H, H-4), 7.32–7.27 (m, 1H, H-8′), 7.24–7.08 (m, 3H, H-5′, H-6′ and H-7′), 5.08 (AB system, *J* = 16.7 Hz, 2H, NOCH_2_), 4.24 (t, *J* = 7.3 Hz, 1H, H-3′), 4.00 and 3.84 (each d, AB system, *J* = 17.3 Hz, 1H each, H-2), 3.72–3.64 (m, 1H, *CH*OH), 3.60 (dd, *J* = 11.8, 7.5 Hz, 1H, *CH’*OH), 2.84 and 2.83 (each s, 4H each, 2xCO*CH*_2_*CH*_2_CO).

### Preparation of Bioconjugates of Hapten **Ia**

For the preparation of the bioconjugate BSA–**Ia**, 136 μL of a 100 mM solution in DMSO of the bis-NHS ester of hapten **Ia** (7.6 mg, 13.5 μmol, *ca.* 60 equiv) was added slowly with stirring to 1.0 mL of a solution of BSA (15 mg/mL) in PB, and the conjugation reaction mixture was stirred for 48 h at rt. Coupling conditions for OVA and HRP were the same, but amounts differed. Thus, for the preparation of OVA–**Ia**, 80 μL of a 50 mM solution in DMSO of the bis-NHS ester of hapten **Ia** (2.25 mg, 4 μmol, *ca.* 35 equiv) was added to 1 mL of a 5 mg/mL solution of OVA, while for the preparation of the bioconjugate HRP–**Ia**, 60 μL of a 20 mM solution in DMSO of the bis-NHS ester of hapten **Ia** (675 μg, 1.2 μmol, *ca.* 20 equiv) was added to 0.9 mL of a solution of HRP (3 mg/mL). The bioconjugates were purified by size-exclusion chromatography and sterilized by filtration before being stored at −20 °C (BSA and OVA) or 4 °C (HRP).

### Preparation of Bioconjugates of Hapten **Ib**

The proteins were first modified with propargyl groups. To this end, the *N*-hydroxysuccinimidyl ester of 5-oxo-5-(prop-2-in-1-ylamino)pentanoic acid (**9**)^19,20^ was dissolved in DMF and added dropwise to a solution of protein in PB. The initial molar ratios of alkyne to protein (RM_0_) used in the reaction mixture were 40, 12, and 10 for BSA, OVA, and HRP, respectively. The conjugation reaction was carried out with a maximum DMF content of 10% (v/v). After incubation overnight at room temperature, the alkylated proteins were purified by size-exclusion chromatography and stored at 4 °C until use.

For the preparation of the bioconjugate BSA–**Ib**, 622.8 μL of a solution of the hapten **Ib** (3.0 mg, 7.1 μmol, *ca*. 2 equiv of hapten per alkyne residue) in DMSO was added dropwise to a 10 mg solution of the BSA-alkyne (0.142 μmol) in 25 mL of PB. Thereafter, 66.5 μL of a solution of THPTA (54.1 mM, 3.55 μmol, 1 equiv per alkyne residue) and CuSO_4_ (10.7 mM, 0.71 μmol, 0.2 equiv per alkyne residue) in Milli-Q H_2_O were added dropwise. The resulting mixture was degassed by vacuum/nitrogen purges (3×), and 150 μL (10 equiv per alkyne residue) of a 230 mM solution of sodium ascorbate in Milli-Q H_2_O, also previously degassed by vacuum/nitrogen purges (3×), was added. The resulting solution was stirred for 22 h at rt, and the resulting BSA–**Ib** bioconjugate was first separated from the rest of the reagents using Amicon Ultra-4 10K filters and then purified by size-exclusion chromatography. The bioconjugate was sterilized by filtration through a 0.45 μm nylon filter and stored at −20 °C.

Coupling conditions for the OVA-alkyne and HRP–alkyne were identical except for the employed reagent amounts. For the preparation of the bioconjugate OVA–**Ib**, OVA–alkyne (1.0 mg/mL, 6 mL, *ca.* 0.12 μmol) in PB, hapten **Ib** (11.4 mM, 153.5 μL, 1.75 μmol, *ca.* 4 equiv per alkyne residue) in DMSO, a solution of THPTA (13.2 mM, 0.88 μmol) and CuSO_4_ (2.71 mM, 0.18 μmol) in MiliQ H_2_O (66.5 μL), and sodium ascorbate in MiliQ H_2_O (230 mM, 40 μL, 8.8 μmol) were mixed. For the preparation of the bioconjugate HRP–**Ib**, HRP–alkyne (1.5 mg/mL, 1.33 mL, 45 nmol) in PB, hapten **Ib** (10 mM, 18 μL, 180 μmol, *ca.* 3 equiv per alkyne residue) in DMSO, a solution of THPTA (0.68 mM, 45 nmol) and CuSO_4_ (0.135 mM, 9 nmol) in MiliQ H_2_O (66.5 μL), and sodium ascorbate in MiliQ H_2_O (10 mM, 45 μL, 450 nmol) were used. After purification, the bioconjugates were kept at −20 °C (OVA–**Ib**) or 4 °C (HRP–**Ib**).

## Results and Discussion

### Patulin Readily Reacts with Aryl-1,2-dithiolates to Afford a Unique Compound

**Scheme 1:**
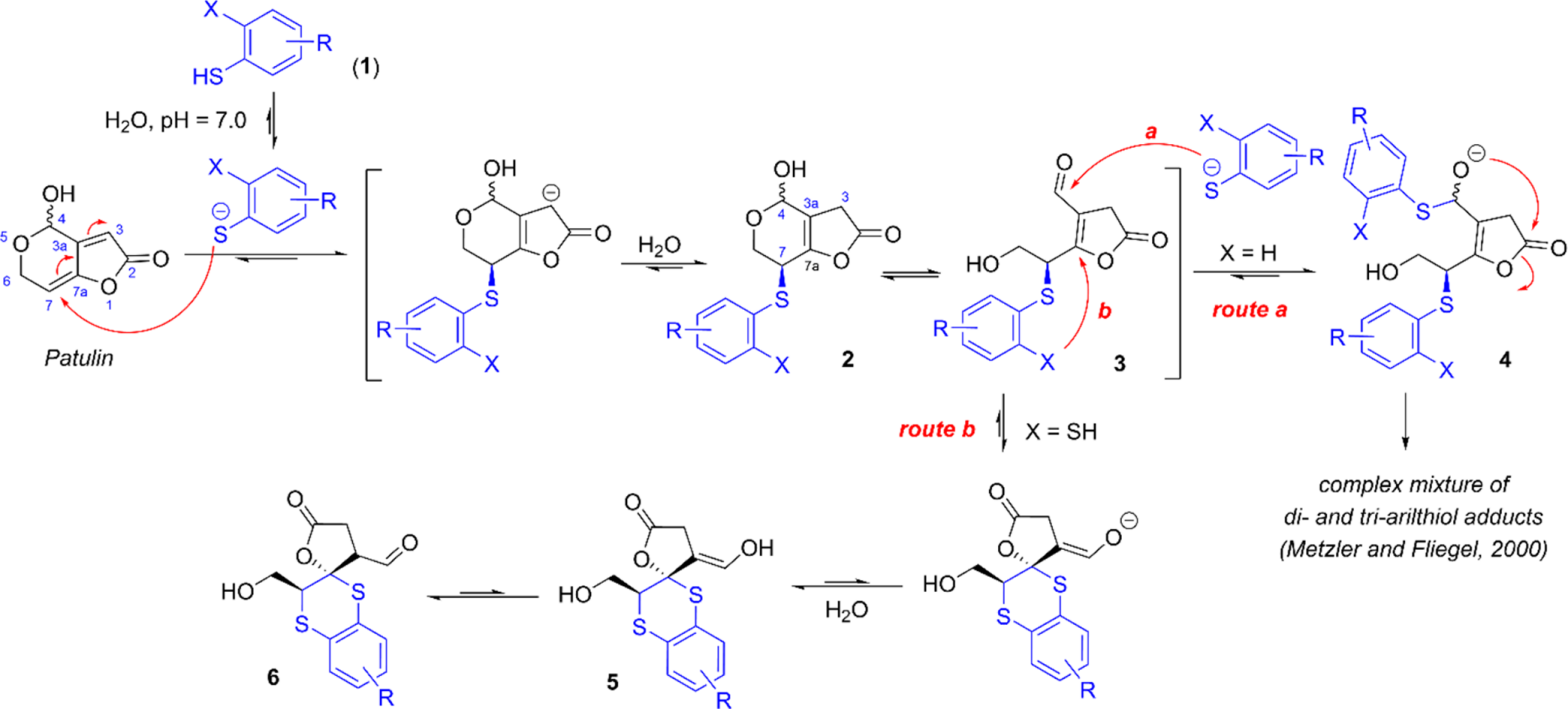
Proposed Pathway for the Addition of Aryl Monothiols (Route a) and Aryl-1,2-dithiols (Route b) to Patulin The addition of the aryl thiol generates a new stereogenic center at C–7, which can have either an *R*– or an *S*–configuration. For simplification, only the stereoisomers with the *S*–configuration at this center are depicted.

Previous studies by Fliege and Metzler, corroborated by our research group, demonstrated that patulin reacts in an aqueous medium with aryl monothiols as nucleophiles via the sodium thiolate (**1**, X = H), resulting in a multitude of products through complex reaction pathways (Scheme 1, route a).^23,24^ The initial step for all observed products involves a thia-Michael addition to the electrophilic C-7 position of the α,β,γ,δ-unsaturated lactone moiety, leading to a transient hemiacetal monoadduct (**2**) that is in equilibrium with the tautomeric ring-opened (hydroxy-aldehyde) form (**3**). In the presence of an excess of thiol, a rapid nucleophilic 1,2-addition to the electrophilic aldehyde carbonyl group occurs, forming a thiohemiacetal intermediate (**4**). This intermediate undergoes a series of rearrangements, eliminations, and even the addition of a new thiol molecule, resulting in a complex mixture of products, an outcome that is far from the desired goal of obtaining a single compound.

We hypothesized that the mechanistic pathway of the addition reaction of an aryl thiol to patulin could be altered and potentially directed toward the formation of a more stable unique adduct by using an aryl-1,2-dithiolate (**1**, X = SNa) instead of an aryl monothiolate. We speculate that after the addition of the first thiolate group to the electrophilic C-7 position of patulin, the presence of the second thiol group could modify the reaction pathway, favoring an intramolecular thia-Michael addition. This reaction could yield a compound such as **6**, which we believed would be stable enough to be isolated as the major product of the dithiolate addition reaction (Scheme 1, route b).

**Scheme 2:**
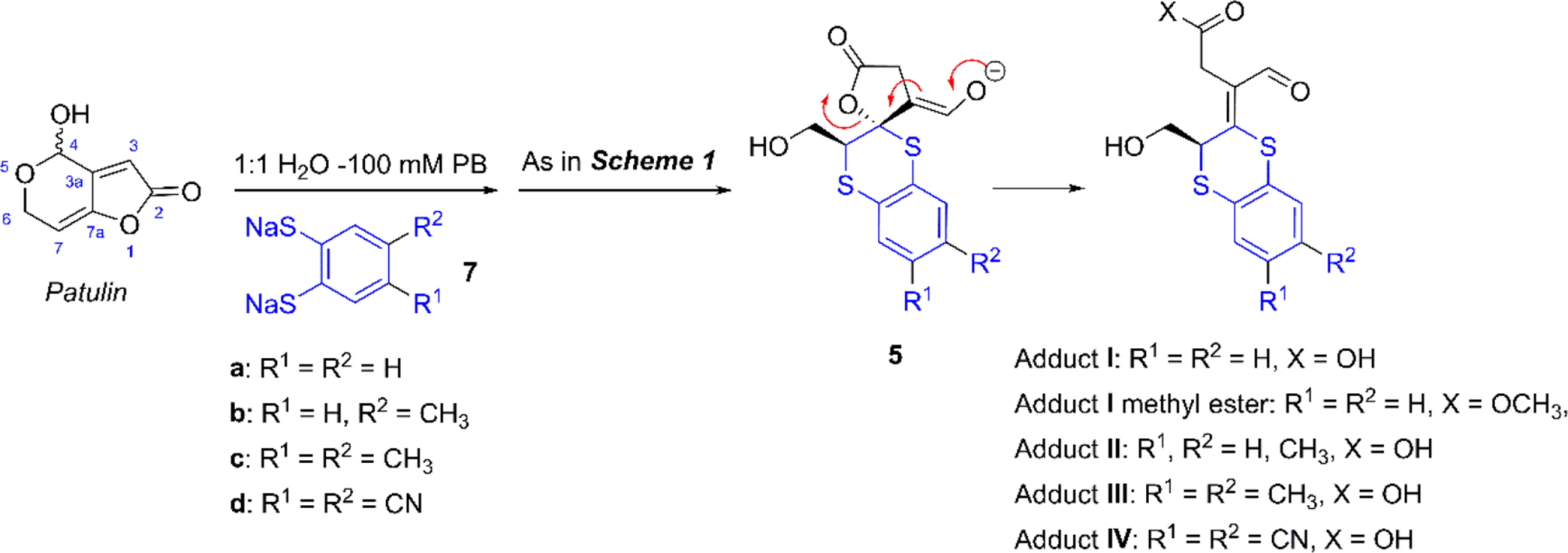
Formation of Monodithiol Adducts (**I**–**IV**) through Addition of Aryl-1,2-dithiolates to Patulin

To test our hypothesis, we first reacted patulin with the disodium salt of commercially available benzene-1,2-dithiol, which was independently and easily prepared by reacting dithiol with NaO*^t^*Bu in methanol. The reaction was conducted at room temperature in 100 mM phosphate buffer, pH = 7.4 (PB). Upon mixing all reactants, an intense yellow color was immediately observed, indicating the reaction of patulin with the dithiol had occurred. After a few minutes and subsequent acidic workup, analysis of the crude reaction mixture *via* both TLC and ^1^H NMR spectroscopy revealed the presence of a predominant reaction product (Figures S1 and S2). Based on spectroscopic data, structure **I** was tentatively assigned to this dithiol adduct, which forms following the ring opening of the γ-lactone moiety of the initially formed intermediate **5** (Scheme 2). The final confirmation of the structure and stereochemistry of this adduct was achieved by converting it to the corresponding methyl ester through esterification with diazomethane, followed by chromatographic purification and comprehensive spectroscopic analysis, including two-dimensional (2D) NMR techniques such as COSY, HSQC, HMBC, and NOESY experiments (Table S1, Figure S3, and NMR spectra in the Supporting Information).

The reaction of patulin with other sodium aryl-1,2-dithiolates proceeded similarly, primarily yielding the corresponding monodithiol adducts (Scheme 2). This outcome was particularly notable with aryl-1,2-dithiols possessing electron donor substituents in the *meta*/*para* positions relative to the thiol groups. For instance, when patulin was reacted with the sodium salt of 4-methyl-1,2-benzenedithiol (**7b**), a 1:1 mixture of the regioisomeric adducts **II** was obtained. Similarly, the reaction with the sodium salt of 4,5-dimethyl-1,2-benzenedithiol (**7c**) resulted in the formation of adduct **III**. Conversely, the efficiency of monodithiol adduct formation was markedly lower with the sodium salt of 4,5-dicyano-1,2-benzenedithiol (**7d**), likely due to the lower nucleophilic nature of this thiolate owing to the electron-withdrawing properties of the cyano groups. Interestingly, reactions involving alkyl-1,2-dithiols, such as 1,2-ethanedithiol, also predominantly produced the corresponding monodithiol adduct, albeit with less clean reaction profiles compared to aryl counterparts.

### Patulin Adducts are Efficiently Conjugated to Proteins and Trigger an Effective Immune Response

The efficiency of forming patulin-monodithiol adducts, combined with the simplicity of the experimental conditions and the rigidity and functional diversity of the resulting derivatives, paved the way for the production of antibodies and the development of rapid immunoanalytical methods based on the prior derivatization of patulin with an aryl-1,2-dithiolated compound. Notably, this reaction can be performed with the same efficiency and cleanliness using a large excess of the dithiolate or extended reaction times, demonstrating the stability of these adducts in an aqueous medium. This stability is a crucial feature for ensuring reliable antibody production and a consistent analytical performance.

With this goal in mind, we focused on adduct **I**, due to both the efficacy with which it is formed and the commercial availability of the corresponding dithiol. Adduct **I** was first coupled to immunogenic (bovine serum albumin, BSA) and coating (ovalbumin, OVA) carrier proteins by carbodiimide-mediated chemistry, taking advantage of the carboxyl group of the hapten. Using MALDI-TOF mass spectrometry, coupling densities of 6.0 and 4.5 were determined for BSA and the OVA bioconjugates, respectively (Figures S4 and S6). To evaluate the immunogenicity of the BSA–adduct conjugate, rabbits were immunized, and the resulting antisera were tested by indirect competitive ELISA. Unfortunately, low antibody titers were obtained, and poor recognition of free adduct **I** was observed. This result, while disappointing, was not entirely unexpected and could be attributed to the relatively low hapten loading in the immunogen and the presence of an aldehyde group in the adduct, which may be modified *in vivo* after inoculation—it is known that aldehyde groups can form Schiff base adducts with α- and γ-amino groups of proteins.^25−27^ To mitigate this issue and improve the exposure of the adduct backbone to the immune system, we decided to incorporate a spacer arm through the aldehyde carbonyl group *via* the formation of an oxyimino bond.

**Scheme 3:**
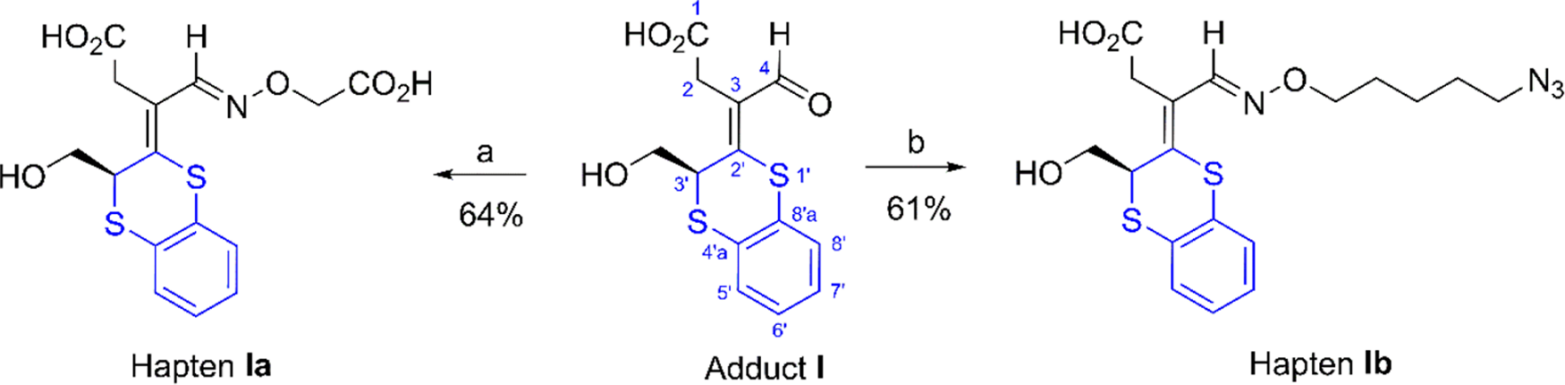
Preparation of Haptens **Ia** and **Ib** Reagents and conditions: (a) HO_2_CCH_2_ONH_2_·1/2HCl, pyridine, EtOH-THF, rt, 4 h; (b) N_3_(CH_2_)_5_ONH_2_ (**8**), pyridine, EtOH, rt, 3 h.

Accordingly, we designed two derivatives of adduct **I** for immunization, namely, haptens **Ia** and **Ib** (Scheme 3). Hapten **Ia** contains a short chain with a (carboxymethoxy)imino moiety with a terminal carboxyl group for conjugation to carrier proteins by the formation of the corresponding active ester. Hapten **Ib**, on the other hand, features a longer linear saturated hydrocarbon chain with a terminal azide group. This azide group possesses orthogonal reactivity with respect to the other functional groups of the hapten, facilitating chemoselective conjugation to carrier proteins *via* click chemistry through a Cu(I)-catalyzed 1,3-dipolar azide–alkyne cycloaddition (CuAAC) reaction,^28,29^ a coupling strategy that our group has successfully employed for other compounds.^30,31,32^ The synthesis of hapten **Ia** was accomplished in 64% yield by reacting adduct **I** with (aminooxy)acetic acid hemihydrochloride and pyridine in a mixture of ethanol and tetrahydrofuran (Scheme 3). Similarly, hapten **Ib** was synthesized in 61% yield using *O*-(5-azidopentyl)hydroxylamine (**8**) as a nucleophile, which was prepared in three steps from 1,5-dibromopentane (Supporting Information).

**Scheme 4:**
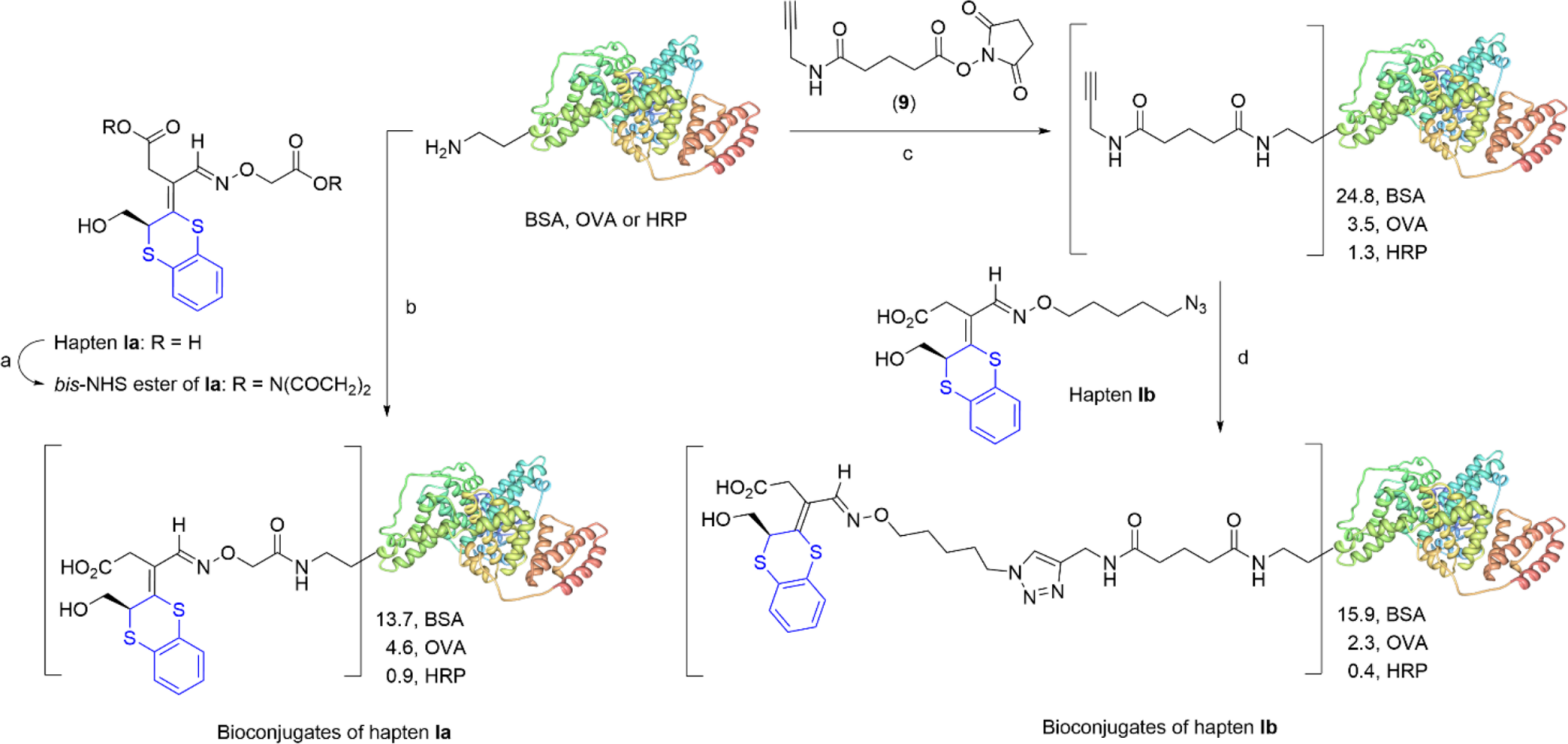
Preparation of Bioconjugates of Haptens **Ia** and **Ib** Reagents and conditions: (a) EDC·HCl (3 equiv), NHS (3 equiv), 2:1 CH_3_CN-THF, −40 °C, N_2_, 2 h (53%); (b) DMSO-PB 100 mM (pH 7.4), rt, 48 h; (c) DMF-PB 100 mM (pH 7.4), rt, overnight; (d) 5:1 CuSO_4_–THPTA complex, sodium ascorbate, DMSO-PB 100 mM (pH 7.4), rt, N_2_, 22 h.

With both haptens at hand, we first activated the carboxyl group of hapten **Ia** at the (carboxymethoxy)imino moiety by forming the corresponding *N*-hydroxysuccinimidyl ester using 1.4 equiv of both 1-ethyl-3–3-(dimethylamino)propyl carbodiimide hydrochloride (EDC·HCl) and *N*-hydroxysuccinimide (NHS). Unlike the direct activation of adduct **I** described earlier, the use of standard temperatures (0–25 °C) resulted in a very complex mixture of products. Only when the reaction was carried out at temperatures below −25 °C did the reaction proceed as expected, although the NHS ester of the C-1 carboxyl group and the bis-NHS ester were also observed. To avoid working with product mixtures, we opted to prepare only the bis-NHS ester of hapten **Ia** using 3 equiv of both EDC-HCl and NHS, with a reaction time of 48 h. We hypothesized that under standard conjugation conditions in buffer, coupling of the bis-NHS ester of hapten **Ia** to carrier proteins would occur preferentially through the (carboxymethoxy)imino group, while the NHS ester at C-1 would hydrolyze to regenerate the carboxyl group at this position. The bioconjugates prepared in this way were characterized by MALDI-TOF-MS analysis, which determined hapten densities of 13.7, 4.6, and 0.9 for the conjugates BSA–**Ia**, OVA–**Ia**, and HRP–**Ia**, respectively (Scheme 4 and Figures S4–S6).

To prepare the bioconjugates of hapten **Ib**, we first functionalized proteins with propargyl groups at adequate molar ratios for their intended role and available lysine residues: 24.8 for BSA, 3.5 for OVA, and 1.3 for HRP (Scheme 4 and Figures S4–S6). The coupling reaction between hapten **Ib** and the alkyne-modified proteins *via* the CuAAC reaction was performed with 2–4 equiv of hapten per alkyne residue in 100 mM PB under nitrogen in the presence of the catalytic complex Cu(I)-tris(3-hydroxypropyltriazolylmethyl)amine (THPTA) and sodium ascorbate as a reducing agent. After 22 h at room temperature, the bioconjugates were concentrated and purified by size-exclusion chromatography. Hapten-to-protein loadings of 15.9, 2.3, and 0.4 were obtained for the bioconjugates BSA–**Ib**, OVA–**Ib**, and HRP–**Ib**, respectively, as determined by MALDI-TOF-MS (Scheme 4 and Figures S4–S6).

We first assessed the immune response in rabbits by immunization with bioconjugates BSA–**Ia** and BSA–**Ib**. When the two antisera obtained were evaluated by indirect competitive ELISA, inhibition curves with excellent IC_50_ values in the low nanomolar range (4.6–10.6 nM) were observed (Figure S7).

### Monoclonal Antibodies Recognize Derivatized Patulin (Adduct I) with Remarkable Affinity

The high affinity of the rabbit antisera for patulin after its transformation into adduct **I** motivated us to pursue the production of monoclonal antibodies (mAbs), given their high value in industry for the development of rapid commercial assays. To this end, standard hybridoma technology was employed with splenocytes from mice immunized with the bioconjugate BSA–**Ia**, the immunogen that elicited the best response in rabbits (Supporting Information). From two independent cell fusion experiments, three antibody-producing hybridomas capable of recognizing adduct **I** were generated. Following affinity purification, the mAbs were characterized by direct and indirect competitive ELISA using the homologous and the heterologous conjugate, *i.e.*, based on hapten **Ia** or **Ib**, respectively. As summarized in Table S2, the three antibodies successfully recognized both coating conjugates (OVA–**Ia** and OVA–**Ib**), yielding IC_50_ values for patulin below 7.0 nM. The most sensitive immunoreagent pair in this format was the combination of mAb #116 with heterologous conjugate OVA–**Ib** (IC_50_ = 2.9 nM). In the direct competitive format, even better IC_50_ values were found with mAbs #13 and #17 when used with the homologous enzyme tracer HRP–**Ia** (IC_50_ = 0.7–0.8 nM). Remarkably, the inhibition curves with these antibodies overlapped when either adduct **I** or patulin (after reacting with **7a**) were used as competitors, while they did not recognize **7a** or patulin when tested separately, nor did they recognize other regulated mycotoxins.

**Figure 1:**
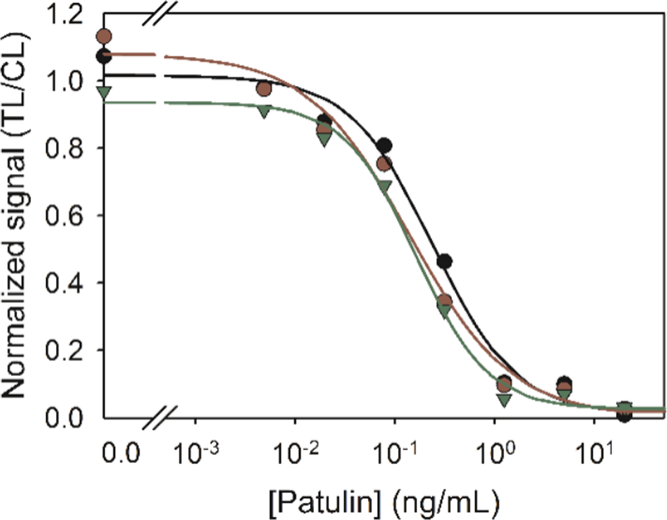
Representative inhibition curves by the immunochromatographic assay based on antibody #17 and conjugate BSA–**Ia**. Patulin standards were prepared in buffer (black curve) and in apple juice diluted with buffer 1/10 (red curve) and 1/50 (green curve). Following derivatization as described in the text, the standards were analyzed by the immunostrips. The color intensity at the test and control lines was recorded with a flatbed scanner, and TL/CL signals were normalized with respect to those in buffer.

To further evaluate the suitability of the generated antibodies and bioconjugates for the sensitive determination of patulin using rapid immunoanalytical methods, we also tested their performance in immunochromatographic strips. In these strips, the conjugate BSA–**Ia** was immobilized on the nitrocellulose membrane to form the test line, whereas an antimouse immunoglobulin antibody was applied 5 mm upstream to form the control line. The detection reagent consisted of antibodies coupled to gold nanoparticles precoated with goat antimouse immunoglobulins. As with other competitive assays, a fading signal at the test line indicates that the sample is positive for the presence of the analyte. Preliminary experiments showed that the lines were brighter and more defined with mAb #17, so further work utilized this antibody. Patulin standard solutions were prepared by serial dilution in assay buffer, and 2% (v/v) of a 1 mg/mL solution of the sodium salt of benzene-1,2-dithiol (**7a**) in water was added to each calibrator. After incubation at room temperature for 30 min, the samples were analyzed with the immunostrips, and the ratio between the signals at the test and control line (TL/CL) after 10 min was plotted against the patulin concentration on a semilogarithmic graph. As shown in Figure 1, the inhibition curve of the assay showed an excellent IC_50_ value of 0.2 ng/mL and a limit of quantitation (LOQ) of approximately 0.03 ng/mL, which are well below the maximum permitted levels of patulin in food.

**Figure 2:**
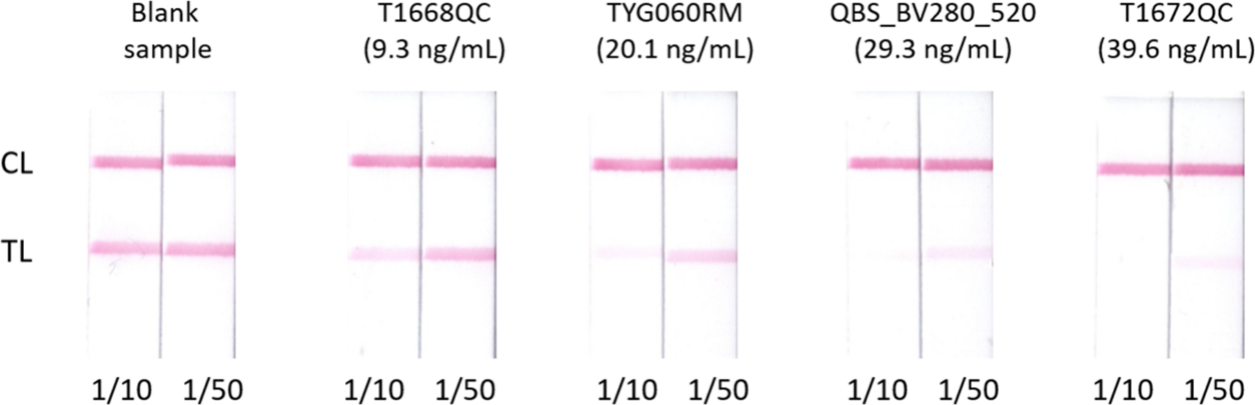
Visual aspect of scanned immunostrips after the analysis of apple juice samples contaminated with patulin. The blank sample (homemade juice from healthy apples) and quality control and reference materials were diluted 1/10 and 1/50 in assay buffer before analysis.

The assay was also tested in apple juice, which is the food product with the highest occurrence of patulin. First, matrix effects were evaluated by preparing patulin standard solutions in homemade fruit juice from healthy apples diluted 1/10 and 1/50 (v/v) in assay buffer. After dilution and derivatization, the calibrators were analyzed by the immunochromatographic assay as described before. The inhibition curves obtained from the diluted apple juice closely overlapped with the curve in buffer (Figure 1), indicating that the samples could be analyzed after simple dilution. To validate this claim, four apple juices naturally contaminated with patulin were analyzed with the developed immunostrips. These samples, which were commercial quality control and reference materials from proficiency testing, were diluted 1/10 and 1/50 in assay buffer, and the derivatization reagent was added. As shown in Table S3, the patulin concentrations determined by the immunostrips were consistent with the values reported by the suppliers for all four samples. Notably, this detection system also allows for easy visual identification of contaminated samples at regulated levels (Figure 2). For example, apple juices with patulin concentrations greater than 25 ng/mL resulted in barely visible signals at both 1/10 and 1/50 dilutions; the sample with less than 10 ng/mL showed a slight signal decrease only at 1/10 dilution; and the sample with patulin content between 10 and 25 ng/mL exhibited almost no signal at the test line at 1/10 dilution and a significant decrease at 1/50.

## Conclusions

We report here for the first time an efficient strategy for the development of immunoanalytical methods to determine mycotoxin patulin in food at regulated concentrations. The proposed approach leverages the high electrophilic reactivity of patulin and the previously unknown and remarkable reaction of patulin with aryl-1,2-dithiolates. Notably, this reaction proceeds very rapidly and quantitatively in an aqueous medium at room temperature, yielding a single compound in a clean manner. Moreover, the resulting adduct is stable and structurally complex enough to induce a strong immune response and antibody formation. As a result, three monoclonal antibodies with subnanomolar affinity for the patulin adduct were generated, and one of these antibodies was successfully incorporated into a prototype immunochromatographic assay for the rapid determination of patulin in apple juice samples, a long-standing target of food immunodiagnostic companies.

## Data Availability

The immunoreagents reported herein are available upon request for evaluation and research purposes. These developments have been patented (WO2021165557A1), and an exclusive license has been granted to Eurofins-Abraxis (Pennsylvania, USA; currently Gold Standard Diagnostics) for the production and commercialization of immunoanalytical methods for patulin.

## Supporting Information Available

Chemicals, apparatus, general experimental techniques, the preparation of adducts other than adduct **I**, the preparation of bioconjugates of adduct **I**, the MALDI-TOF-MS analysis of bioconjugates, the evaluation of the immune response in rabbits, and the production of monoclonal antibodies (PDF)
