# Supplementary material for: Harnessing the Intrinsic Chemical Reactivity of the Mycotoxin Patulin for Immunosensing
Source: Anal Chem. 2024 Jul 15;96(30):12370–7. doi: 10.1021/acs.analchem.4c01631 (PMC11295118; doi:10.1021/acs.analchem.4c01631)

SUPPORTING INFORMATION

Harnessing the intrinsic chemical reactivity of the mycotoxin patulin for immunosensing

Hadyn Duncan,[1, 2] Consuelo Agulló,[1] Josep V. Mercader,[2] Antonio Abad-Somovilla,[1,*] Antonio Abad-Fuentes[2,*]

[1] Department of Organic Chemistry, University of Valencia, Doctor Moliner 50, 46100 Burjassot, Valencia, Spain

[2] Institute of Agrochemistry and Food Technology (IATA), Spanish National Research Council (CSIC), Agustí Escardino 7, 46980 Paterna, Valencia, Spain

* Corresponding authors.

Email: antonio.abad@uv.es (A. Abad-Somovilla). Email: aabad@iata.csic.es (A. Abad-Fuentes)

Contents **Page**

1. General experimental procedures and techniques S-2
2. Thin layer chromatography (TLC) of the reaction between patulin and disodium benzene-1,2-thiolate S-4
3. ^{1}H NMR spectrum of the crude of the reaction between patulin and disodium benzene-1,2-thiolate S-5
4. NMR spectroscopic data of adduct **I** methyl ester S-6
5. Global minimum energy conformation of adduct **I** methyl ester S-7
6. Methylation of adduct **I** S-7
7. Reaction of patulin with aryl-1,2-dithiols. Formation of mono-dithiol adducts S-8
8. Preparation of hapten **Ia** dimethyl ester S-11
9. Preparation of the NHS ester of adduct **I** S-12
10. Preparation of O-(5-azidopentyl)hydroxylamine (**8**) S-13
11. Preparation of bioconjugates of adduct **I** S-13
12. MALDI mass spectrometry analysis of bioconjugates S-14
13. Evaluation of the immune response S-18
14. Monoclonal antibody generation, selection and purification S-19
15. Characterization of the monoclonal antibodies by competitive ELISA S-21
16. Immunochromatographic strips S-22
17. Determination of patulin in apple juice samples S-22
18. Copies of NMR spectra S-23

1. General experimental procedures and techniques

1.1. ***Reagents, equipment, and general techniques in synthesis procedures***

Tetrahydrofuran (THF) were distilled over Na and benzophenone under nitrogen atmosphere before use. CH_3CN were distilled from CaH_2 in the same way.[1] Anhydrous *N,N*-dimethylformamide (DMF) and MeOH were purchased from Fisher Scientific (Madrid, Spain). Patulin was purchased from Fermentek (Jerusalem, Israel). The remaining solvents and commercial reagents were used without prior purification. The operations with air and/or moisture-sensitive reagents were carried out under an inert atmosphere of dry nitrogen, using syringes or cannulas, oven-dried (140 °C) glass material, and freshly distilled and dried solvents. Reactions were monitored by thin-layer chromatography on precoated silica plates (0.25 mm layer thickness, Silica Gel 60 F_{254}) using UV light as the visualizing agent and ethanolic phosphomolybdic acid or aqueous ceric ammonium molybdate solutions and heat as developing agents. The synthesized compounds were purified by flash column chromatography using silica gel 60 (particle size 0.043–0.063 mm). IR spectra were recorded using a Nicolet Avatar 320 FT-IR spectrophotometer equipped with ATR (IR band intensities: w = weak, m = medium, s = strong). $^{1}H/^{13}C$ NMR spectra were recorded at 25 °C, in the solvent indicated, at 300/75 MHz (Bruker Avance DPX300 spectrometer), 400/101 MHz (Bruker AV400 spectrometer) or 500/125 MHz (Bruker Avance DRX500 spectrometer). The chemical shifts are expressed in ppm (δ scale) relative to the residual solvent as the internal reference in all cases [7.27/77.16 ppm, 2.05/29.84 ppm, 3.31/49.00 ppm and 4.79 ppm for the $^{1}H/^{13}C$ spectra in $CDCl_3$, acetone-d_6, methanol-d_4 and D_2O, respectively. Carbon substitution degrees were established by DEPT pulse sequences. Complete assignment of ^{1}H and ^{13}C chemical shifts of selected compound was made based on a combination of COSY, HSQC, HMBC and NOESY experiments. High-resolution mass spectra (HRMS) were obtained by electrospray ionization (ESI) mode in a premier Q-TOF mass spectrometer equipped with an electrospray source (Waters, Manchester, UK). The obtained data are expressed as mass/charge ratio (*m/z*).

1.2. Reagents and equipment used in antibody generation and immunoassays

BSA fraction V (cat. no. 10735094001) was purchased from Roche Applied Science (Mannheim, Germany). OVA grade VI (cat. no. A2512) was provided by Sigma/Aldrich (Madrid, Spain) and horseradish peroxidase (HRP, cat. no. 31490, activity: 307 U/mg) was from ThermoFisher Scientific (Madrid, Spain). Goat anti-mouse immunoglobulins were obtained from Jackson ImmunoResearch Laboratories Inc. (West Grove, PA, USA, cat. no. 115-005-008). Peroxidase-labeled rabbit anti-mouse immunoglobulins antibody conjugate was obtained from Dako (Glostrup, Denmark, cat. no. P026002-2) and peroxidase-labeled goat anti-rabbit immunoglobulins antibody conjugate from Bio-Rad Laboratories (Madrid, cat. no. 170-6515). Costar flat-bottom high-binding 96-well polystyrene ELISA plates were purchased from Corning (Corning, NY, USA, cat.

[1] Perrin, D. D; Armarego, W. L .F., in "Purification of Laboratory Chemicals", 4th ed.; Butterworth Heinemann Press: Oxford, 1996.

no. 3590). ELISA absorbance was determined employing a PowerWave HT from BioTek Instruments (Winooski, VT, USA). Microplate wells were washed with an ELx405 microplate washer also from BioTek Instruments. Nitrocellulose membranes (pore size: 15 µm) were obtained from MDI Membrane Technologies (Ambala, India, cat. no. 70CNPH-N-SS40) and backing cards were purchased from Kenosha (Amstelveen, The Netherlands). Cellulose sample pad (cat. no. CFSP173000) and absorbent pad were acquired from Merck-Millipore (Billerica, MA, USA) and Ahlstrom-Munksjö (Manchester, UK), respectively. 40-nm GAM-modified gold nanoparticles (OD = 10) were obtained from BBI solutions (Crumlin, UK, cat. no. BA.GAM40). A ZX1010 system from Biodot (West Sussex, UK) was employed for dispensing the immunoreagents onto the nitrocellulose membrane, immunostrips were manually assembled and cut using a CM5000 guillotine from Biodot. Immunostrips were scanned using an EPSON V39 scanner and RGB signal was processed using ImageJ (version 1.52a) free software.

Animals were supplied by Granja San Bernardo (Navarra). Female New Zealand rabbits [Crl:KBL(NWZ)] and female BALB/c mice (BALB/cAnNCrl) were kept and cared for by the Animal Production section from the Central Support Service for Experimental Research (SCSIE) (University of Valencia) for the entire immunization process. Animal manipulation was performed in compliance with the European Directive 2010/63/EU and the Spanish laws and guidelines (RD1201/2005 and 32/2007) concerning the protection of animals used for scientific purposes. Mouse plasmocytoma cell line P3-X63-Ag8.653 from the European Collection of Animal Cell Cultures (ECACC, Salisbury, United Kingdom) was purchased from Sigma. Cells were cultured at 37 °C under 90% humidity and 5% CO_2.

Complete and incomplete Freund adjuvants, Dulbecco modified Eagle medium (DMEM, high glucose content, cat. no. D6546), fetal bovine serum (FBS, cat. no. F7524), L-alanine-L-glutamine solution (L-Ala-L-Gln, 200 mM), non-essential amino acids (NEAA, 100×), Hybri-Max erythrocytes lysis buffer and polyethylene glycol (PEG 1500, cat. no. P7181) were obtained from Sigma-Aldrich. Gentamicin (50 mg/mL, cat. no. G1397), HT supplement (hypoxanthine 5 mM, thymidine 0.8 mM, cat. no. 41065-012) and HAT supplement (HT supplemented with aminopterin 20 µM, cat. no. 21060-017) were purchased from Gibco BRL (Paisley, United Kingdom). Hybridoma fusion and cloning supplement (HFCS, 50×, Roche, cat. no. 11363735001) and DMSO Hybri-Max (cat. no. D2650) were provided by Sigma.

2. Thin layer chromatography (TLC) of the reaction between patulin and disodium benzene-1,2-thiolate

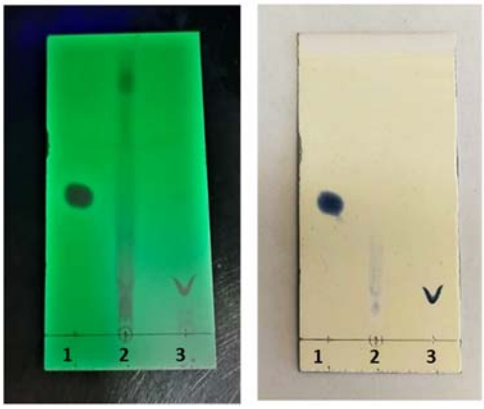

Figure S1. Thin layer chromatography (TLC) of the reaction between patulin and disodium benzene-1,2-thiolate. TLC plates show spots of (1) patulin, (2) disodium benzene-1,2-thiolate and (3) reaction mixture of patulin and disodium benzene-1,2-thiolate in 50 mM sodium phosphate buffer after 10 min. *Eluent*: 95:5 $CHCl_3$-isopropanol with 0.5% of HCO_2H. *Developing reagent:* UV light (left) and aqueous ceric ammonium molybdate solution and heat (right).

3. ^{1}H NMR spectrum of the crude of the reaction between patulin and disodium benzene-1,2-thiolate [Adduct I]

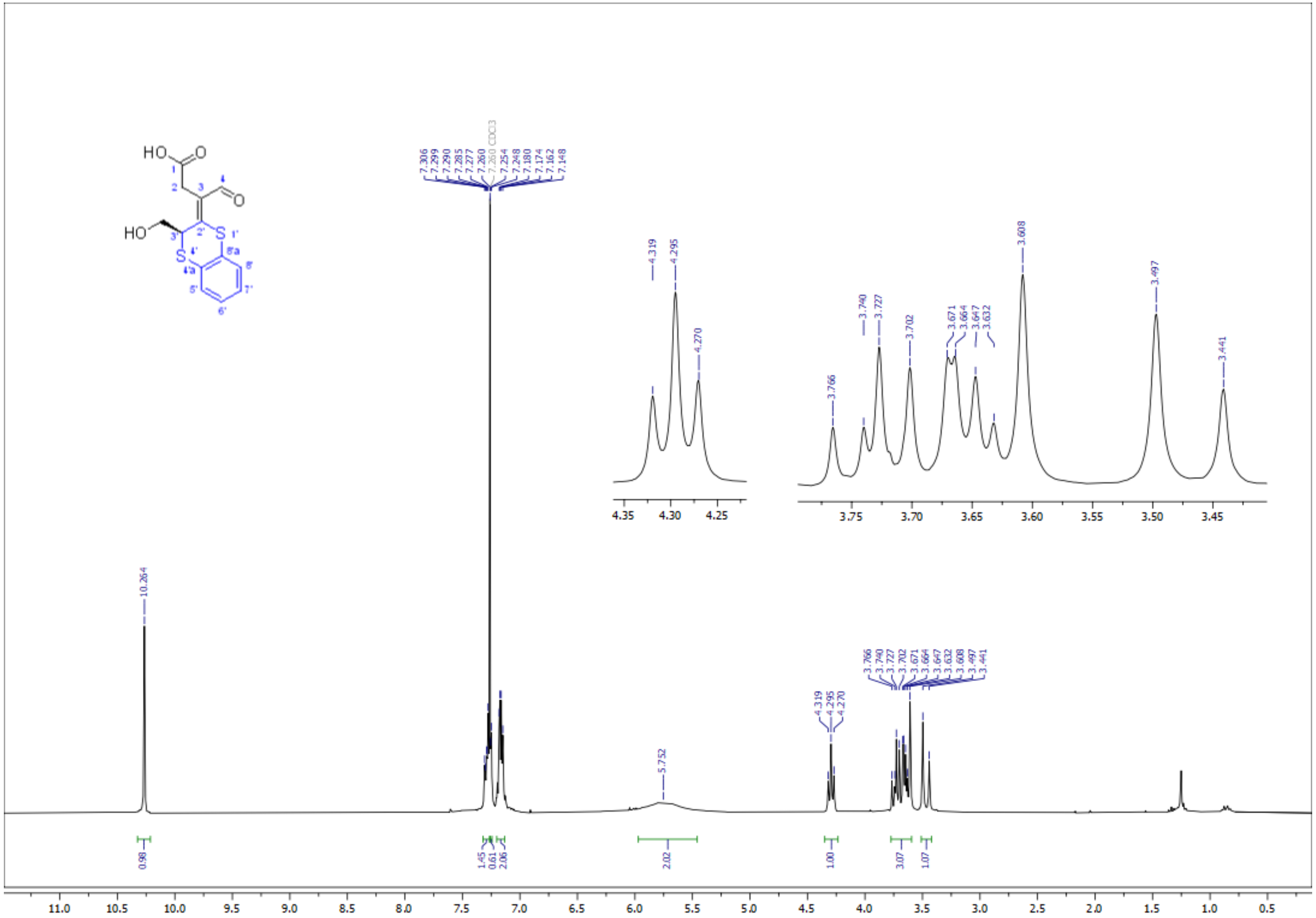

Figure S2. ^{1}H NMR spectrum (in $CDCl_3$) of the crude of the reaction between patulin and disodium benzene-1,2-thiolate in 50 mM PB (Adduct **I**)

4. NMR spectroscopic data of adduct I methyl ester

Table S1. NMR spectral data of adduct **I** methyl ester[$CDCl_3$, 400 (^1H)/101 (^{13}C) MHz].

Position assignment		Chemical shift (ppm)[a]		^1H multiplicity[b]	^1H–^{13}C connectivity at 2 bonds[c]	^1H–^{13}C connectivity at 3 or more bonds[c]	Observed NOE effects[d]
		^1H	^{13}C				
C-1	CO_2	–-	171.2	*n/a*	–	–	–
C-2	CH_2	3.62	32.0	AB system, J = 16.3 Hz	C-1, C-3	C-2', C-4	H-3'
		3.49			C-1, C-3	C-2', C-4, OCH_3	H-3'
C-3	C	–	126.4	*n/a*	–	–	–
C-4	CHO	10.31	188.0	s	C-3		
C-2'	C	–-	154.0	*n/a*	–	–	–
C-3'	CH	4.30	43.3	t, J = 7.4 Hz	C-2', CH_2OH	C-4'a	H-2/H'-2, OCH_3
C-4'a	C	–	126.8	*n/a*			
C-5'	CH	7.32-7.27	127.3	Part AA'	C-6'	C-7'	
C-8'	CH		130.6		C-7'	C-6'	
C-6' *	CH	7.22-7.14	127.18	----- (AA'BB' system) ---		C-4'a	
C-7' *	CH		127.16	Part BB'			
C-8'a	C	–	129.5	*n/a*	–	–	–
CH-OH		3.78-3.72	62.4	m			
CH'-OH		3.66		ddd, J = 12.8, 7.4, 4.4 Hz			
OCH_3		3.71	52.7	s	C-1		H-3', OH
OH		2.35	–	dd, J = 7.9, 5.6 Hz	CH_2OH		OCH_3

n/a: not applicable; s: singlet; s; dd: double doublet; ddd: double doublet of doublets, t: triplet; m: multiplet.

[a] Chemical shift assigned from the experiments of ^1H, ^{13}C, HSQC and HMBC in $CDCl_3$ as solvent.

[b] ^1H multiplicity and carbon substitution degrees obtained from the ^1H and edited-HSQC experiment.

[c] Connectivity between ^1H and ^{13}C positions deduced from HMBC experiments.

[d] Observed NOE effects in the 2D NOESY experiment.

* Interchangeable assignment.

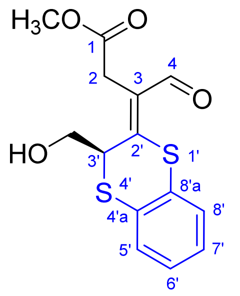

Adduct **I** methyl ester

5. Global minimum energy conformation of adduct I methyl ester

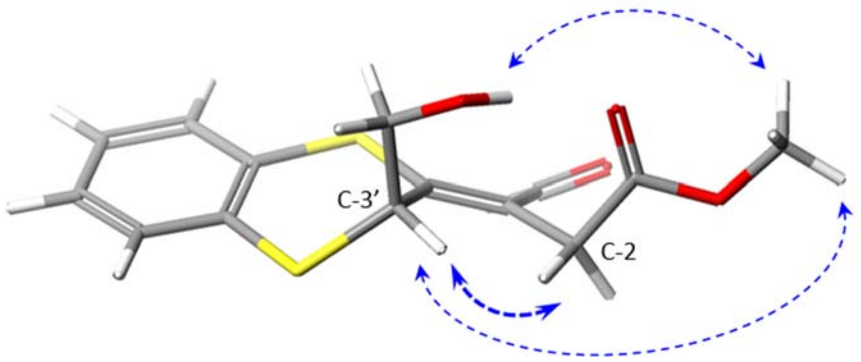

Figure S3. Global minimum energy conformation of adduct **I** methyl ester. Calculations were performed using Molecular Mechanics (MM3) as implemented in the SCIGRESS program (version 3.0.0). A systematic conformational search was performed (all rotatable bonds were rotated by 15 degree steps) and the geometry of the generated conformers was refined by performing an optimize geometry calculation in MOPAC using PM3 parameters and including solvation effects of water simulated by COSMO [MO-G-PM3_H2O]. Dotted arrows denote relevant NOESY correlations.

6. *Methylation of adduct* I. *Preparation of methyl (Z)-3-(3-(hydroxymethyl)benzo[b][1,4]dithiin-2(3H)-ylidene)-4-oxobutanoate* (Adduct I methyl ester)

A solution of adduct **I** (6.9 mg, 0.023 mmol) in THF (500 µL) was added to the outside tube of an Aldrich MNNG diazomethane generation apparatus. A solution of diazald® (53.1 mg, 0.248 mmol, 10.8 equiv) in diethylene glycol monoethyl ether (800 µL) was then added to the inside tube and the system assembly was completed. After cooling the outer tube in an ice bath, 800 µL of an aqueous solution of KOH (51.0 mg, 0.909 mmol, 39.5 equiv) was added dropwise to allow diazomethane generation. Upon completion, the reaction mixture was concentrated in vacuo and purified by silica gel flash chromatography, using $CHCl_3$ as eluent, to afford the methyl ester of adduct **I** (4.1 mg, 60.5%) as a yellow oil. ^1H NMR (400 MHz, $CDCl_3$) δ (ppm) 10.31 (s, 1H, HCO), 7.32-7.27 (part AA' of an AA'BB' system, 2H, H-5' and H-8'), 7.22-7.14 (part BB' of an AA'BB' system, 2H, H-6' and H-7'), 4.30 (t, *J* = 7.4 Hz, 1H, H-3'), 3.78-3.72 (m, 1H, *CH*OH), 3.66 (ddd, *J* = 12.8, 7.4, 4.4 Hz, 1H, *CH'*OH), 3.62 and 3.49 (AB system, *J* = 16.3 Hz, each 1H, H_2-2), 2.35 (dd, *J* = 7.9, 5.6 Hz, 1H, OH); ^{13}C NMR (126 MHz, acetone-d_6) δ (ppm) 188.7 (CH, C-4), 171.5 (C-1), 154.,0 (C-2'), 131.2 (CH, C-8'), 130.7 (C, C-8'a), 128.6 (C, C-4'a), 127.9 (CH, C-6'), 127.7 (CH, C-7'), 127.6 (CH, C-5'), 127.6 (C, C-3), 62.9 (CH_2OH), 43.7 (CH, C-3'), 32.4 (CH_2, C-2). See also Table S1 for a more detailed NMR spectroscopic data of this compound.

7. Reaction of patulin with aryl-1,2-dithiols

7.1. *Reaction of patulin with 4-methylbenzene-1,2-dithiol: Preparation of adduct* II

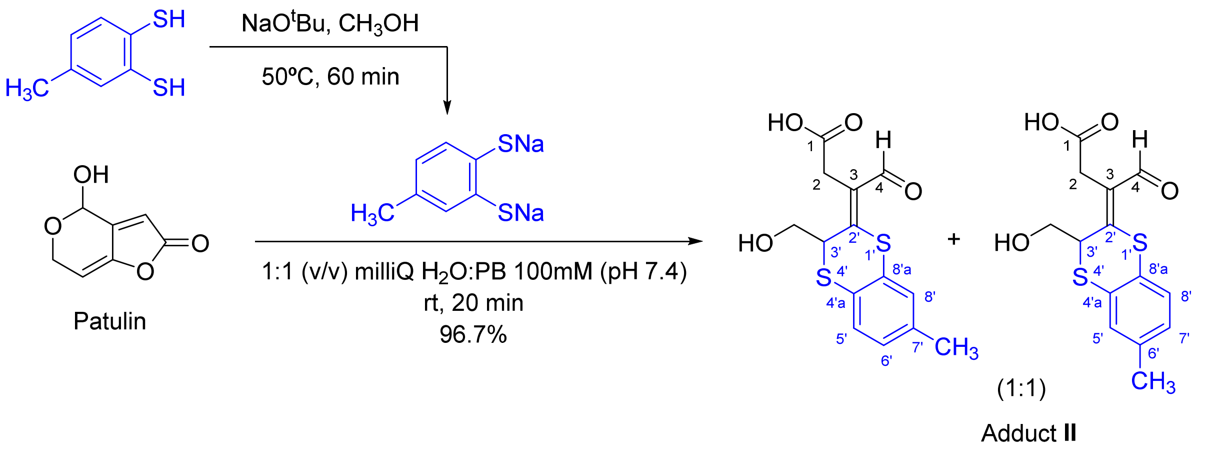

i) *Preparation of disodium 4-methylbenzene-1,2-thiolate*. 4-methylbenzene-1,2-dithiol (166 mg, 1.06 mmol) was added to a solution of sodium *tert*-butoxide (325 mg, 3.38 mmol, 2.4 equiv) in anhydrous methanol (1.5 mL) under nitrogen and the mixture was stirred at 50 °C for 60 min. The resulting mixture was cooled down to rt, concentrated at reduced pressure and the obtained solid residue washed with anhydrous THF (2×) and Et_2O (2×). The resulting white solid was dried under high vacuum overnight to give disodium 4-methylbenzene-1,2-thiolate (85.9 mg, 46%) as a white solid that was used directly without further purification. ^{1}H NMR (300 MHz, D_2O) δ (ppm) 7.30 (d, *J*= 8.1 Hz, 1H, H-6), 7.28 (s, 1H, H-3), 6.53 (dd, *J*= 7.8, 1.5 Hz, 1H, H-5), 2.11 (s, 3H, CH_3).

ii) *Reaction of patulin with disodium 4-methylbenzene-1,2-thiolate*

A solution of the above obtained (*bis*)thiolate (22.1 mg, 0.110 mmol, 1.1 equiv) in Milli-Q water (12 mL) was added into a stirred solution of patulin (15.6 mg, 0.101 mmol) in PB (12 mL). After 20 min of stirring at rt, the resulting yellowish mixture was cooled in an ice-water bath, acidified with HCO_2H to pH 3-4 and extracted with $CHCl_3$ (20 mL × 3). The combined organic phases were washed with brine (15 mL), dried over anhydrous $MgSO_4$ and concentrated under reduced pressure to give adduct **II** (30.3 mg, 96.6%) as a yellow oil, whose ^{1}H NMR spectrum showed that it was a practically equimolecular mixture of two chromatographically homogeneous regioisomers. IR ν_{max} (cm^{-1}) 3406m, 2922m, 1709s, 1655s, 1562m, 1469m, 1175s, 1044s; ^{1}H NMR (400 MHz, $CDCl_3$) δ (ppm) 10.27 (br s, 1H, H-4), 7.18 (d, *J* = 8.0 Hz, 0.5H, H-5’), 7.15 (d, *J* = 8.0 Hz, 0.5H, H-8’), 7.12 (br s, 0.5H, H-8’), 7.09 (br s, 0.5H, H-5’), 6.99 (dd, *J* = 8.1, 1.0 Hz, 0.5H, H-6’), 6.97 (dd, *J* = 8.1, 1.0 Hz, 0.5H, H-7’), 4.27 (m, 1H, H-3’), 3.75–3.58 (m, 2H, CH_2OH), 3.67 and 3.46 (each d, AB system, *J* = 16.6 Hz, 1H each, H-2), 2.31 and 2.30 (each s, 1.5H each, CH_3); ^{13}NMR (126 MHz, $CDCl_3$) δ (ppm) 188.3 (CH, C-4), 174.4 and 174.3 (C, C-1), 155.1 (C, C-2’), 137.6 and 126.0 (C, C-4’a), 137.4 and 129.1 (C, C-8’a), 131.1 and 127.5 (CH, C-8’), 130.5 and 127.0 (CH, C-5’), 128.4 and 128.3 (CH, C-7’/C-6’), 126.1 and 126.0 (C,

C-3), 125.9 and 123.3 (C, C-6'/C-7'), 62.3 and 62.2 (CH_2OH), 43.2 and 43.1 (CH, C-3'), 32.3 and 32.2 (CH_2, C-2), 21.13 and 21.07 (CH_3); HRMS *m/z* calcd for $C_{14}H_{13}O_3S_2$ $[M-H_2O+H]^{\cdot+}$ 293.0301, found $[M-H_2O+H]^{\cdot+}$ 293.0298.

7.2. *Reaction of patulin with 4,5-dimethylbenzene-1,2-dithiol: Preparation of adduct* III

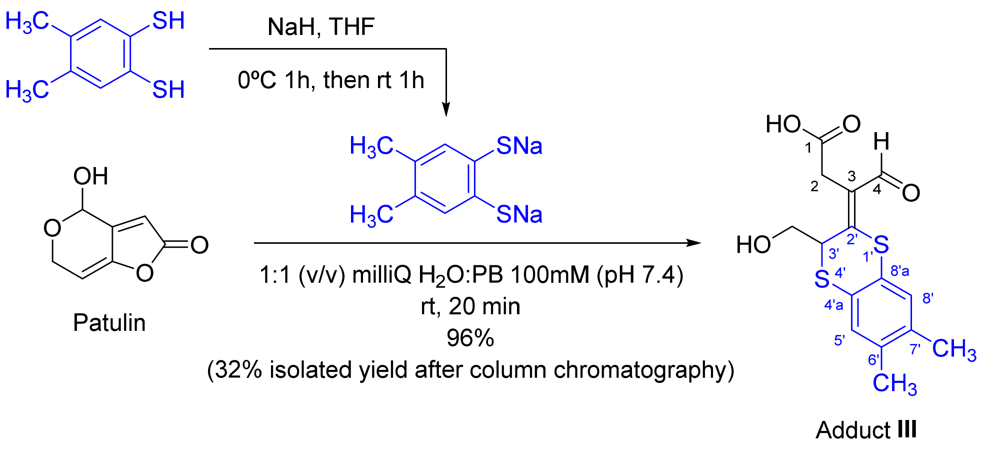

i) *Preparation of disodium 4,5-dimethylbenzene-1,2-thiolate*. Sodium hydride 60 wt % dispersion in mineral oil (6.2 mg, 3.7 mg of NaH, 0.154 mmol, 2.2 equiv) was weighed into a round bottom flask fitted with a magnetic stir-bar and purged with nitrogen. The NaH-oil dispersion was then washed with several portions of dry pentane and back-flushed with N_2, to give a dry white powder. The sodium hydride was then suspended in 150 µL of dry THF and cooled in an ice bath. A solution of 4,5-dimethylbenzene-1,2-dithiol[2,3] (12 mg, 0.071 mmol) in 300 µL of dry THF was then added dropwise (hydrogen evolution) and the mixture stirred for 1 h at 0 °C and for a further hour at rt. The resulting white suspension was transferred to a centrifuge tube and centrifuged at high speed. The supernatant was removed and the solid was washed with 500 µL of dry THF and centrifuged again. This procedure was repeated for two more times. The supernatant and washing were concentrated to dryness in the rotary evaporator and then placed under high vacuum to give disodium 4,5-dimethylbenzene-1,2-thiolate as a white powder (15 mg, 99%).

ii) *Reaction of patulin with disodium 4,5-dimethylbenzene-1,2-thiolate*. Adduct **III** was prepared as described for adduct **II**, using solutions of patulin (9.8 mg, 0.063 mmol) in PB (8.7 mL) and disodium 4,5-

---

[2] 4,5-Dimethylbenzene-1,2-dithiol was prepared from 1,2-dibromo-4,5-dimethylbenzene in two steps by an adaptation of the procedure described by Gleiter and Uschmann for the preparation of aryl-1,2-dithiols (**i**. $PhCH_2SH$, Cu_2O, Py, 130 °C, 17 days; **ii**. BBr_3, PhF, 70 °C, 17h. (a) Gleiter, R., Uschmann, J. Electronic structure of heterospirenes. PE spectroscopic investigations. *J. Org. Chem*. **51**, 370–380 (1986). (b) Schlindwein, S. H., Bader, K., Sibold, C., Frey, W., Neugebauer, P., Orlita, M., van Slageren, J., Gudat, D. New Selective Synthesis of Dithiaboroles as a Viable Pathway to Functionalized Benzenedithiolenes and Their Complexes. *Inorg. Chem.* **55**, 6186–6194 (2016). (c) Altamura, M., Fedi, V., Giannotti, D., Paoli, P., Rossi, P. Privileged structures: Synthesis and structural investigations on tricyclic sulfonamides. *New J. Chem.* **33**, 2219–2231 (2009).

[3] NMR data of 4,5-dimethylbenzene-1,2-dithiol: ^1H NMR (300 MHz, $CDCl_3$) δ (ppm) 7.16 (s, 2H, H-3 and H-6), 3.63 (s, 2H, 2xSH), 2.17 (2, 6H, $2xCH_3$).

dimethylbenzene-1,2-thiolate (15 mg, 0.070 mmol, 1.1 equiv) in Milli-Q water (8.7 mL). Crude adduct **III** (19.7 mg, 96.6%), also obtained as a yellow oil, showed by ^{1}H NMR a purity similar to those of the previously prepared adducts. As with the rest of above prepared adducts, adduct **III** could be chromatographied on silica gel, albeit at the cost of a significant reduction in the yield of the purified product. Thus, chromatographic purification of the above crude product, using 95:5 $CHCl_3$-MeOH as eluent, afforded adduct **III** in only 32% yield (6.5 mg). IR ν_{max} (cm^{-1}) 3375m, 2922m, 1716m, 1651s, 1558m, 1455m; ^{1}H NMR (300 MHz, acetone-d_6) δ (ppm) 10.23 (s, 1H, HCO), 7.14 (s, 1H, H-8'), 7.11 (s, 1H, H-5'), 4.47 (dd, *J* = 7.9, 6.8 Hz, 1H, H-3'), 3.69 (dd, *J* = 11.1, 7.9 Hz, 1H, *CH*OH), 3.66 and 3.58 (each d, AB system, *J* = 16.9 Hz, 1H each, H-2), 3.60 (dd, *J* = 11.1, 6.8 Hz, 1H, *CH'*OH), 2.82 (br s, 2H, 2xOH), 2.23 and 2.22 (each s, 3H each, 2xCH_3); ^{13}13 NMR (75 MHz, acetone-d_6) δ (ppm) 188.5 (CH, C-4), 171.7 (C, C-1), 154.9 (C, C-2'), 136.9 (C, C-4'a), 136.8 (C, C-8'a), 131.8 (CH, C-8'), 128.2 (CH, C-5'), 127.3 (C, C-6'), 127.3 (C, C-7'), 125.0 (C, C-3), 62.9 (CH_2OH), 43.9 (CH, C-3'), 32.4 (CH_2, C-2), 19.2 (2xCH_3); HRMS *m/z* calcd for $C_{15}H_{17}O_4S_2$ $[M+H]^{·+}$ 325.0563, found $[M+H]^{·+}$ 325.0558.

7.3. *Reaction of patulin with 4,5-dimercaptophthalonitrile: Preparation of adduct* IV

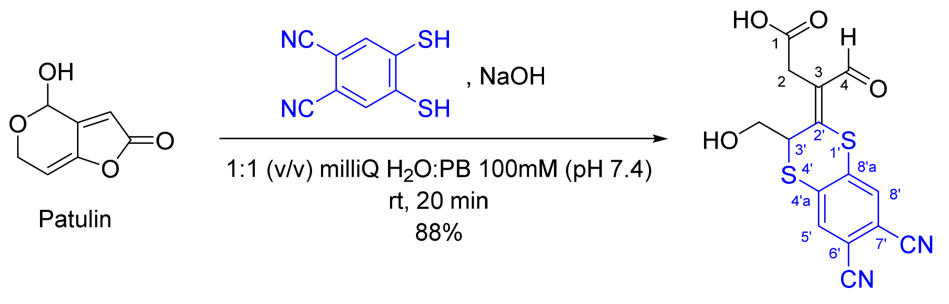

A solution of 4,5-dimercaptophthalonitrile[4,5] (8.8 mg, 0.046 mmol, 1.05 equiv) in 16 mM aqueous NaOH (6.2 mL, 4.0 mg, 0.099 mmol, 2.2 equiv) was added to a stirred solution of patulin (7.0 mg, 0.045 mmol) in PB (6.2 mL) and the mixture was stirred for 20 min at rt. Work up as described for the preparation of adduct **II** afforded adduct **IV** (17.4 mg, 88%) as a dark yellow oil. IR ν_{max} (cm^{-1}) 3442m, 2232s, 1716s, 1668m, 1571s, 1518m, 1470m, 1220s, 1118s; ^{1}H NMR (300 MHz, acetone-d_6) δ (ppm) 10.19 (s, 1H, HCO), 8.08 (s, 1H, H-8'), 8.01 (s, 1H, H-5'), 4.77 (t, *J* = 7.3 Hz, 1H, H-3'), 3.76 (dd, *J* = 11.3, 7.8 Hz, 1H, *CH*OH), 3.75 and 3.68 (each d, AB system, *J* = 17.0 Hz, 1H each, H-2), 3.68 (dd, *J* = 11.3, 6.8 Hz, 1H, *CH'*OH); ^{13}NMR (75 MHz, acetone-d_6) δ (ppm) 189.5 (CH, C-4), 171.2 (C, C-1), 148.6 (C, C-2'), 137.9 (C, C-4'a), 136.5 (C, C-8'a), 135.2 (CH, C-5'), 132.3 (CH, C-

---

[4] 4,5-Dimercaptophthalonitrile was prepared from 4,5-dichlorophthalonitrile in two steps as described by Simao *et al.* (**i**. $PhCH_2SH$, DMF, rt, 20h; **ii**. $AlCl_3$, bencene, rt, 1.5h). Simao, D., Alves, H., Belo, D., Rabaca, S., Lopes, E. B., Santos, I. C., Gama, V., Duarte, M. T., Henriques, R. T., Novais , H., Almeida, M. Synthesis, structure and physical properties of tetrabutylammonium salts of nickel complexes with the new ligand dcbdt = 4,5-dicyanobenzene-1,2-dithiolate, $[Ni(dcbdt)_2]^{z-}$ (z = 0.4, 1, 2). *Eur. J. Inorg. Chem.* 3119–3126 (2001).

[5] NMR data of 4,5-dimercaptophthalonitrile: ^{1}H NMR (300 MHz, acetone-d_6) δ (ppm) 8.06 (s, 2H, H-3 and H-6); ^{13}C RMN (75 MHz*,* acetone-d_6) δ (ppm) 140.6 (2C, C-1 and C-2), 134.2 (2C, C-3 and C-6), 116.0 (2C, 2xCN), 112.7 (2C, C-4 and C-5).

8’), 129.5 (C, C-3), 115.9 (2C, 2xCN), 113.0 (C, C-6’), 112.8 (C, C-7’), 63.0 (CH_2OH), 43.2 (CH, C-3’), 32.9 (CH_2, C-2); HRMS *m/z* calcd for $C_{15}H_9N_2O_4S_2$ [M-H]$^{\cdot-}$ 345.0009, found [M-H]$^{\cdot-}$ 344.9994.

7.4. *Reaction of patulin with ethane-1,2-dithiol. Preparation of adduct* S1

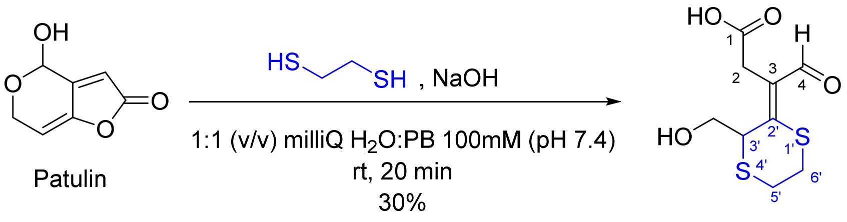

A solution of ethane-1,2-dithiol (5.4 µL, 6.05 mg, 0.064 mmol, 1.05 equiv) in 12 mM aqueous NaOH (12.0 mL, 5.7 mg, 0.143 mmol, 2.3 equiv) was added to a stirred solution of patulin (9.6 mg, 0.062 mmol) in PB (12.0 mL) and the mixture was stirred at rt for 20 min. The reaction mixture was cooled in an ice-water bath, acidified with formic acid to pH 3 and extracted with $CHCl_3$ (20 mL x 3). The combined organic layers were washed with brine (15 mL), dried over anhydrous $MgSO_4$ and concentrated in vacuo to give an oily residue (18 mg). The addition of $CHCl_3$ (0.8 mL) resulted in the appearance of a white solid that was separated by centrifugation, washed with acetone and dried under reduced pressure. It was identified as the adduct **S1** (4.7 mg, 30%). IR ν_{max} (cm^{-1}) 3356m, 2920s, 2853m, 2113m, 1701m, 1654s, 1407s, 1196s; 1092s, 1047s, 967s; ^{1}H NMR (300 MHz, methanol-d_4) δ (ppm) 10.42 (s, 1H, *H*CO), 4.32 (dd, *J* = 11.2, 8.7 Hz, 1H, *CH*OH), 4.04 (dd, *J* = 11.2, 6.1 Hz, 1H, *CH’*OH), 3.78 (dd, *J* = 8.7, 6.1 Hz, 1H, H-3’), 3.55 and 3.36 (each d, AB system, *J* = 16.8 Hz, 1H each, H-2), 3.42 and 3.40 (each dd, *J* = 10.5, 1.1 Hz, 1H each, H-6’) 2.97 and 2.63 (each ddd, *J* = 9.9, 4.7, 1.4 Hz, 1H each, H-5’); ^{13}C NMR (300 MHz, acetone-d_6) δ (ppm) 190.9 (C-4), 170.0 (C-1), 148.1 (C-2’), 130.7 (C-3), 65.2 (CH_2OH), 43.4 (C-3’), 34.6 (C-2), 33.3 (C-6’), 33.1 (C-5’); HRMS calcd for $C_9H_{13}O_4S_2$ [M+H]$^{\cdot+}$ 249.0250, found [M+H]$^{\cdot+}$ 249.0245.

8. Preparation of methyl (3*Z*,4*E*)-3-(3-(hydroxymethyl)benzo[b][1,4]dithiin-2(3H)-ylidene)-4-((2-methoxy-2-oxoethoxy)imino)butanoate (Hapten Ia dimethyl ester)

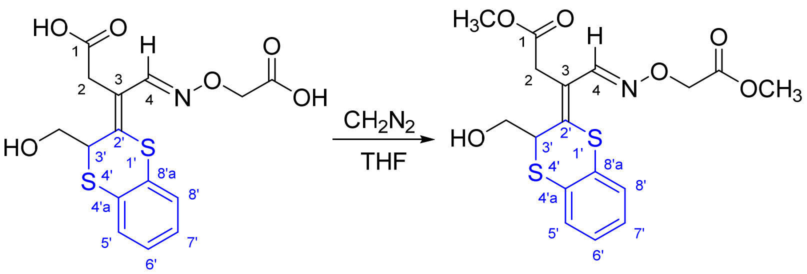

Hapten **Ia** (5.5 mg, 0.0149) was methylated with diazomethane as described above for adduct **I**. Upon completion, the reaction mixture was concentrated in vacuo and the residue chromatographed on silica

gel, using a 95:5 mixture of $CHCl_3$-MeOH as eluent, to afford the dimethyl ester of hapten **Ia** (4 mg, 67.6%) as a yellowish oil. ^{1}H NMR (500 MHz, $CDCl_3$) δ (ppm) 8.60 (s, 1H, H-4), 7.26 (dd, *J* = 7.6, 1.8 Hz, 1H, H-8’), 7.20 (dd, *J* = 7.7, 1.7 Hz, 1H, H-5’), 7.14 (td, *J* = 7.6, 1.8 Hz, 1H, H-6’), 7.10 (td, *J* = 7.4, 1.8 Hz, 1H, H-7’), 4.66 (s, 2H, $NOCH_2$), 4.27 (t, *J* = 7.4 Hz, 1H, H-3’), 3.78 (s, 3H, $OCH_2CO_2\mathit{CH_3}$), 3.72 (m, 1H, *CH*OH), 3.69 (s, 3H, $CO_2\mathit{CH_3}$), 3.62 (m, 1H, *CH’*OH), 3.61 and 3.55 (each d, AB system, *J* = 16.5 Hz, 1H each, H-2), 2.35 (t, *J* = 6.9 Hz, 1H, HO); ^{13}C NMR (126 MHz, $CDCl_3$) δ (ppm) 171.3 (C, C-1), 170.2 (C, $\mathit{CO_2}CH_3$), 148.2 (CH, C-4), 139.2 (C, C-2’), 130.4 (CH, C-8’), 130.3 (C, C-8’a), 127.1 (C, C-5’), 126.8 (CH, C-6’), 126.7 (CH, C-4’a), 126.5 (CH, C-7’), 120.8 (C, C-3), 71.2 ($NOCH_2$), 63.0 (CH_2OH), 52.5 (CH_3, $OCH_2CO_2\mathit{CH_3}$), 52.1 (CH_3, $CO_2\mathit{CH_3}$), 43.1 (CH, C-3’), 33.5 (CH_2, C-2); HRMS calcd for $C_{17}H_{20}NO_6S_2$ $[M+H]^{\cdot+}$ 398.0727, found $[M+H]^{\cdot+}$ 398.0740.

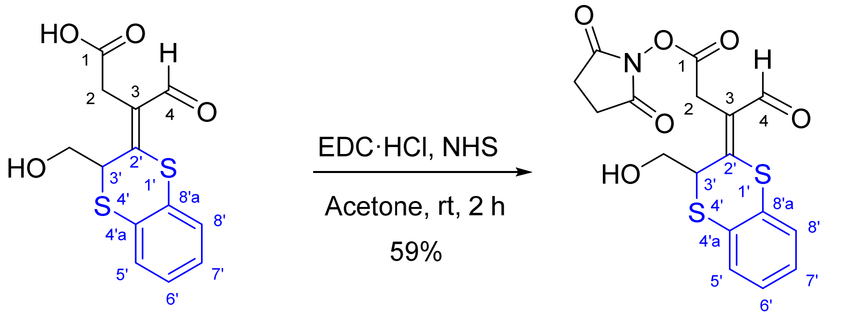

Adduct **I** NHS ester of adduct **I**

9. Preparation of 2,5-dioxopyrrolidin-1-yl (Z)-3-(3-(hydroxymethyl)benzo [b][1,4]dithiin-2(3H)-ylidene)-4-oxobutanoate (NHS ester of adduct I)

Adduct **I** (5.2 mg, 0.018 mmol), EDC·HCl (4.2 mg, 0.022 mmol, 1.25 equiv) and NHS (3.5 mg, 0.022 mmol, 1.25 equiv) were weighed into a reaction vial and purged with nitrogen. Then, dry acetone was added (400 µL) and the mixture was stirred for 2 h at rt. Upon completion, cold water (5 mL) was added to the vial and the mixture was extracted with $CHCl_3$ (3 × 10 mL). The combined organic phases were washed with brine (10 mL), dried over anhydrous $MgSO_4$ and concentrated in vacuo to give the NHS ester of adduct **I** (4.1 mg, 59%) as a yellowish oil. ^{1}H NMR (500 MHz, $CDCl_3$) δ (ppm) 10.33 (s, 1H, H-4), 7.35–7.28 (m, 2H, H-8’ and H-5’), 7.23–7.15 (m, 2H, H-6’ and H-7’), 4.20 (t, *J* = 7.3 Hz, 1H, H-3’), 4.06 and 3.73 (each d, AB system, *J* = 17.3 Hz, 1H each, H-2), 3.71 (dd, *J* = 11.7, 7.2 Hz, 1H, *CH*OH), 3.65 (dd, *J* = 11.7, 7.5 Hz, 1H *CH’*OH), 2.85 (wide s, 4H, $CO\mathit{CH_2CH_2}CO$).

10. Preparation of O-(5-azidopentyl)hydroxylamine (8)

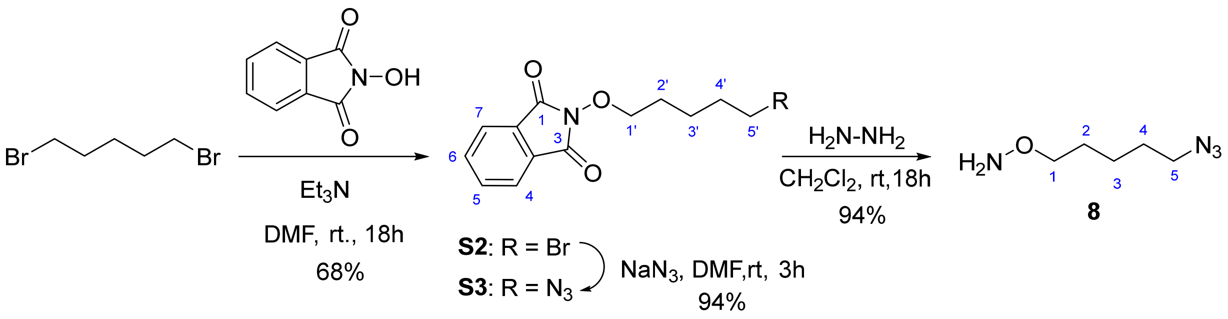

(i) 1,5-Dibromopentane (3.4 mL, 5.74 g, 25 mmol, 2 equiv) and Et_3N (3.8 mL, 27.3 mmol, 2.2 equiv) were added dropwise to a stirred solution of 2-hydroxyisoindoline-1,3-dione (2.0 g, 12.26 mmol) in anhydrous DMF (6 mL) under N_2 at rt. The reaction mixture was stirred for 6 h and then diluted in water and extracted with EtOAc. The combined organic phases were washed with brine, dried over anhydrous $MgSO_4$ and purified by silica gel flash chromatography, using Hexane/AcOEt (6:4) as eluent, to afford **S2** as a white solid (2.60 g, 68%). ^{1}H NMR (300 MHz, acetone-d_6) δ (ppm) 7.87 (m, 4H, H-4, H-5, H-6 and H-7), 4.23 (t, *J* = 6.2 Hz, 1H, H-1’), 3.55 (t, *J* = 6.8 Hz, 1H, H-5’), 1.96 (dt, *J* = 14.1, 7.0 Hz, 1H, H-4’), 1.80 (m, 2H, H-2’), 1.70 (m, 2H, H-3’).

(ii) A solution of the above obtained bromo-hydroxyisoindoline derivative **S2** (2.60 g, 8.33 mmol) and sodium azide (1.10 g, 16.92 mmol, 2 equiv) in anhydrous DMF (9 mL) was stirred at 40 °C under N_2 for 3 h, then diluted with water and extracted with EtOAc. The combined organic phases were washed with brine, dried over anhydrous $MgSO_4$ and concentrated in vacuo to afford azide-hydroxyisoindoline **S3** as a light yellow oil (2.15 mg, 94%). ^{1}H NMR (300 MHz, acetone-d_6) δ (ppm) 7.87 (m, 4H, H-4, H-5, H-6 and H-7), 4.23 (t, *J* = 6.3 Hz, 1H, H-1’), 3.39 (t, *J* = 6.6 Hz, 1H, H-5’), 1.80 (pent, *J* = 6.5 Hz, 1H, H-4’), 1.74-1.52 (m, 2H, H-2’ and H-3’).

(iii) Hydrazine monohydrate (286 µL, 5.84 mmol, 1.6 equiv) was added dropwise to the solution of azide-hydroxyisoindoline **S3** (1.00 g, 3.65 mmol) in CH_2Cl_2 (4 mL) and the mixture was stirred overnight at rt. The reaction mixture was diluted with CH_2Cl_2 and washed twice with brine, dried over anhydrous $MgSO_4$ and purified by silica gel flash chromatography, using a mixture of hexane-EtOAc (8:2) as eluent, to afford hydroxylamine-azide **8** as a white amorphous solid (495 mg, 94%). ^{1}H NMR (300 MHz, $CDCl_3$) δ (ppm) 3.66 (t, *J* = 6.4 Hz, 1H, H-1), 3.27 (t, *J* = 6.9 Hz, 1H, H-5), 1.60 (m, 4H, H-2 and H-4), 1.47 (m, 2H, H-3); ^{13}C NMR (75 MHz, $CDCl_3$) δ (ppm) 75.8 (CH_2, C-1), 51.5 (CH_2, C-5), 28.8 (CH_2, C-4), 28.0 (CH_2, C-2), 23.4 (CH_2, C-3); HRMS (ESI) *m/z* calcd for $C_5H_{13}N_4O$ $[M+H]^{\cdot+}$ 145.1084, found $[M+H]^{\cdot+}$ 145.1083.

11. *Preparation of bioconjugates of adduct I*

For the preparation of the bioconjugate BSA–adduct **I**, 132 µL of a 50 mM solution in DMF of the NHS ester of adduct **I** (6.6 µmol, 30 equiv) were added slowly with stirring to 1.0 mL of a solution of BSA (15 mg/mL, 0.22 µmol) in PB, and the conjugation reaction mixture was stirred overnight at rt. For the

preparation of the bioconjugate OVA–adduct **I,** 125 µL of a 50 mM solution in DMF of the NHS ester of adduct **I** (6.3 µmol, 20 equiv) were added to 1.85 mL of a 15 mg/mL solution of OVA (0.63 µmol) in PB, and the conjugation reaction mixture was stirred overnight at room temperature. The bioconjugates were purified by size-exclusion chromatography and sterilized by filtration before being stored at – 20 °C.

12. MALDI mass spectrometry analysis of bioconjugates

For sample preparation, 100 µL of bioconjugates (0.5–1 mg/mL) were dialyzed against milli-Q water and lyophilized. The samples were dissolved in Milli-Q water to a theoretical final concentration of 1 mg/mL, and 0.8 µL was spotted onto the MALDI plate. After the droplet was air-dried at rt, 0.8 µL of matrix (10 mg/mL sinapinic acid in 70% MeCN, 0.1% trifluoroacetic acid) was added and allowed to air-dry at rt. Then, samples were analyzed in a 5800 MALDI TOF/TOF (ABSciex) apparatus in positive linear mode (1500 shots every position) in a mass range of 12000–100000 *m/z*. Previously, the plate was calibrated with 1 µL of the TOF/TOF calibration mixture (ABSciex), in 13 positions. Every sample was calibrated by 'close external calibration' method with a BSA, OVA or HRP spectrum acquired in a close position. The analysis of the results was performed using the mMass program (http://www.mmass.org/).

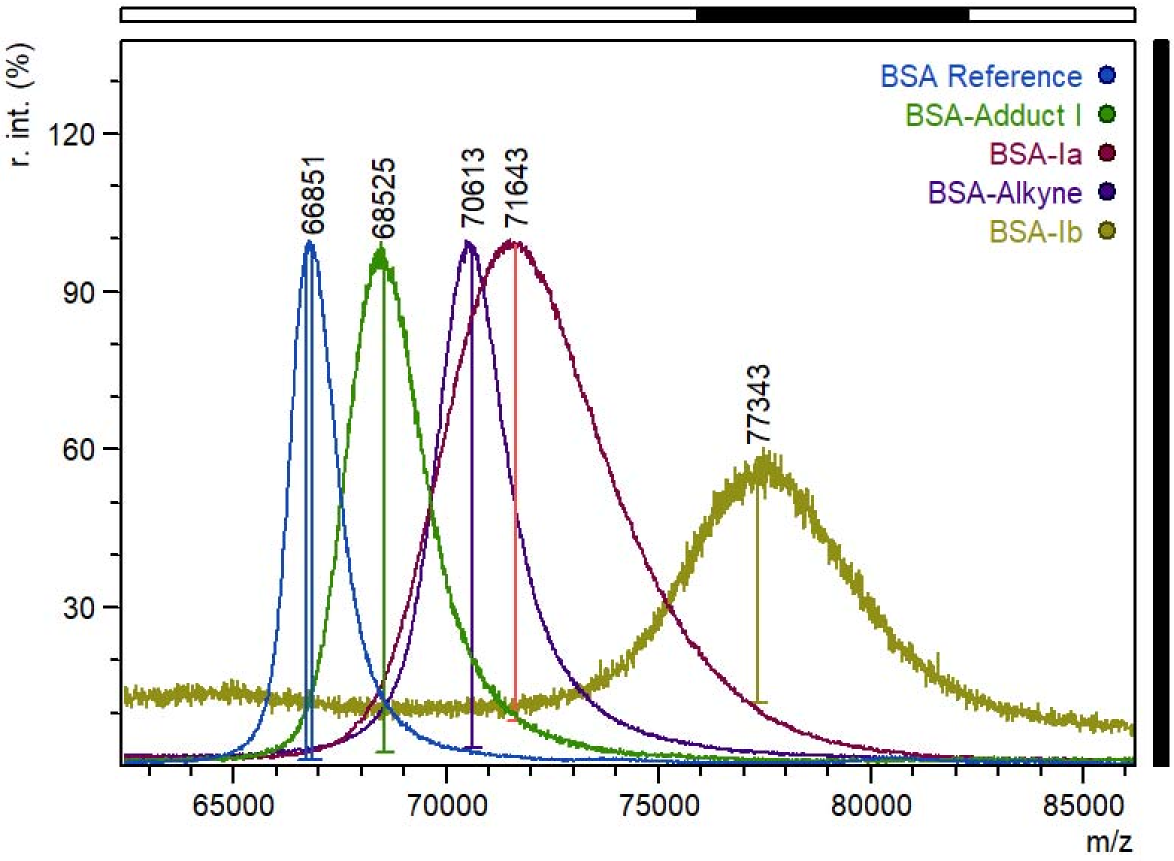

	m/z	Δm/hapten	Δ(*m/z*)	MR_o	MR
**BSA**	66851	–	–	–	–
**BSA-Adduct I**	68525	278	1674	60	6.0
**BSA-Ia**	71643	351	4792	60	13.7
**BSA-Alkyne**	70613	152	3762	40	24.8
**BSA-Ib**	77343	422	6730	50	15.9

Figure S4. MALDI-TOF mass spectra (singly charged ions) of BSA (blue), BSA modified with alkyne **9** (purple), and the corresponding conjugates with adduct **I** (green), hapten **Ia** (red), and hapten **Ib** (olive). Spectrum intensities are normalized. MR_o and MR: initial and final hapten-to-protein molar ratio, respectively.

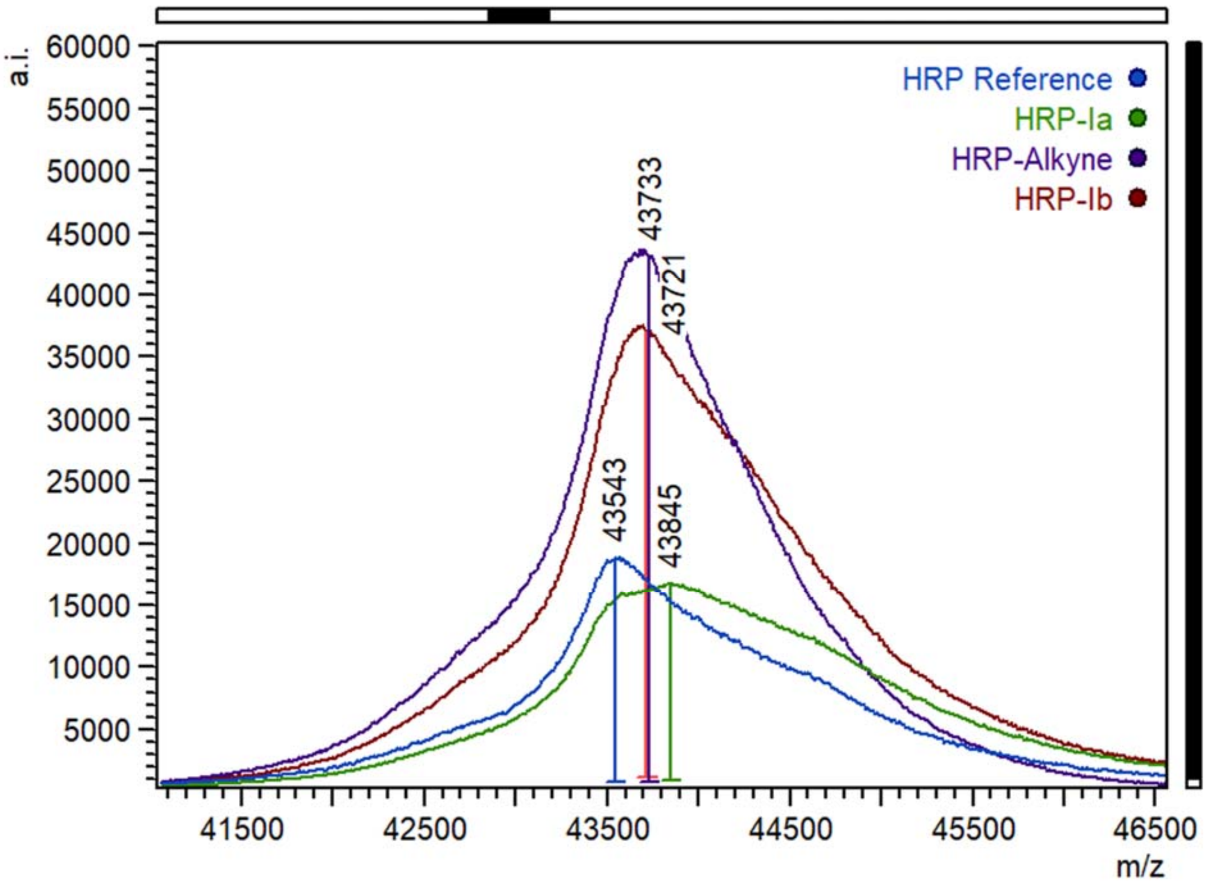

	m/z	Δm/hapten	Δ(*m/z*)	MR_o	MR
**HRP**	43543	–	–	–	–
**HRP-Ia**	43845	351	302	20	0.9
**HRP-Alkyne**	43733	152	190	10	1.3
**HRP-Ib**	43721	422	178	4	0.4

Figure S5. MALDI-TOF mass spectra (doubly charged ions) of OVA (blue), OVA modified with alkyne **9** (black), and the corresponding conjugates with adduct **I** (green), hapten **Ia** (red), and hapten **Ib** (olive). MR_o and MR: initial and final hapten-to-protein molar ratio, respectively.

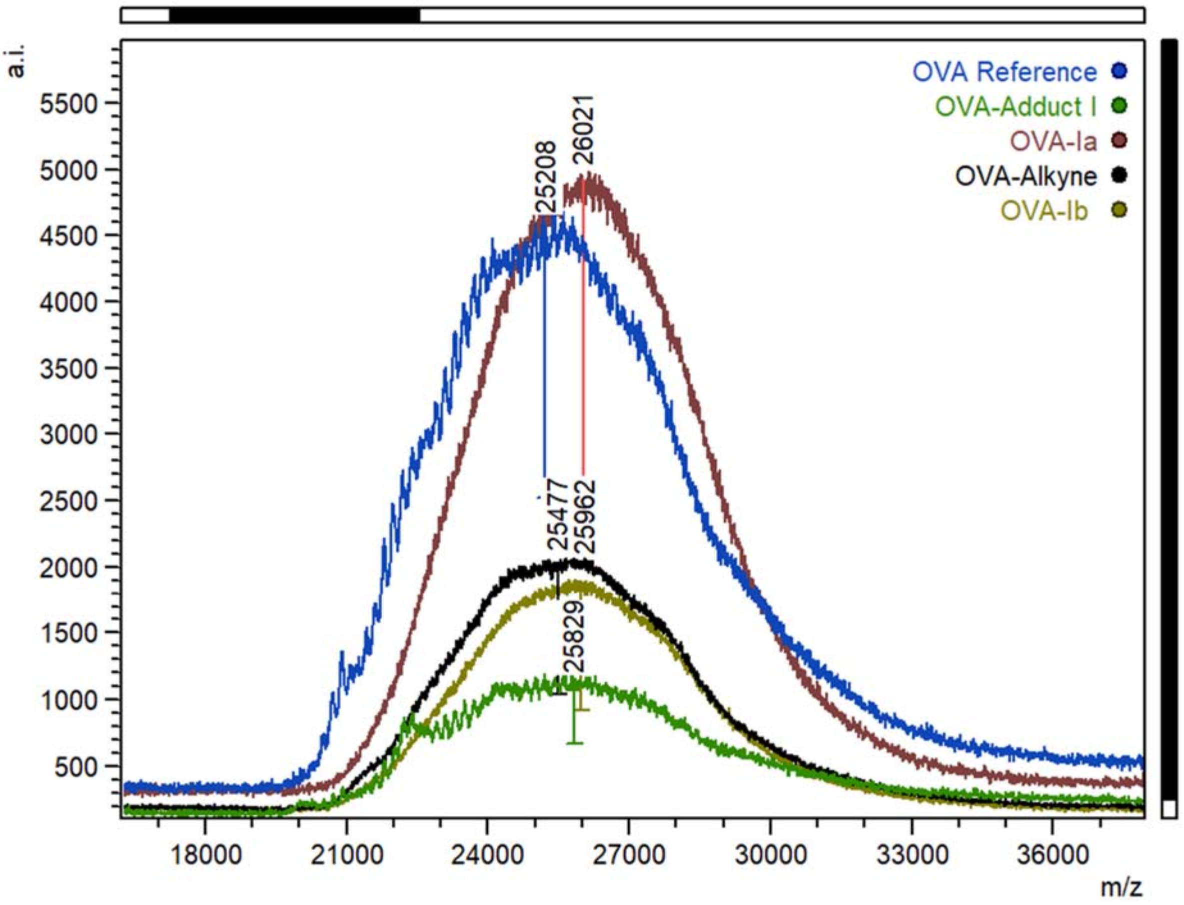

	m/z	Δm/hapten	Δ(*m/z*)	MR_o	MR
**OVA**	25208	–	–	–	–
**OVA-Adduct I**	25829	278	621	10	4.5
**OVA-Ia**	26021	351	813	35	4.6
**OVA-Alkyne**	25477	152	269	12	3.5
**OVA-Ib**	25962	422	485	15	2.3

Figure S6. MALDI-TOF mass spectra (singly charged ions) of HRP (blue), HRP modified with alkyne **9** (purple), and the corresponding conjugates with hapten **Ia** (green) and hapten **Ib** (red). MR_o and MR: initial and final hapten-to-protein molar ratio, respectively.

13. Evaluation of the immune response

Female New Zealand white rabbits were inoculated with 300 µg of bioconjugates BSA–adduct I, BSA–**Ia**, or BSA–**Ib**, in a 1 mL dose of a 1:1 emulsion between sterile PB and Freund's adjuvant (complete for the first dose and incomplete for subsequent doses). A total of 4 injections were subcutaneously administered in the dorsal area of the animals at 21-day intervals. Rabbits were exsanguinated 10 days after the final dose. Blood samples were allowed to coagulate overnight at 4 °C, and sera were separated by double centrifugation (3000 × g, 20 min). Antibodies were precipitated twice with ammonium sulphate and stored at 4 °C until evaluation by indirect competitive ELISA. To do so, 96-well microtiter plates were coated by adding 100 µL per well of OVA–**Ia** or OVA–**Ib** at 1 µg/mL in 50 mM carbonate buffer, pH 9.6. After overnight incubation at rt the plates were washed four times with a 150 mM NaCl solution. Eight patulin standard solutions were prepared by serial dilution in PBS, and 2% (v/v) of a 1 mg/mL of disodium benzene-1,2-dithiolate (**7a**) in water was added to each calibrator. The solutions were incubated at rt for 30 min to quantitatively convert patulin to adduct **I**. The competitive step was started by transferring to the microtitre plates 50 µL per well of the derivatized patulin standards plus 50 µL per well of antiserum diluted 1/3000 in PBS-T, and the plates were incubated 1 h at rt. After plate washing, 100 µL per well of the enzyme-labeled secondary antibody (GAR–HRP at 1/10000 in PBS-T containing 10% (v/v) adult bovine serum) was added and incubated at rt for an additional hour. The plates were washed again and the signal was generated by adding 100 µL per well of the enzyme substrate solution. Following incubation during 10 min at rt, the reaction was stopped by adding 100 µL per well of 1 M H_2SO_4, and the plates were immediately read.

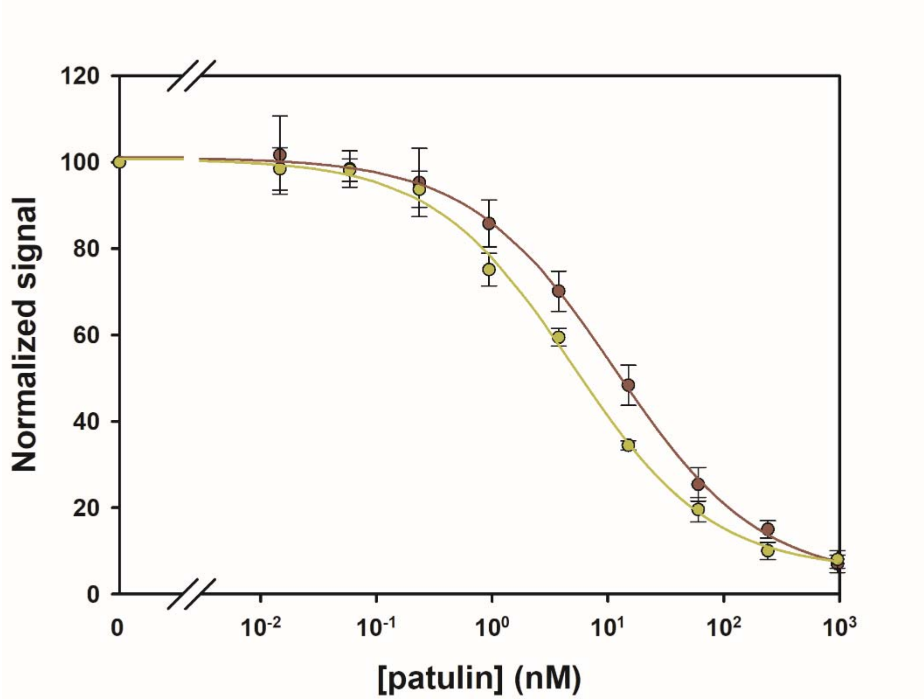

Figure S7. Recognition of patulin by rabbit polyclonal antibodies elicited by immunization with the bioconjugates BSA–**Ia** (green curve) and BSA–**Ib** (red curve). For the competitive step of the immunoassay, the patulin standards were prepared in PBS and 2% (v/v) of a 1 mg/mL of **7a** in water was added to each calibrator. The standards were incubated at rt for 30 min to quantitatively convert patulin to adduct **I**. Thereafter, the solutions were added to microtiter wells precoated with OVA–**Ia** or OVA–**Ib** at 1 µg/mL followed by the corresponding antiserum diluted 1/3000 in PBS-T. Error bars represent the standard deviation (n=3).

14. Monoclonal antibody generation, selection and purification

Six two-month old female Balb/c mice were inoculated by intraperitoneal injection with 100 µg of BSA–**Ia** conjugate in a 1:1 (v/v) emulsion (200 µL) between PB and Freund's adjuvant (complete for the first injection and incomplete for the second and third injections). After a resting period of at least three weeks from the third boost, a fourth injection in sterile phosphate buffer was administered four days before cell fusions.

For hybridoma generation, a modified procedure from the original protocol developed by Köhler and Milstein was followed which entails the cellular fusion of myeloma cell line P3-X63-Ag8.635 with B

lymphocytes isolated from the spleen of 2 equally-inoculated mice.[6] Once animal sacrifice had taken place, blood samples were obtained by intracardiac puncture, followed by spleen extirpation. Physical force using the piston of a sterile syringe was applied in order to extract cells from the spleen. Erythrocytes were lysed by osmotic shock employing 1 mL of lysis buffer at 4 °C for 1 min, and after washing with complete medium at 4 °C, the suspension was filtered to separate possible blood clots. Cellular fusion was carried out by mixing cultured myeloma cells with the isolated B lymphocytes at a 4:1 lymphocyte/myeloma ratio. The resulting mixture was washed 3 times with incomplete medium and then collected by centrifugation, and 1 mL of PEG 1500 at 37 °C was added for 1 min. Afterwards, the cell mixture was gradually diluted with 50 mL of complete medium and incubated for 30 min. After centrifugation, fused cells were distributed in 96-well culture plates at a density of 1.5×10^5 lymphocytes per well in 100 µL of DMEM containing 15% (v/v) FBS and incubated at 37 °C (5% CO_2, 95% RH). Twenty-four hours after plating, 100 µL of selection medium (DMEM supplemented with HAT containing 20% (v/v) FBS and 1% (v/v) HFCS) was added to each well and the plates were incubated under the same conditions for 12 days.

Antibody-producing cells were identified by a double screening procedure. Twelve days after cell fusions, hybridoma culture supernatants were first screened by differential antigen-coated competitive ELISA on microtiter plates coated with 0.1 µg/mL (100 µL per well) of the OVA–**Ia** bioconjugate. Fifty microliters of each supernatant was added to two adjacent wells of an ELISA plate, one containing 50 µL of PBS (blank) and the other one containing 50 µL of 200 nM adduct **I** in PBS. The ratio between the signals of both wells was used as the criterion for selecting the antibodies with the highest affinity. Fresh culture medium was added to the selected wells, and the next day they were revaluated by checkerboard competitive ELISA. This second screening assay was carried out with two coating concentrations of the OVA–**Ia** conjugate (0.01 and 0.1 µg/mL), four supernatant dilutions (1/10, 1/50, 1/250, and 1/1250), and three analyte levels (0, 10, and 100 nM). Selected hybridomas were cloned by limiting dilution in cloning medium (DMEM containing 20% (v/v) FBS and supplemented with HT and 1% (v/v) HFCS), and stable antibody-producing clones were expanded and cryopreserved in liquid nitrogen.

Hybridoma expansion was carried out in Petri dishes for 7-10 days. Cells were removed by centrifugation and the supernatants were precipitated with one volume of saturated ammonium sulphate. To purify the monoclonal antibodies, precipitated supernatants were centrifuged (3800 × g, 20 min), the pellet was

---

[6] (*a*) Kearney, J. F., Radbruch, A., Liesegang, B. & Rajewsky, K. A new mouse myeloma cell line that has lost immunoglobulin expression but permits the construction of antibody-secreting hybrid cell lines. *J. Immunol.* **123**, 1548–1550 (1979). (*b*) Köhler, G. & Milstein, C. Continuous cultures of fused cells secreting antibody of predefined specificity. *Nature* **256**, 495–497 (1975). (*c*) Nowinski, R. C., Lostrom, M. E., Tam, M. R., Stone, M. R. & Burnette, W. N. The isolation of hybrid cell lines producing monoclonal antibodies against the p15(E) protein of ecotropic murine leukemia viruses. *Virology* **93**, 111–126 (1979). (*d*) Mercader, J. V., Suárez-Pantaleón, C., Agulló, C., Abad-Somovilla, A. & Abad-Fuentes, A. Production and characterization of monoclonal antibodies specific to the strobilurin pesticide pyraclostrobin. *J. Agric. Food Chem.* **56**, 7682–7690 (2008).

dissolved in 8-10 mL of 20 mM phosphate buffer, pH 7.4, and filtered through a nitrocellulose membrane (0.45 µm pore diameter). The resulting filtrate was purified by affinity chromatography with a 5 mL HiTrap Protein G HP column, and the antibody-containing fractions were gathered and the buffer was exchanged to 20 mM phosphate buffer, pH 7.4, using Amicon Ultra 4 10k centrifugal devices. Final antibody concentration was calculated using the absorption at 280 nm and a molar extinction coefficient of 1.4 L g^{-1} cm^{-1}. The purified antibodies were precipitated with 1 volume of saturated ammonium sulphate for long-term storage at 4 °C. Working aliquots were prepared by diluting the precipitate with PBS containing 1% (w/v) BSA and 0.05% thimerosal and were also stored at 4 °C.

15. Characterization of the monoclonal antibodies by competitive ELISA

Antibody-coated direct competitive ELISA. Microtiter plates were coated by adding 100 µL per well of the antibody solution in 50 mM carbonate buffer, pH 9.6. After overnight incubation at 4 °C, the plates were washed four times with a 150 mM NaCl solution. The competitive assay was carried out by adding 50 µL per well of the analyte in phosphate-buffered saline (PBS) plus 50 µL per well of the HRP–hapten bioconjugate in PBS-T (PBS containing 0.05% v/v Tween 20), and the plates were incubated 1 h at rt. After plate washing, the signal was generated by adding 100 µL per well of the enzyme substrate solution. Following incubation during 10 min at rt, the reaction was stopped by adding 100 µL per well of 1 M H_2SO_4, and the absorbance was immediately read.

Conjugate-coated indirect competitive ELISA. Microtiter plates were coated by adding 100 µL per well of the corresponding OVA–hapten conjugate solution in 50 mM carbonate buffer, pH 9.6. After overnight incubation at rt, the plates were washed four times with a 150 mM NaCl solution. The competitive assay was carried out by adding 50 µL per well of the analyte in PBS plus 50 µL per well of antibody solution in PBS-T, and the plates were incubated 1 h at rt. After plate washing, 100 µL per well of the enzyme-labelled secondary antibody (RAM-HRP 1/2000) in PBS-T was added and incubated at rt for an additional hour. The plates were washed again and the signal was generated by adding 100 µL per well of the enzyme substrate solution. Following incubation during 10 min at rt, the reaction was stopped by adding 100 µL per well of 1 M H_2SO_4, and the absorbance was immediately read.

Table S2. Characterization of the monoclonal antibodies for patulin binding by competitive ELISA (n=3).

	**Direct Format**			**Indirect Format**					
	HRP–**Ia**			OVA–**Ia**			OVA–**Ib**		
mAb	[mAb][a]	[HRP][a]	IC_{50}[b]	[mAb]	[OVA]	IC_{50}	[mAb]	[OVA]	IC_{50}
#13	1000	100	0.8	300	100	6.7	100	300	4.0
#17	100	300	0.7	100	100	3.5	100	300	7.0
#116	–[c]	–	–	100	100	6.5	30	1000	2.9

[a] Antibody and bioconjugate concentration values are in ng/mL. [b] Values are expressed in nM units. [c] No response.

For the competitive step of the immunoassays, the patulin standards were prepared in PBS and 2% (v/v) of a 1 mg/mL of **7a** in water was added to each calibrator. After incubation at rt for 30 min to quantitatively convert patulin to adduct **I**, standards were assayed by ELISA.

16. Immunochromatographic assays

Immunostrips were prepared by dispensing the immunoreagents on a nitrocellulose membrane (300 × 25 mm) at a flow rate of 0.5 µL/cm employing a BioDot ZX1010 machine. The test line (TL) was formed by dispensing the BSA–**Ia** conjugate at 1 mg/mL in 100 mM phosphate buffer, pH 7.4, containing 150 mM NaCl, while the control line (CL) was formed by dispensing goat anti-mouse immunoglobulins also at 1 mg/mL in the same buffer. The distance of the TL and CL from the base of the membrane was 10 mm and 15 mm, respectively. Once dried for 1 h at rt, the membrane was fixed, 14 mm from the base, to the backing card (300 × 78 mm). A nitrocellulose sample pad (300 × 17 mm) and an absorbent pad (300 × 43 mm) were incorporated overlapping 3 mm and 4 mm with the membrane, respectively. Lastly, a Biodot guillotine was used to cut the assembled components in to 4 × 78 mm immunostrips.

The mAb #17 was coupled to colloidal gold as follows. The antibody was added to a final concentration of 1 µg/mL to commercial 40 nm GAM-modified gold nanoparticles (OD = 10) diluted 1/10 in 75 mM Tris buffer, pH 7.4, containing 0.05% (v/v) Tween 20, and the mixture was incubated for 1 h at rt. For running the assay, 25 µL of nanoparticles and 75 µL of standard or sample solution, both in 75 mM Tris buffer, pH 7.4, with 0.05% /v/v) Tween 20, were added to a polystyrene well and incubated for 5 min at rt. The immunostrip was then vertically dipped into the well for 10 min, the sample pad was removed, and the membrane was dried under a cold airflow. The signals at the TL and CL were acquired with a flatbed scanner and processed using ImageJ (version 1.52a) to calculate the TL/CL ratio.

17. Determination of patulin in apple juice samples

Table S3. Determination of patulin in contaminated apple juices from proficiency testing programmes with the immunochromatographic assay based on the antibody #17 and the bioconjugate BSA–Ia.[a]

Supplier	Sample code	Assigned value	Satisfactory range	Value found
FAPAS	T1668QC	9.3	5.2 – 13.4	8.5 ± 1.0
	TYG060RM	20.1 ± 1.4	-[b]	15.6 ± 1.1
	T1672QC	39.6	22.2 – 57.0	36.3 ± 5.6
LGC standards	QBS_BV280_520	29.3 ± 8.1	13.2 – 45.4	33.4 ± 8.4

[a] Values are in ng/mL from three independent determinations. [b] Not provided by the supplier.

18. Copies of NMR spectra

^{13}C NMR spectrum (126 MHz) of adduct **I** in acetone-d_6

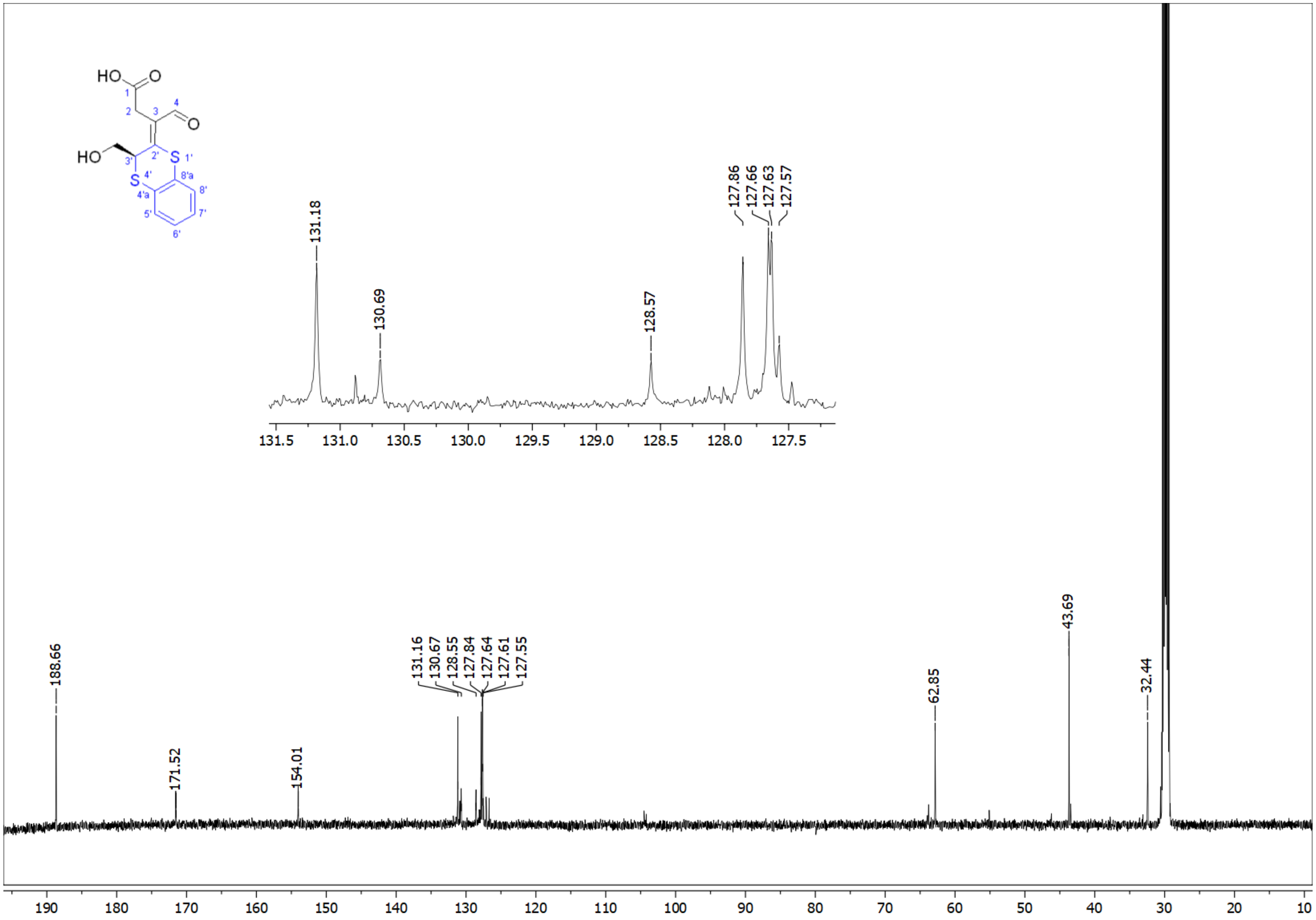

^{1}H NMR spectrum (400 MHz) of methyl ester of adduct **I** in $CDCl_3$

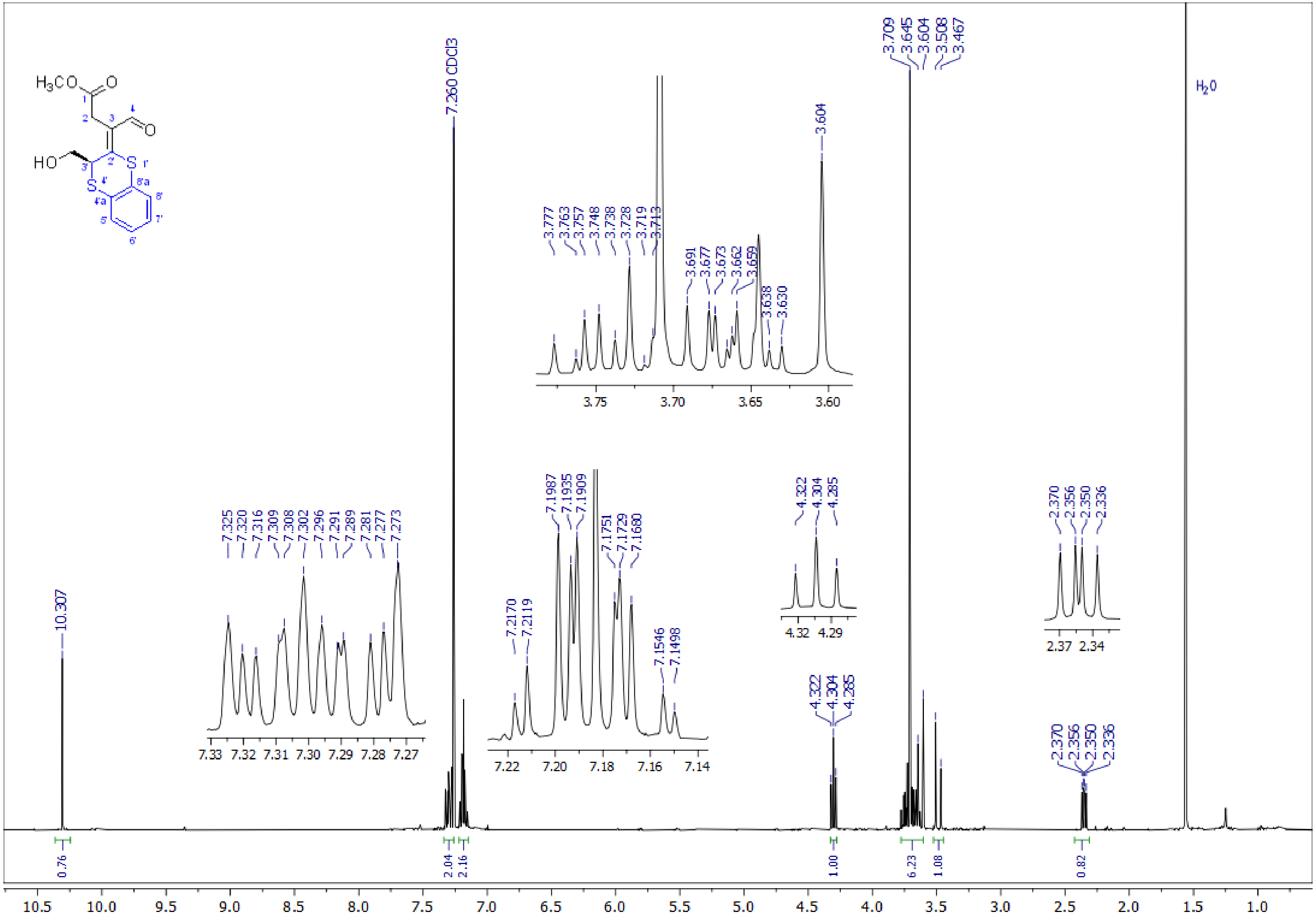

^{13}C NMR spectrum (101 MHz) of methyl ester of adduct **I** in $CDCl_3$

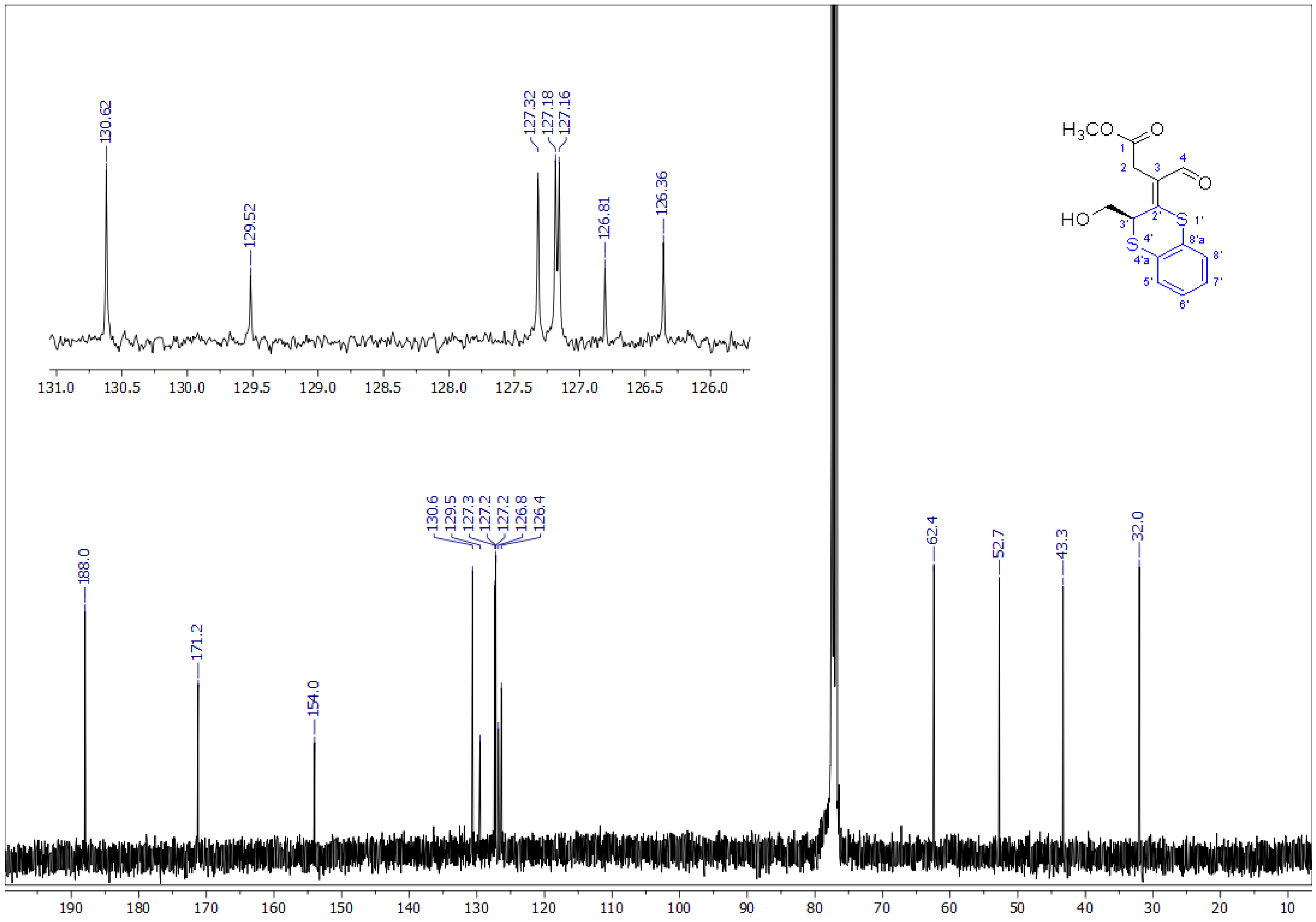

2D edited-HSQC spectrum of methyl ester of adduct **I** in $CDCl_3$

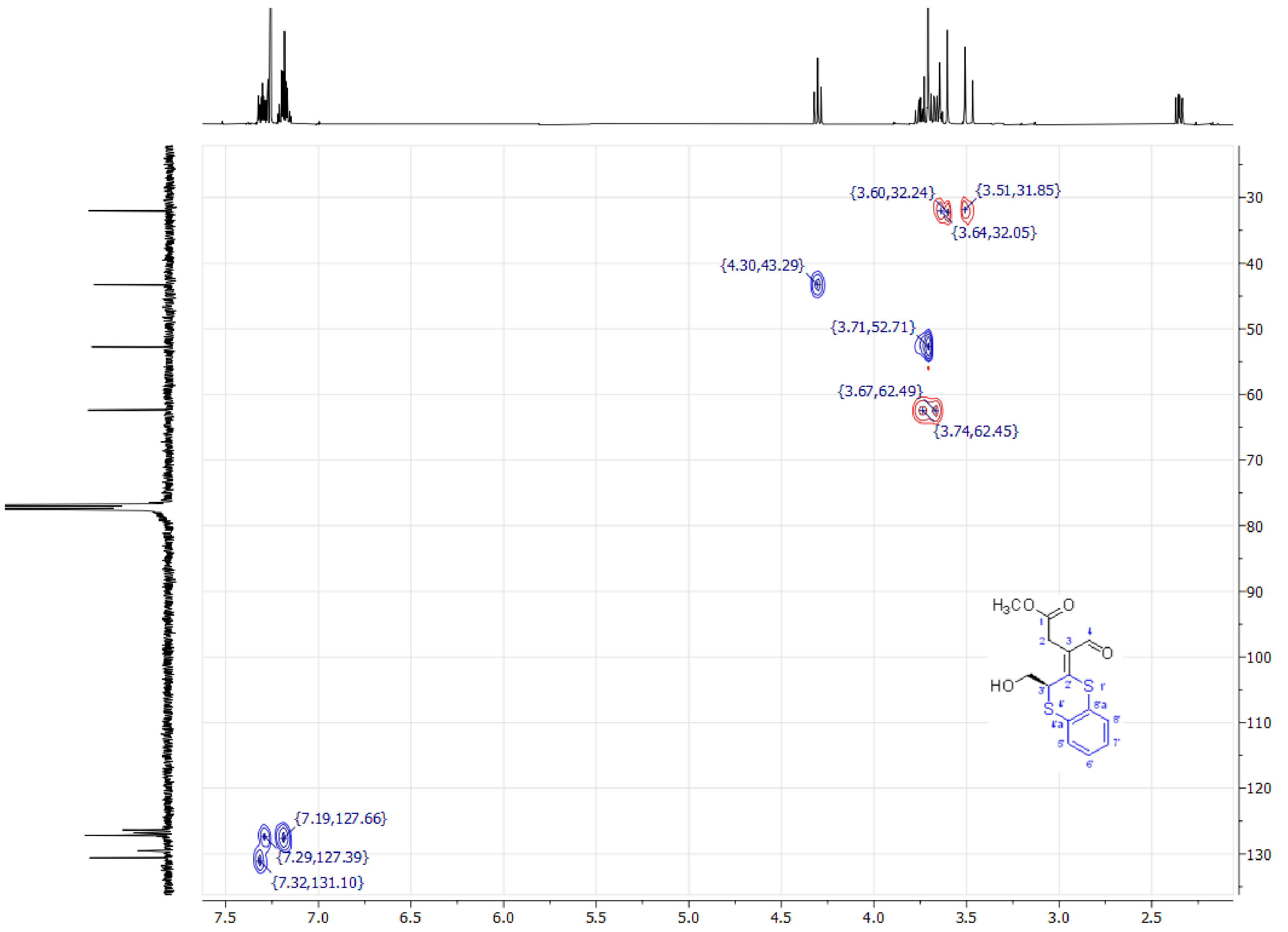

2D COSY spectrum of methyl ester of adduct **I** in $CDCl_3$

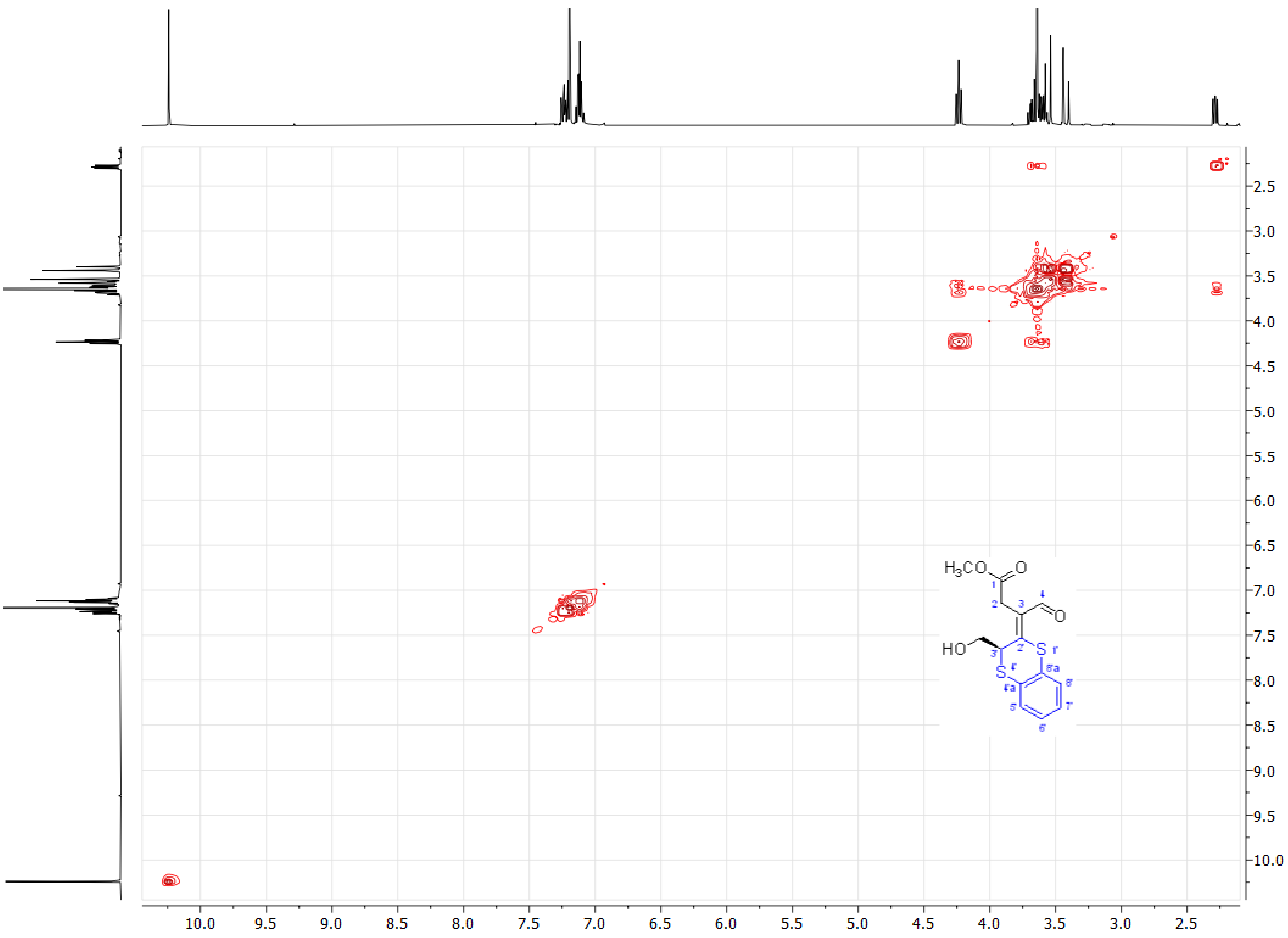

2D HMBC spectrum of methyl ester of adduct **I** in $CDCl_3$

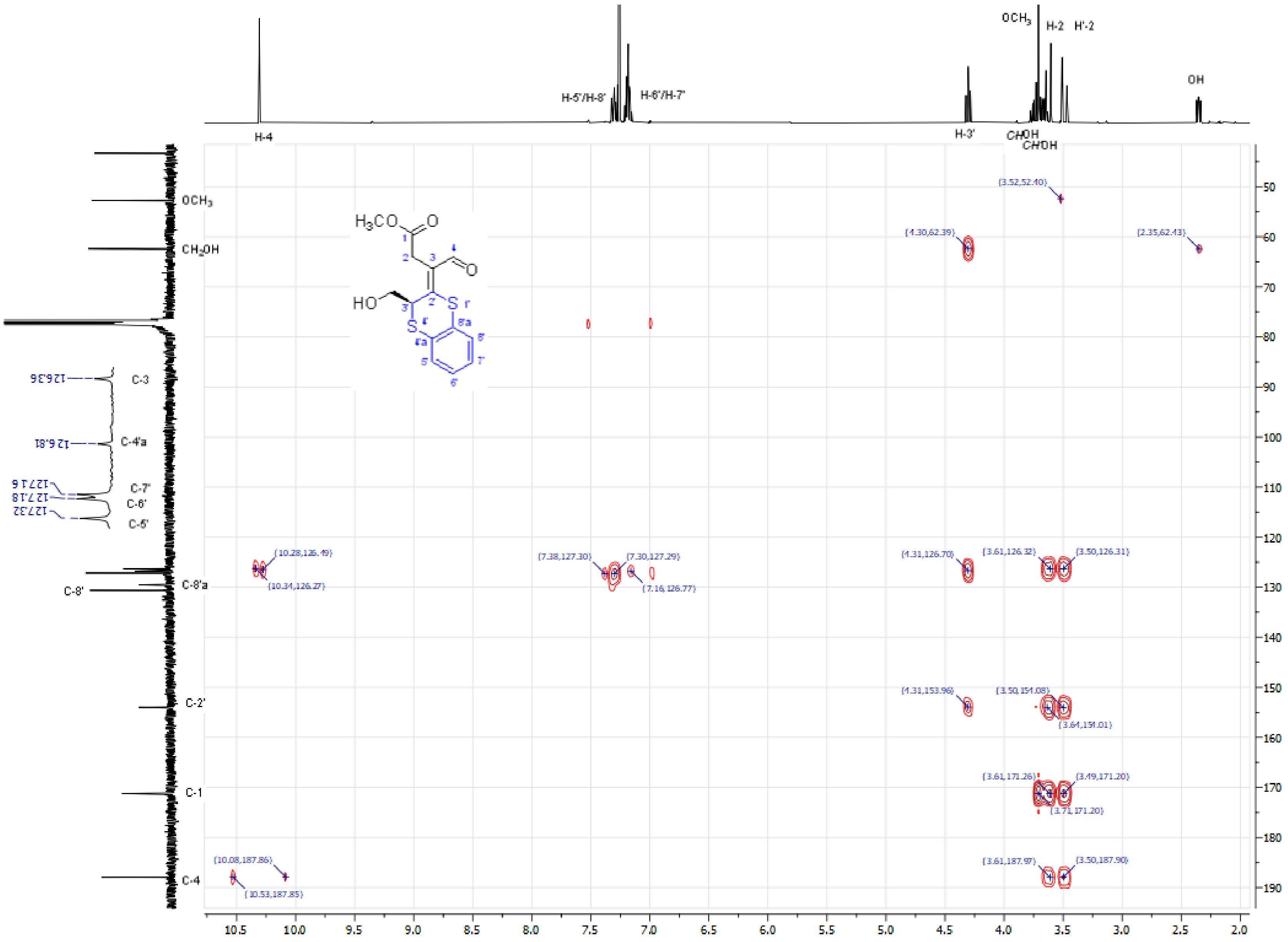

2D HMBC spectrum of methyl ester of adduct **I** in $CDCl_3$

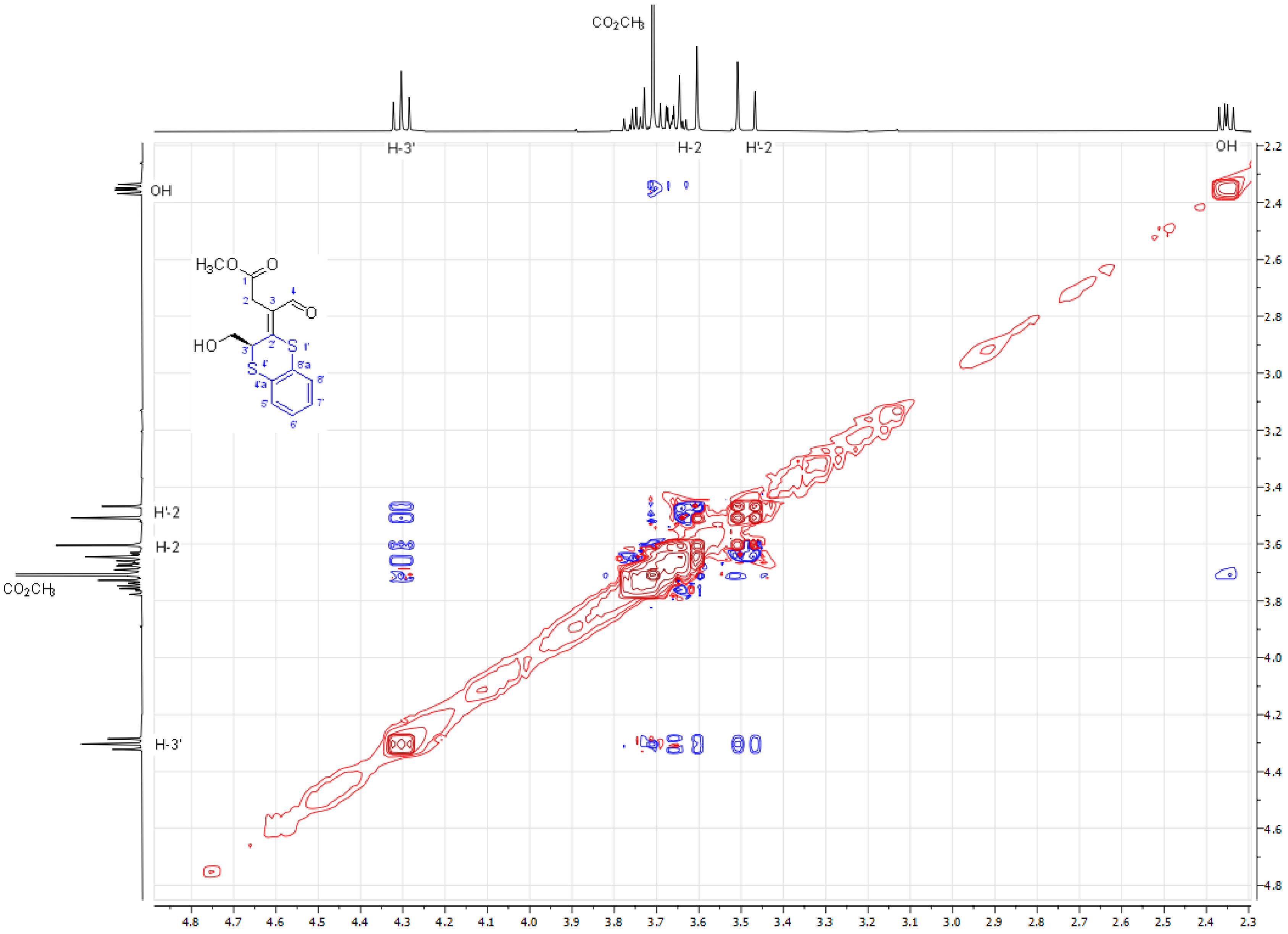

^{1}H NMR spectrum (500 MHz) of the NHS ester of adduct **I** in $CDCl_3$

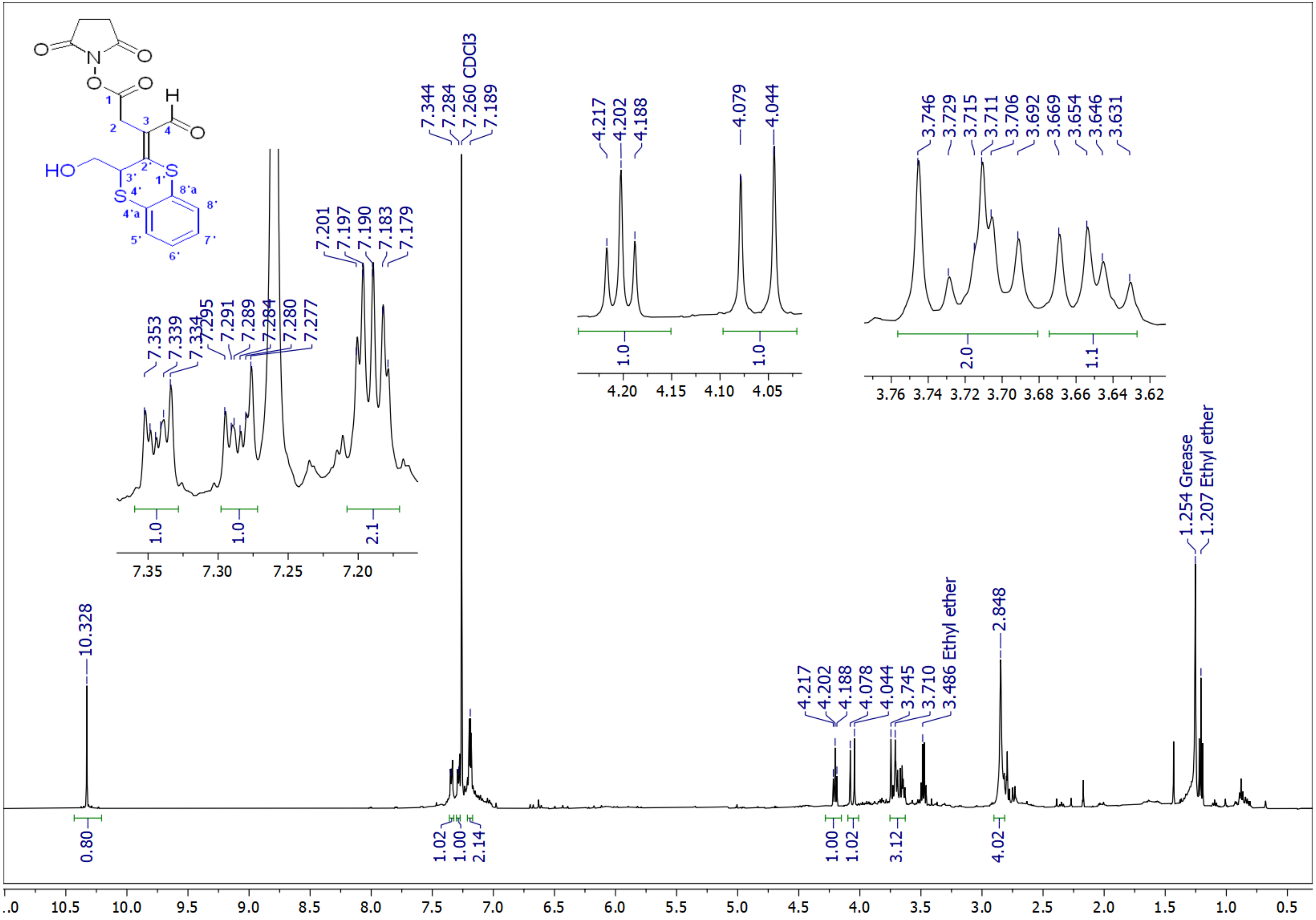

^{1}H NMR spectrum (400 MHz) of adduct **II** in $CDCl_3$

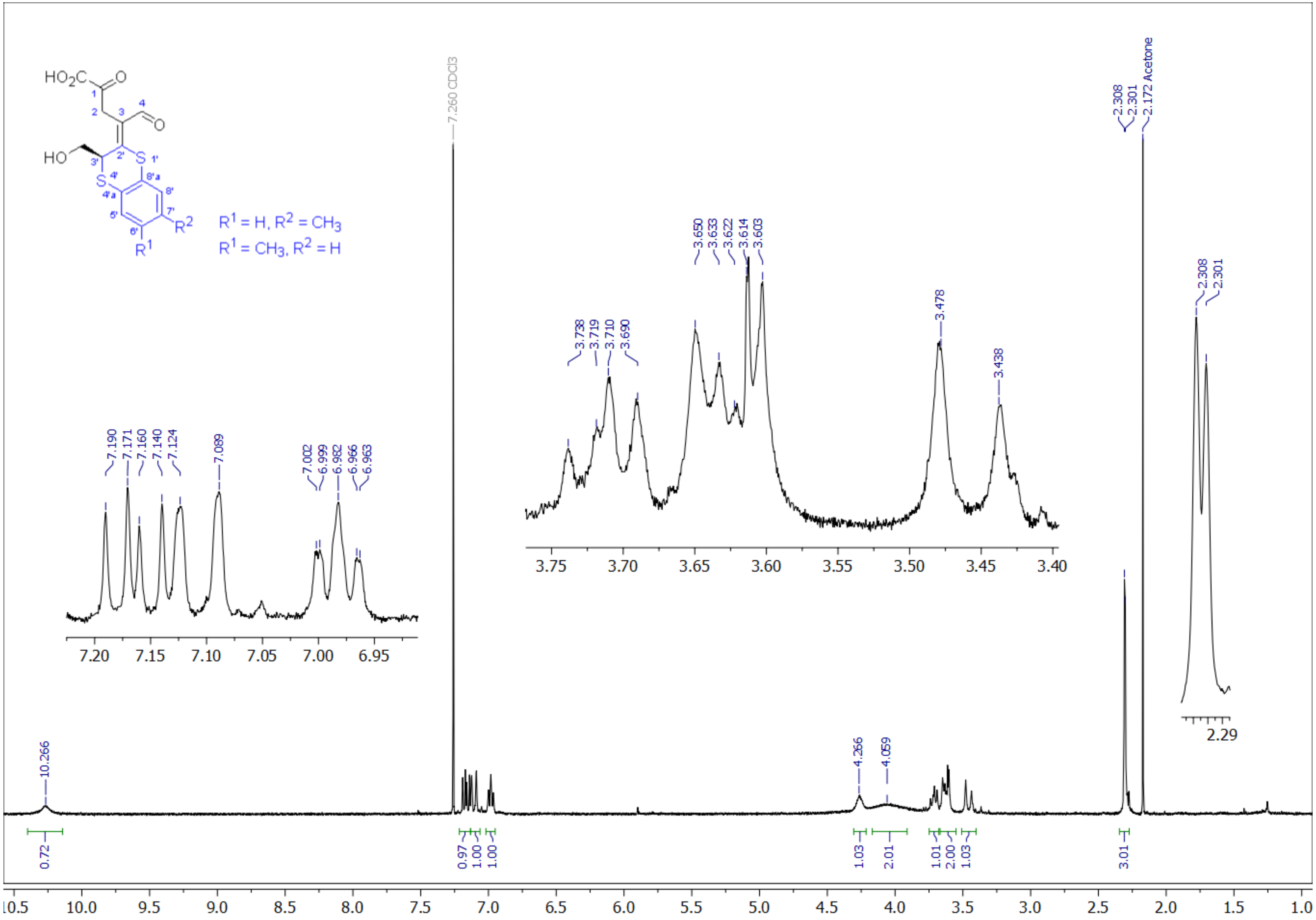

^{13}C NMR spectrum (126 MHz) of adduct **II** in $CDCl_3$

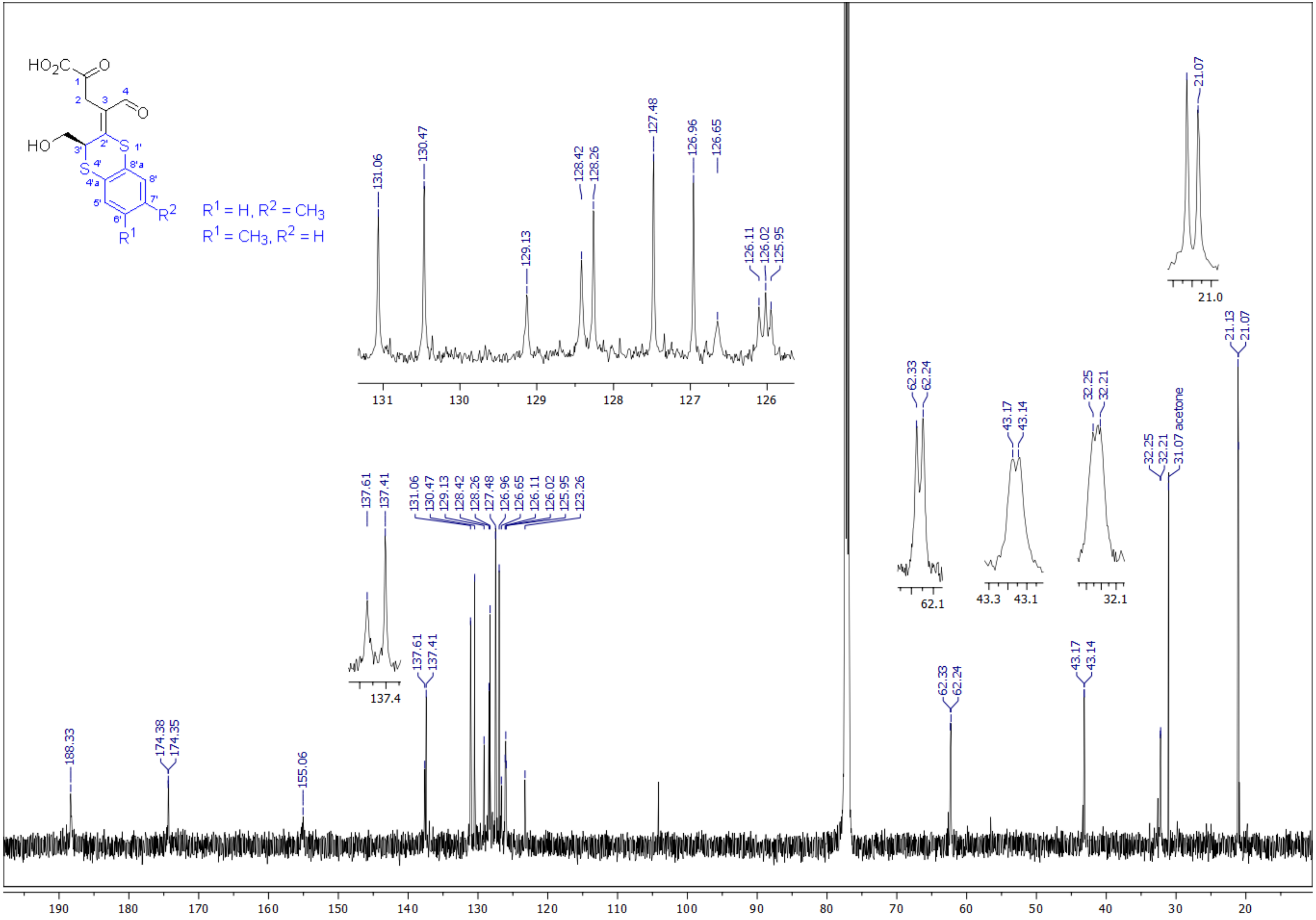

^1H NMR spectrum (300 MHz) of adduct **II** methyl ester in $CDCl_3$

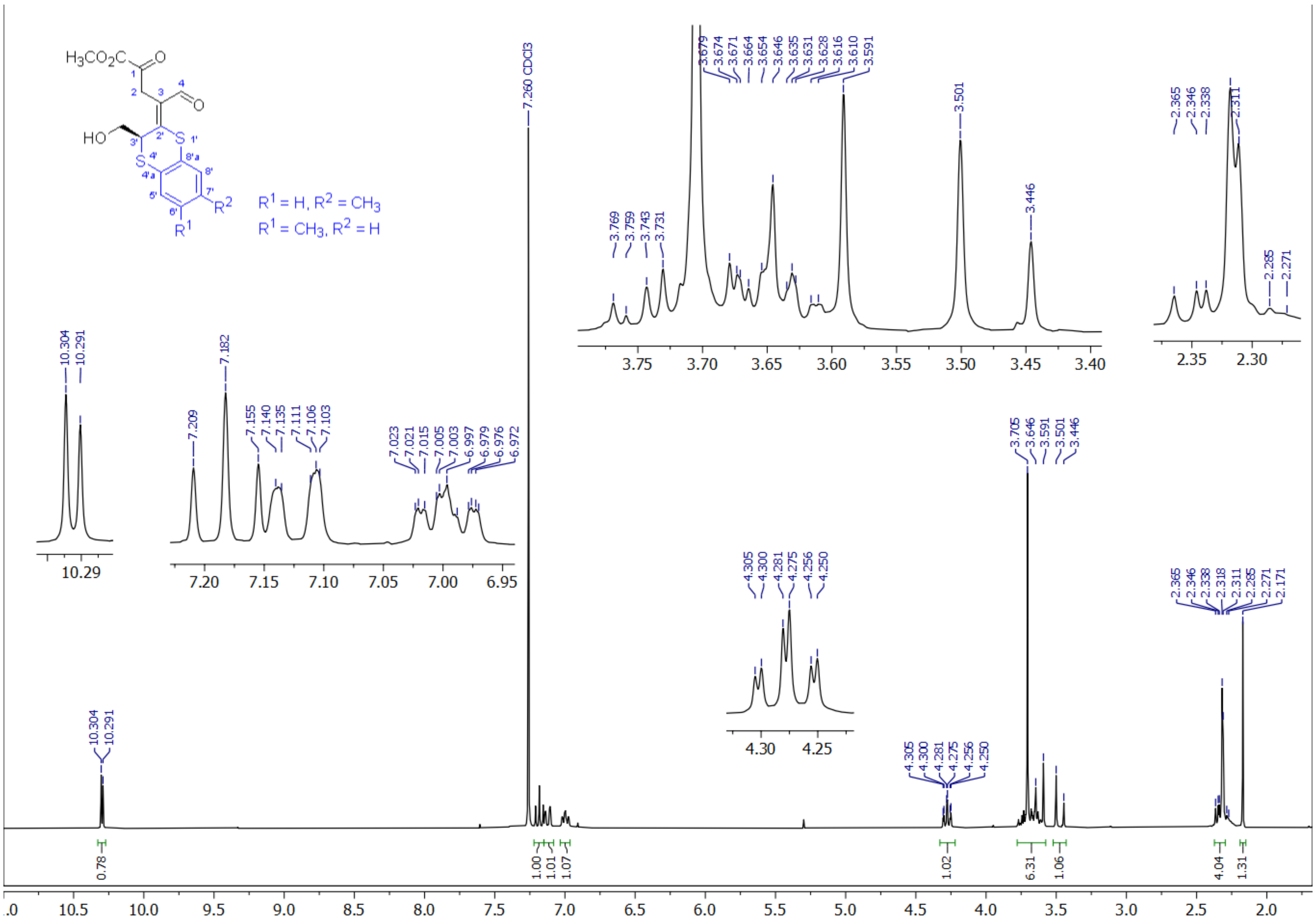

^1H NMR spectrum (300 MHz) of adduct **III** in acetone-d_6

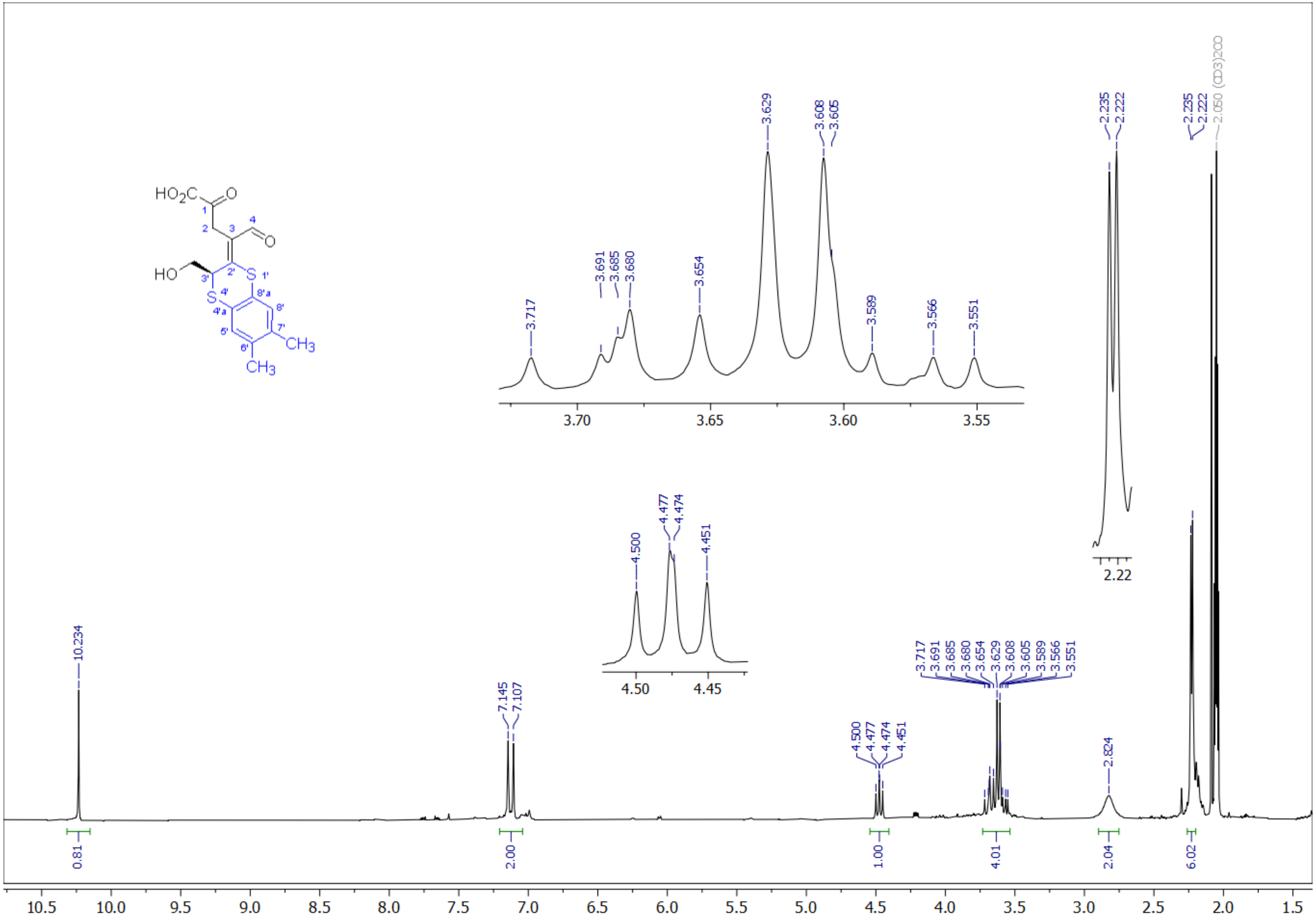

^{13}C NMR spectrum (75 MHz) of adduct **III** in acetone-d_6

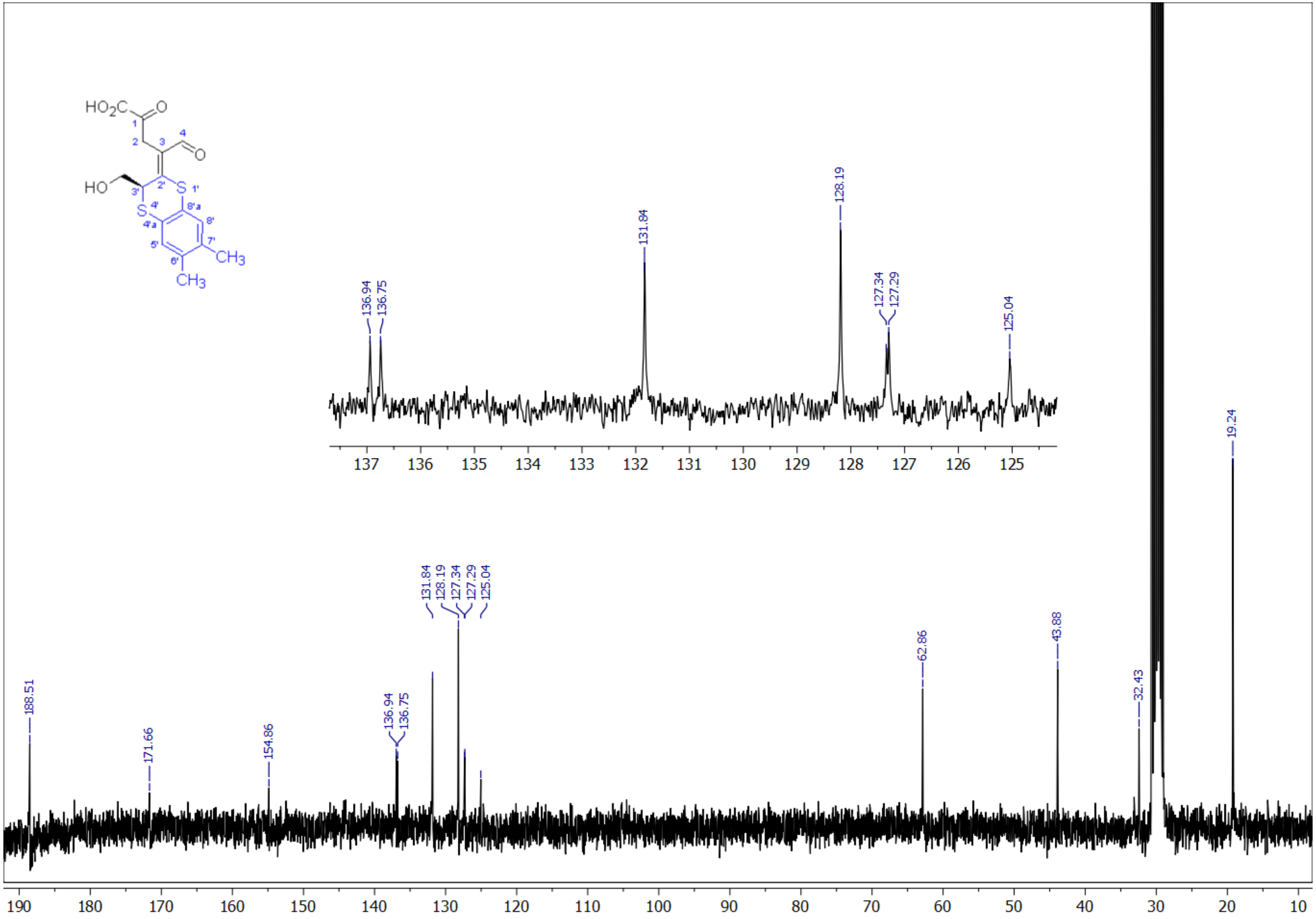

^{1}H NMR spectrum (300 MHz) of adduct **IV** in acetone-d$_6$

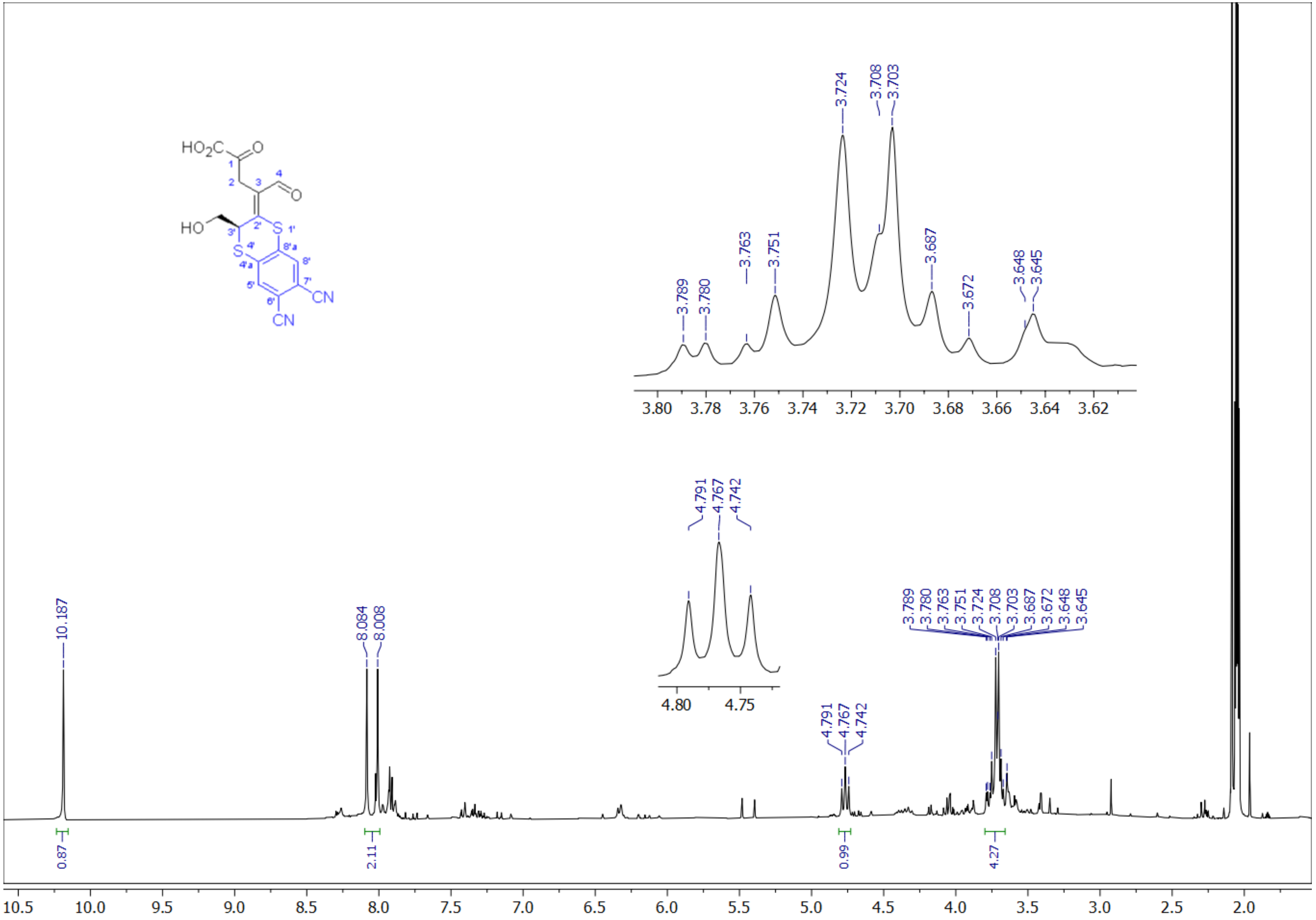

^{13}C NMR spectrum (75 MHz) of adduct **IV** in acetone-d_6

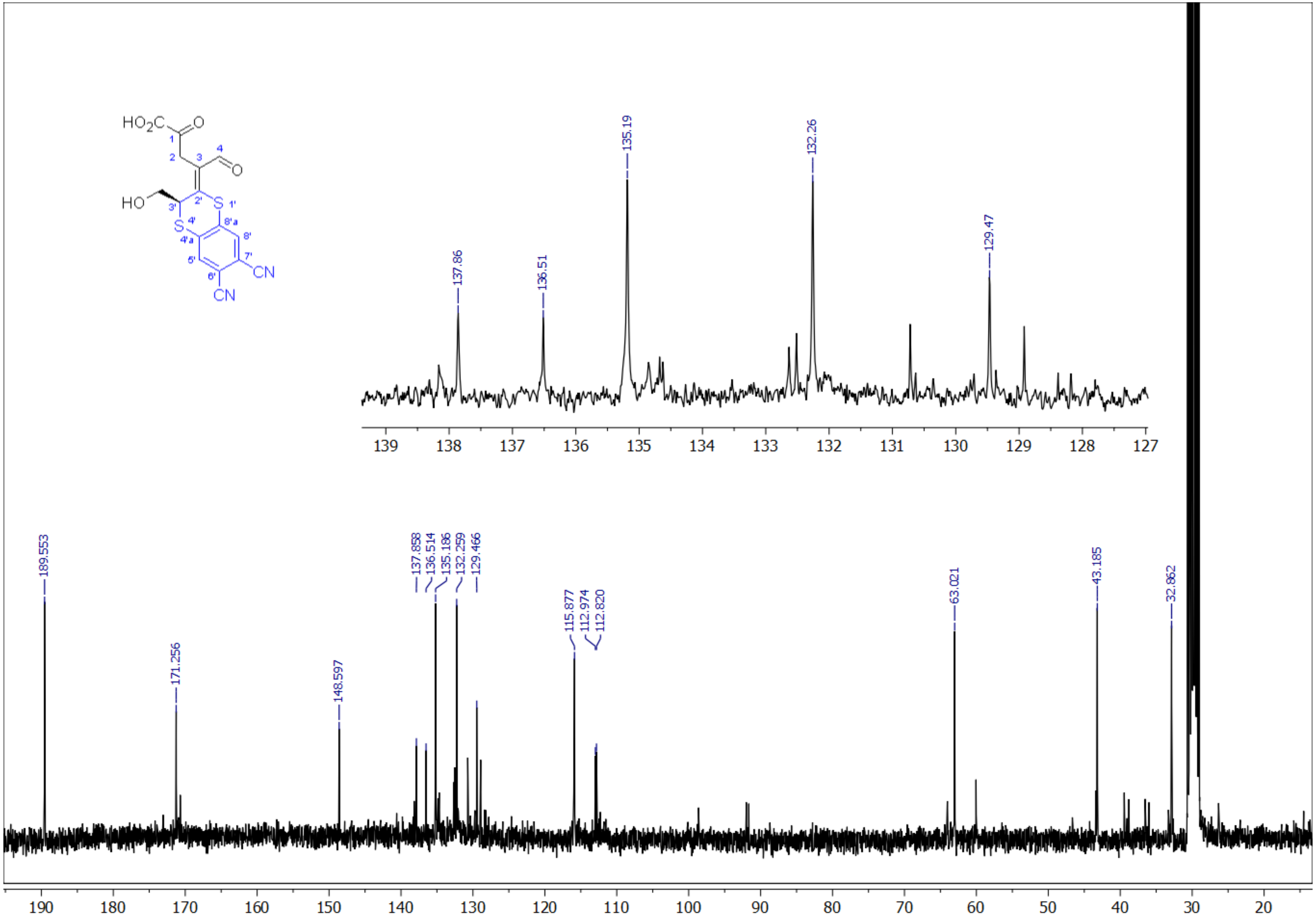

^{1}H NMR spectrum (300 MHz) of adduct **S1** in Methanol-d$_4$

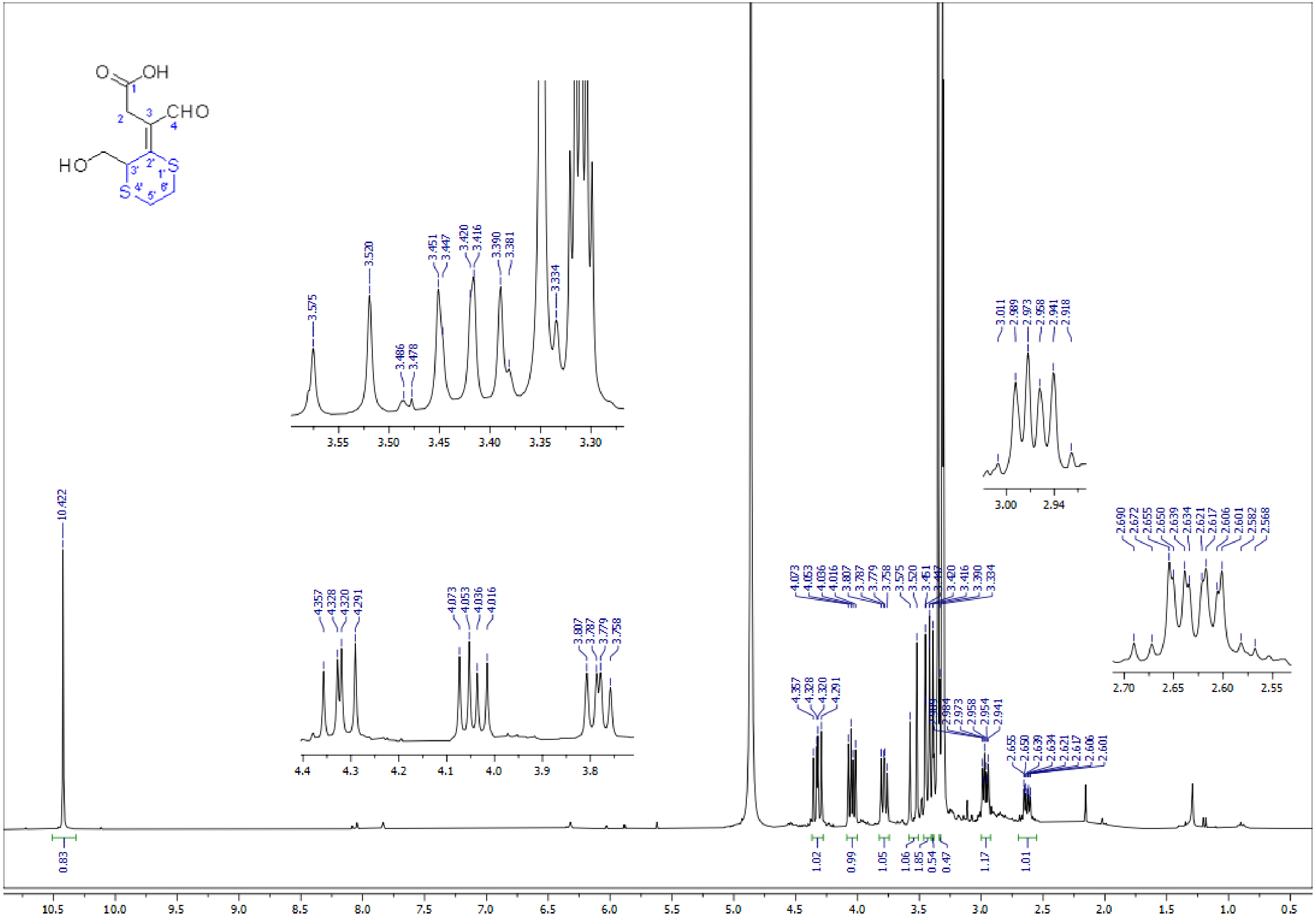

2D COSY spectrum expansion (from 4.5 to 2.5 ppm) of adduct **S1** in Methanol-d_4

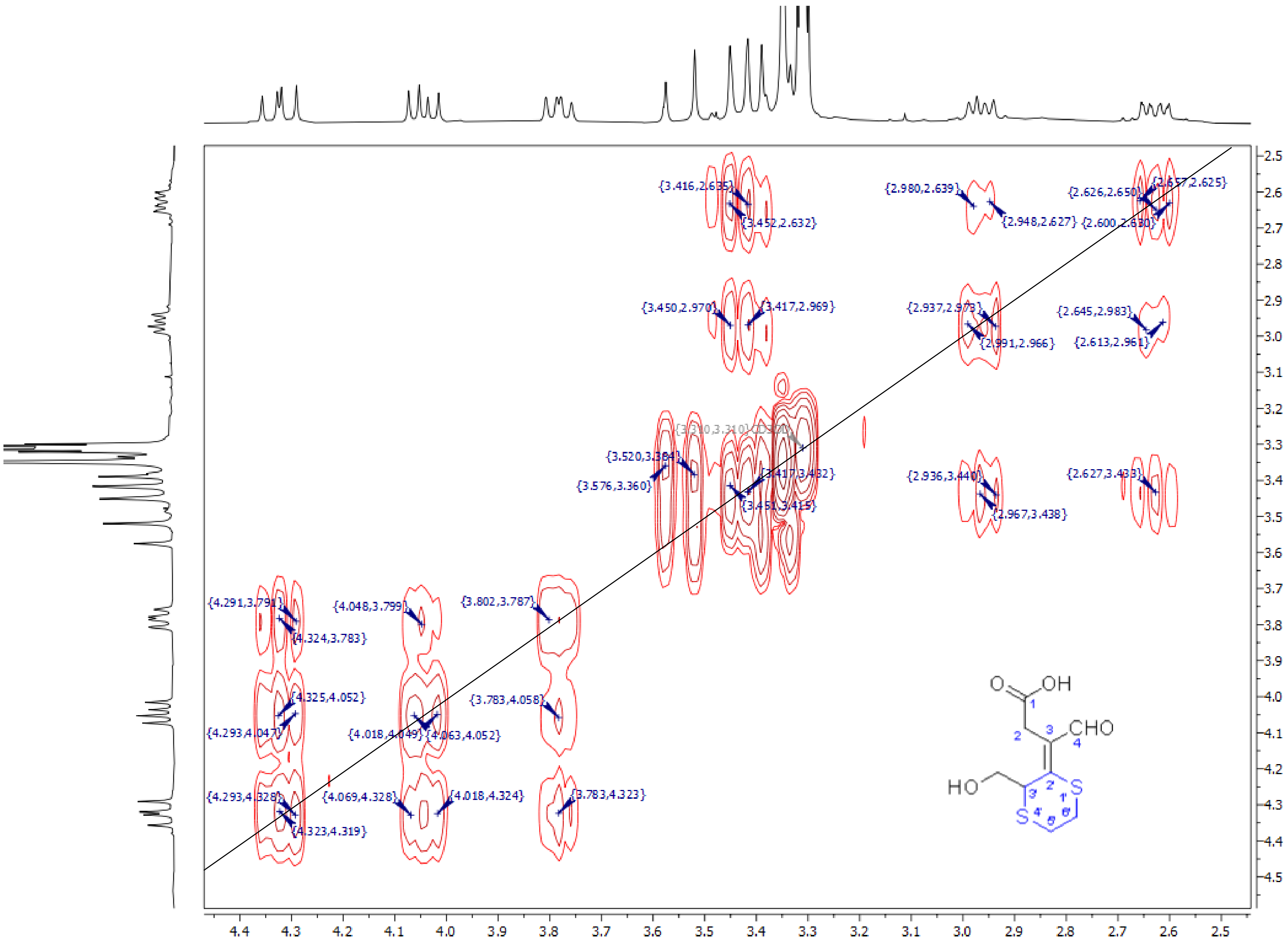

^{1}H NMR spectrum (500 MHz) of hapten **Ia** in acetone-d_6

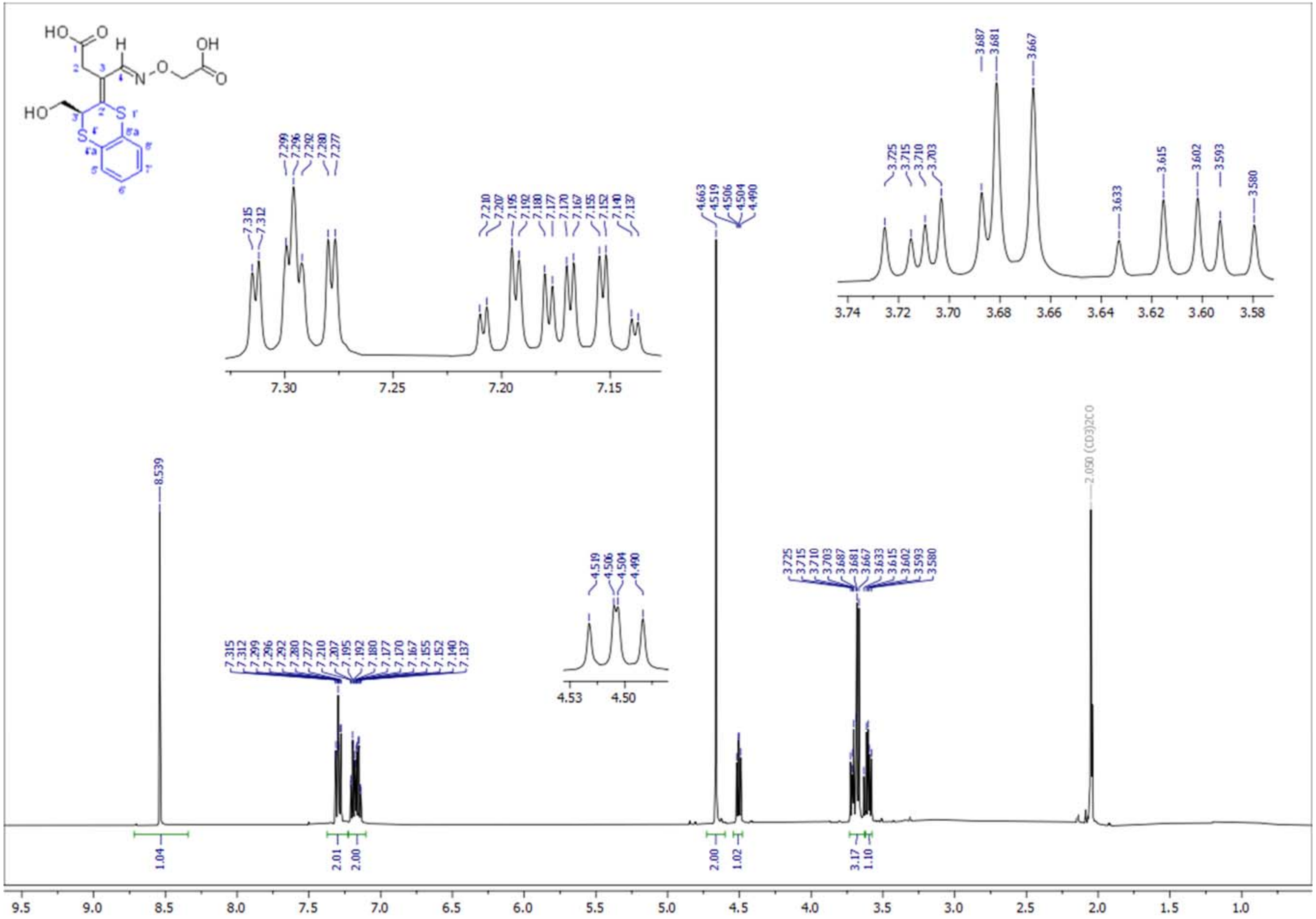

^{13}C NMR spectrum (126 MHz) of hapten **Ia** in acetone-d_6

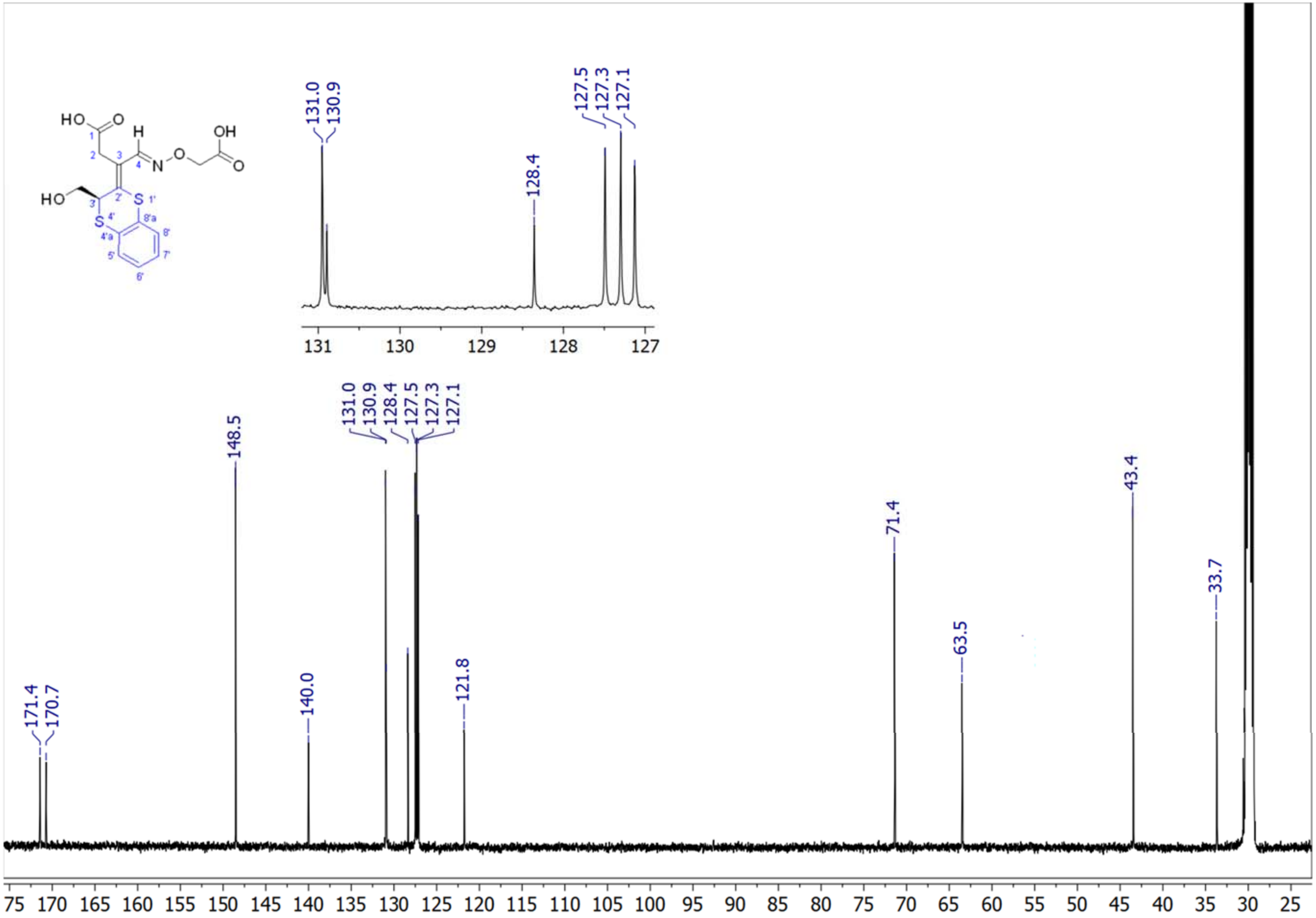

^{1}H NMR spectrum (500 MHz) of dimethyl ester of hapten **Ia** in $CDCl_3$

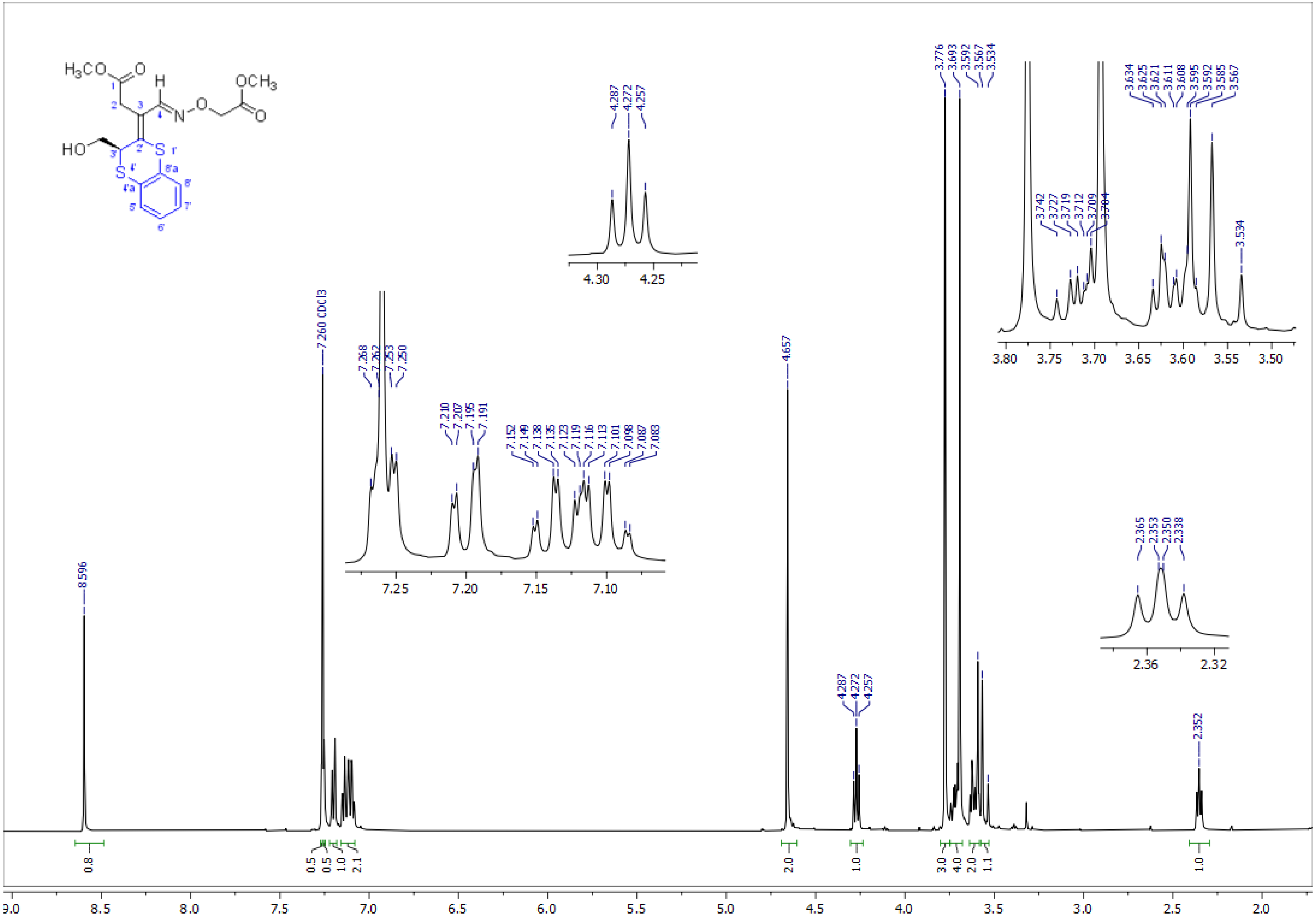

^{13}C NMR spectrum (126 MHz) of dimethyl ester of hapten **Ia** in $CDCl_3$

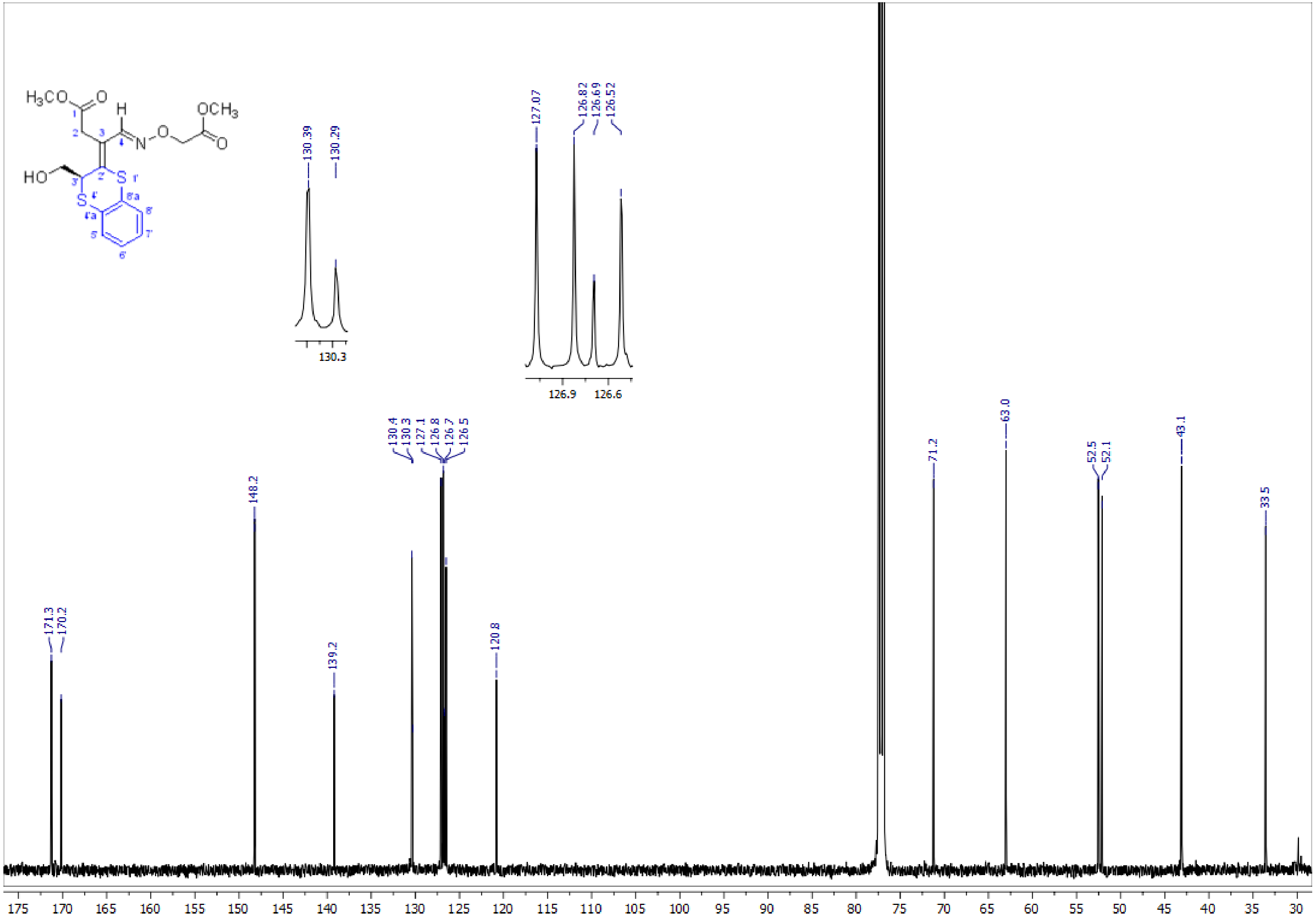

2D HMBC spectrum of dimethyl ester of hapten **Ia** in $CDCl_3$

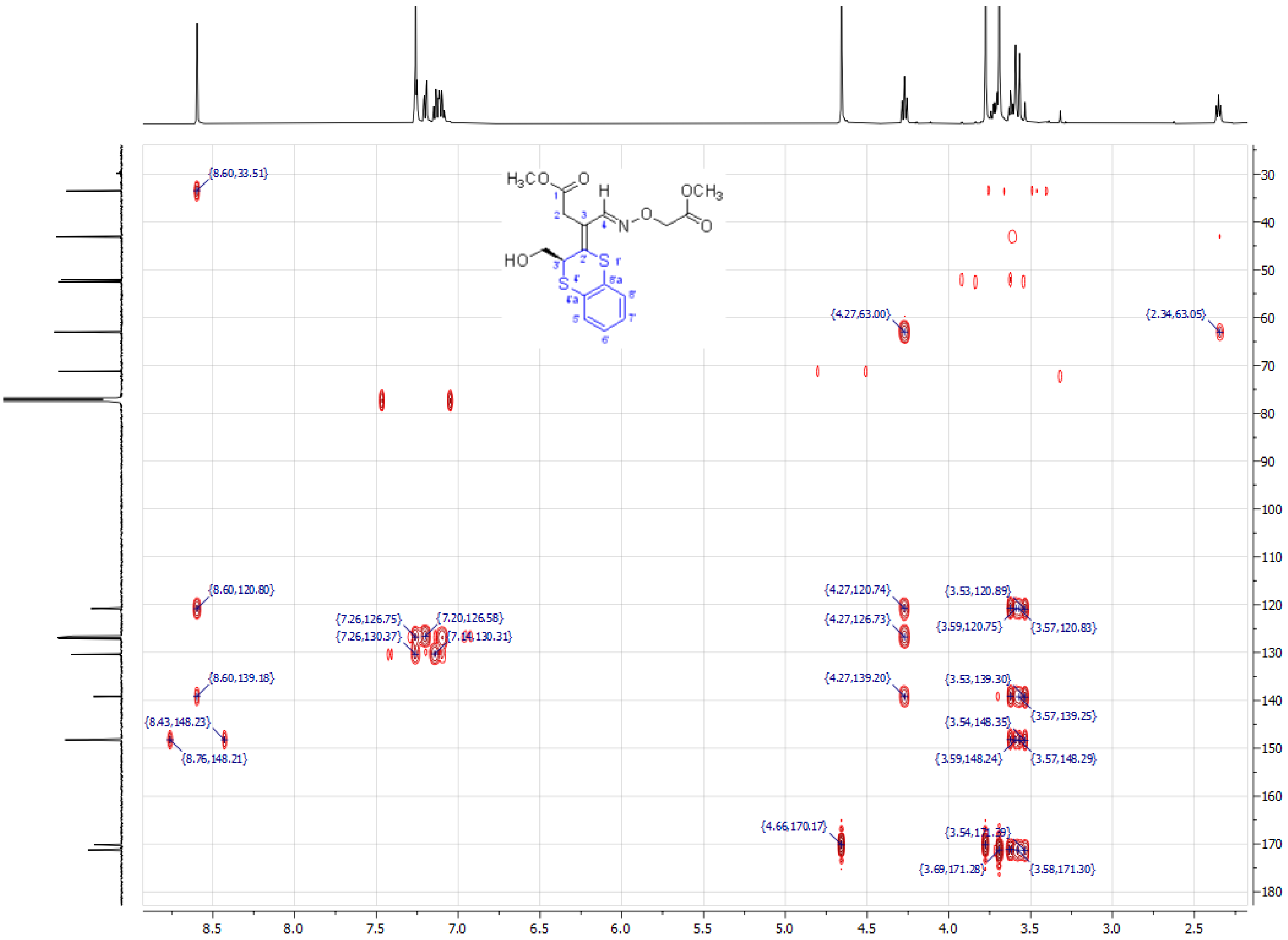

^{1}H NMR spectrum of *bis*-NHS ester of hapten **Ia** in $CDCl_3$

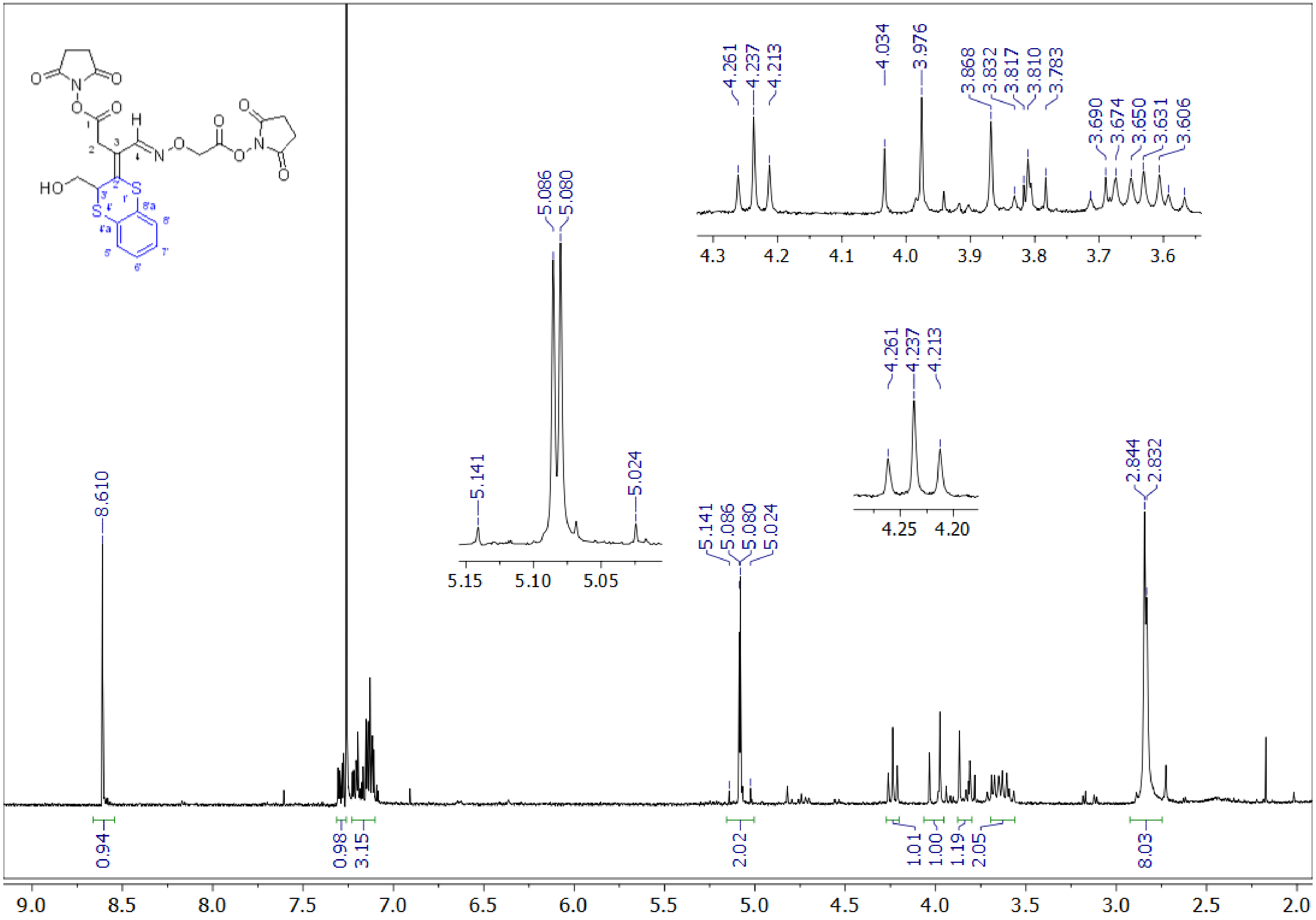

^1H NMR spectrum (300 MHz) of hapten **Ib** in acetone-d_6

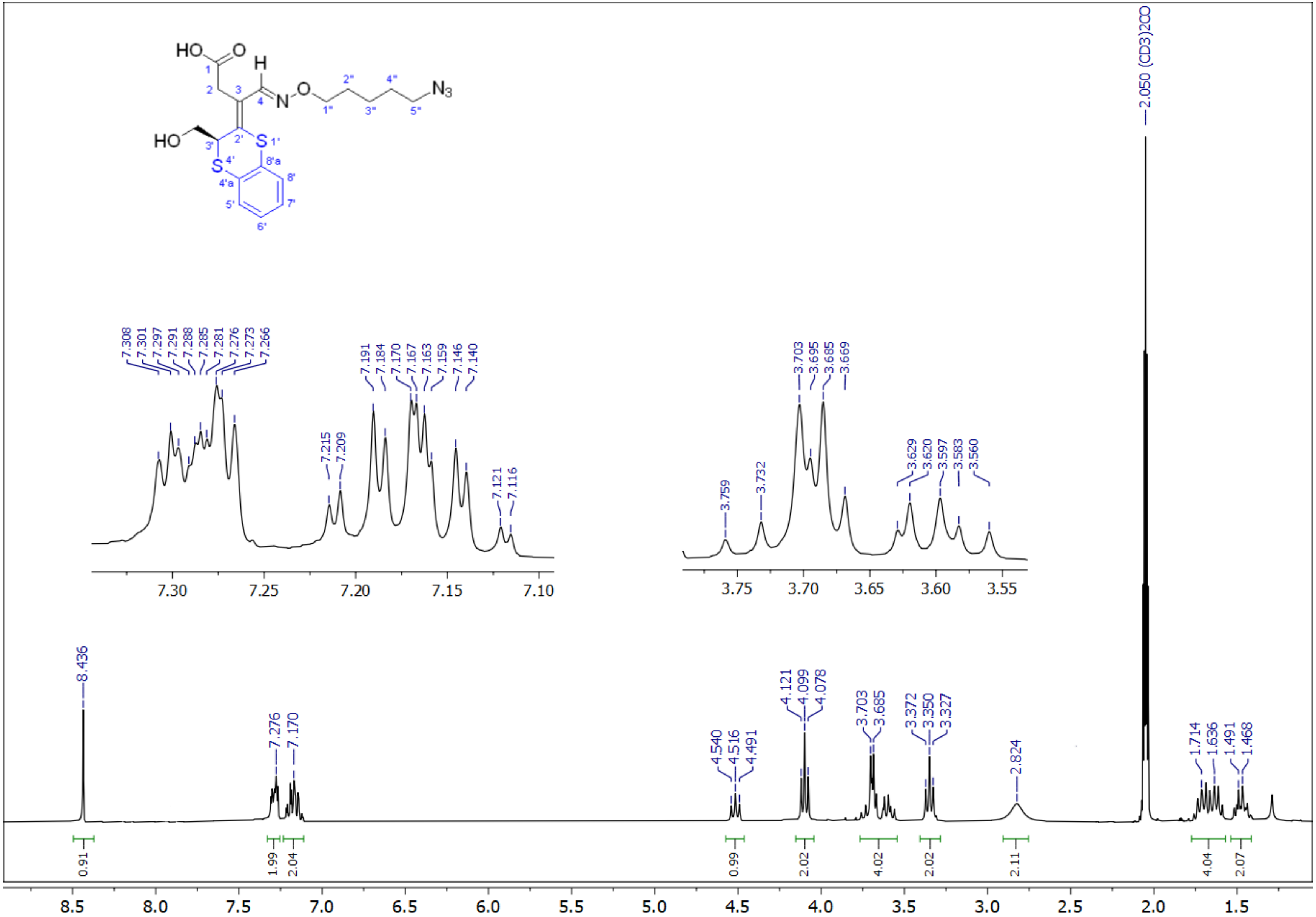

^{13}C NMR spectrum (126 MHz) of hapten **Ib** in acetone-d6

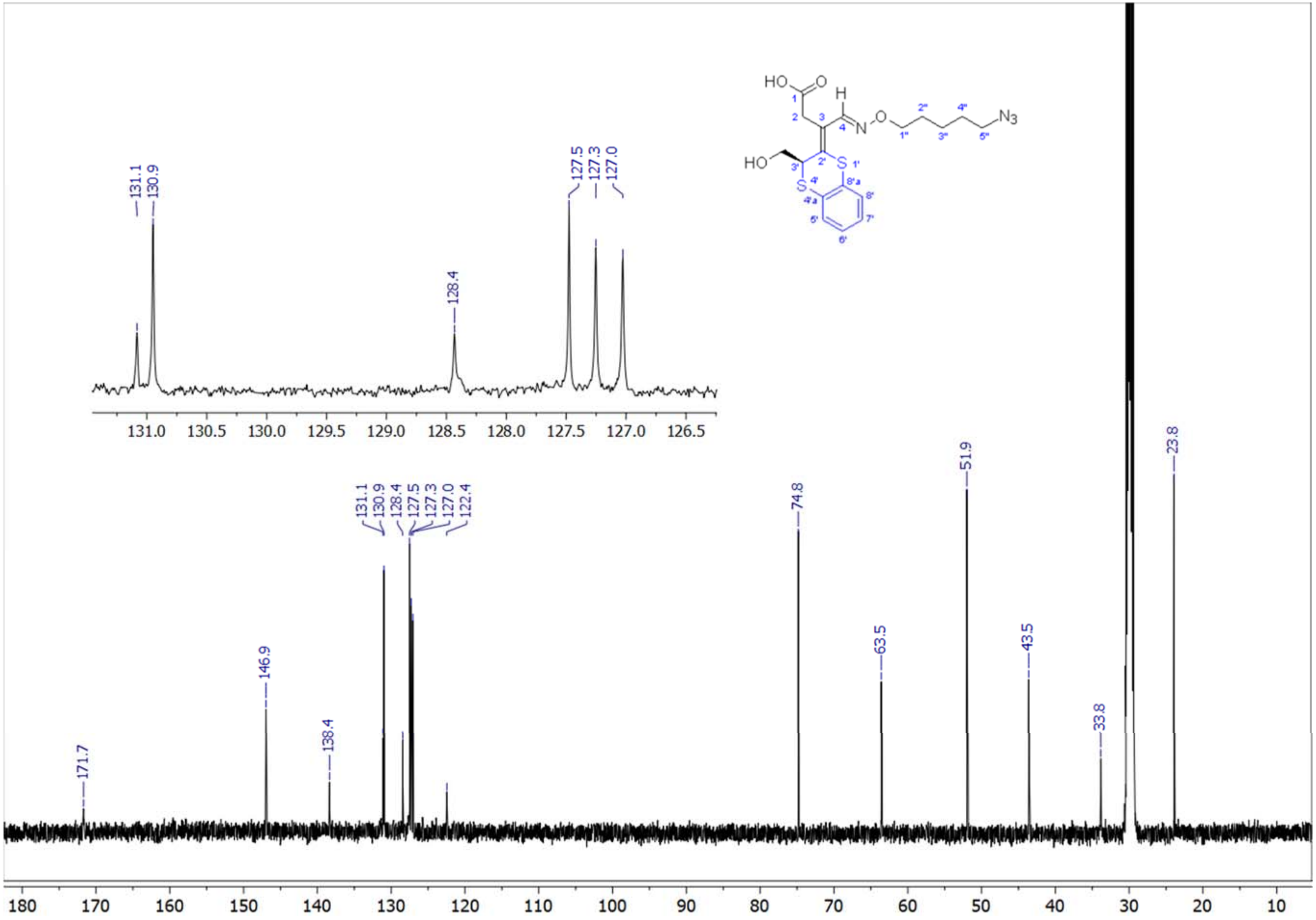

2D edited-HSQC spectrum of hapten **Ib** in acetone-d_6

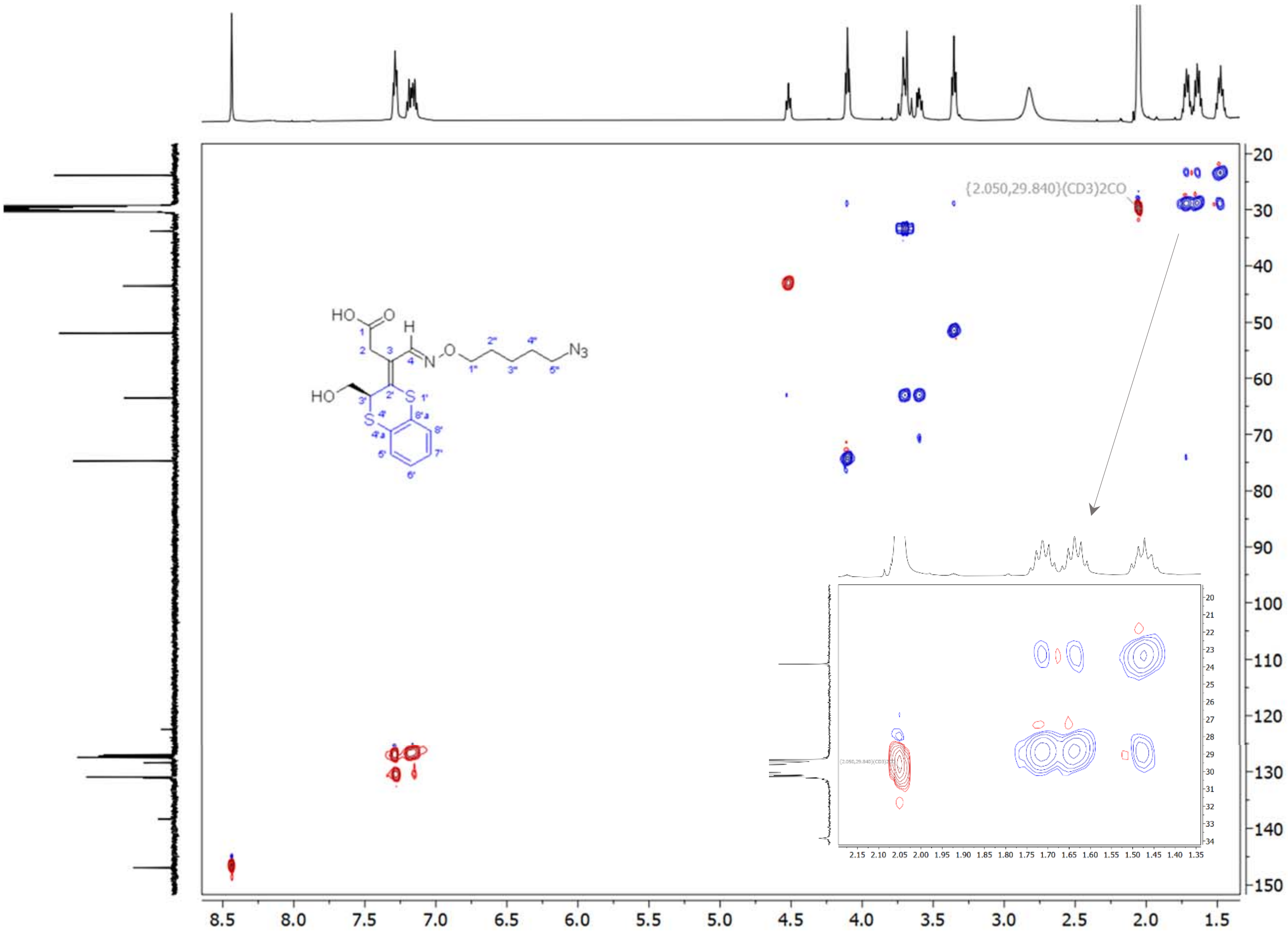

Supplement: Supplementary file 1 — ac4c01631_si_001.pdf [file ac4c01631_si_001.pdf]
